# Effects of mindfulness-based exercise on Parkinson’s disease and Alzheimer’s disease: a systematic review and meta-analysis

**DOI:** 10.3389/fneur.2026.1860499

**Published:** 2026-07-30

**Authors:** Meiwei Zhang, Yu Zhang, Zhibo Zhang, Xiaoye Jia, Shanshan Lei, Nan Bu, Yulin Wang, Yan Bai, Shun Wang

**Affiliations:** 1 Heilongjiang University of Chinese Medicine, Harbin, China; 2 Jiangxi University of Chinese Medicine, Nanchang, China; 3 Institute of Acupuncture and Moxibustion, Heilongjiang Academy of Traditional Chinese Medicine, Harbin, China

**Keywords:** Alzheimer’s disease, meta-analysis, mindfulness-based exercise, motor and cognitive impairments, Parkinson’s disease, qigong, tai chi, yoga

## Abstract

**Objective:**

Currently, there is a lack of robust evidence demonstrating the efficacy of mindfulness-based exercise (MBE) on motor and cognitive outcomes in individuals with Parkinson’s disease (PD) and Alzheimer’s disease (AD), resulting in the absence of standardized and effective MBE treatment protocols. This systematic review and meta-analysis summarizes the available evidence to evaluate the therapeutic effects of MBE on motor and cognitive function in PD and AD.

**Methods:**

For two common neurodegenerative diseases, PD and AD, we searched PubMed, Embase, Cochrane Library, and Web of Science to identify studies published from inception to January 30, 2026. Search terms included neurodegenerative diseases, Parkinson’s disease, Alzheimer’s disease, mindfulness, Tai Chi, yoga, and Qigong. Two independent reviewers assessed the risk of bias of included studies, performed data extraction, and evaluated the evidence. Treatment effects were assessed using the Unified Parkinson’s Disease Rating Scale (UPDRS-III), Timed Up and Go Test (TUG), Berg Balance Scale (BBS), Montreal Cognitive Assessment (MoCA), Mini-Mental State Examination (MMSE), Beck Depression Inventory (BDI), Parkinson’s Disease Sleep Scale (PDSS), Activities of Daily Living (ADL), and Parkinson’s Disease Questionnaire-39 (PDQ-39). Data analysis was performed using Review Manager 5.4 software to evaluate effect sizes and 95% confidence intervals (CIs). Heterogeneity tests were conducted to assess differences in treatment effects among Tai Chi, yoga, and Qigong.

**Results:**

We screened 4,576 articles and identified 28 studies that met the inclusion criteria. Of the included studies, 23 focused on PD and 5 on AD. Only 7 reported adequate allocation concealment, and 2 implemented participant blinding. GRADE assessment indicated moderate certainty of evidence for UPDRS-III, TUG, BBS, MoCA, and MMSE. BDI, PDSS, ADL, and PDQ-39 were rated as low-quality evidence. The pooled results showed significant effects: UPDRS-III (MD: −4.74, 95% CI [−6.78, −2.70], *p* < 0.00001); TUG(MD: −1.63, 95% CI [−2.41, −0.85], *p* < 0.0001); BBS(MD:2.80, 95% CI [1.54, 4.06], *p* < 0.0001); MoCA (MD: 1.93, 95% CI [1.12, 2.74], *p* < 0.00001); MMSE (MD: 2.80, 95% CI [0.32, 5.29], *p* = 0.03); BDI (SMD: −0.18, 95% CI [−0.47, 0.12], *p* = 0.24); PDSS (SMD: −0.31, 95% CI [−0.91, 0.28], *p* = 0.30); ADL (MD: −0.59, 95% CI [−3.49, 2.31], *p* = 0.69); and PDQ-39 (MD: −2.85, 95% CI [−6.36, 0.67], *p* = 0.11).

**Conclusion:**

MBE demonstrated statistically significant improvements in motor function and certain cognitive domains in PD patients, suggesting its potential as a beneficial adjunctive non-pharmacological intervention for ameliorating motor symptoms and delaying cognitive decline, whereas evidence for non-motor symptom improvement remains insufficient. Subgroup analysis further indicated a favorable signal of MBE on cognitive function in AD patients; however, the current evidence is preliminary and warrants validation through additional high-quality studies. Given the inherent challenges in implementing double-blinding due to the nature of MBE interventions, coupled with inadequate allocation concealment, the present findings should be interpreted with caution owing to the substantial risks of performance and selection bias.

## 1 Introduction

Alzheimer’s disease (AD) and Parkinson’s disease (PD) represent two of the most prevalent neurodegenerative disorders. Neurodegenerative diseases represent a heterogeneous group of complex disorders characterized by progressive loss of neurons in the central or peripheral nervous system, synaptic dysfunction, and aseptic neuroinflammation, resulting in gradual deterioration of cognitive or motor function until complete loss of independence in activities of daily living (1–3). These diseases severely affect more than 1.7% of the global population, carry an extremely poor prognosis, and currently lack effective curative treatments (4). Reports indicate approximately 10 million new cases annually, imposing a heavy burden on individuals aged 65 years and older (5, 6). AD is the most common cause of dementia in both industrialized and non-industrialized countries; globally, over 75 million people are suffering from AD, a figure that continues to rise with the accelerating aging population, with an incidence rate of approximately 9.87 cases per 1,000 person-years (7, 8). Clinically, typical AD cases are characterized by progressive decline in memory and other cognitive functions, eventually progressing to dementia (9). PD is the second most common neurodegenerative diseases after AD, affecting more than 6 million people worldwide (10). The prevalence of PD exceeds 10% in populations over 60 years of age, and approximately 40% of PD patients develop cognitive impairment or progress to dementia (11). Motor complications develop in the advanced stages in 80% of treated patients (12). Throughout the disease course, both AD and PD typically exhibit varying degrees of dysfunction in cognitive domains or motor function, imposing substantial burdens on individuals, families, and society (13, 14).

Currently, pharmacological interventions remain the mainstream treatment approach for neurodegenerative diseases in clinical practice. Taking AD as an example, cholinesterase inhibitors and NMDA receptor antagonists serve as first-line clinical medications that can improve cognitive symptoms to some extent but fail to halt or reverse the neurodegenerative process (15, 16). PD is primarily treated with levodopa, which, although effective in alleviating motor symptoms, often induces motor complications with long-term use, such as wearing-off phenomena and dyskinesia, and offers limited benefits for non-motor symptoms including depression, anxiety, and cognitive impairment (17–19). More critically, no pharmacological agent has yet been proven capable of delaying or preventing disease progression in PD and AD, and patients ultimately face inevitable functional decline and reduced quality of life. Against this backdrop, exploring comprehensive intervention strategies that balance symptom management with quality of life enhancement, while demonstrating good feasibility and sustainability, has become a critical priority in PD and AD rehabilitation. MBE has emerged as a novel intervention approach driven by this very need. Mindfulness, originating from ancient Buddhist and yogic meditation techniques, represents a practical and cost-effective practice that activates the parasympathetic nervous system through regulated breathing, enabling individuals to control attention, focus on intentional selection, and observe internal experiences with a non-judgmental attitude (20). Mindfulness practice aims to cultivate a mindful state; these exercises can be formal or informal, fostering an open, non-judgmental, curious, and compassionate awareness of present-moment thoughts, feelings, bodily sensations, and surroundings (21, 22). Following its secularization and manualization in the 1980s, it was introduced into medical settings to help individuals cope with chronic pain and stress (23), and has gained widespread popularity in secular populations across healthcare, education, and workplace settings in recent years (24). Similarly, certain mind–body exercises rooted in Eastern traditions also emphasize achieving mind–body integration through breath and consciousness regulation. MBE, represented by yoga, Tai Chi, and Qigong, serves as a composite intervention that deeply integrates low-intensity physical activity with cognitive training, breath regulation, and mindfulness meditation, demonstrating unique clinical value. At present, MBE is gaining increasing popularity and has gradually been adopted by Western medical and educational systems as a complementary health promotion approach (25). Unlike pure aerobic exercise, MBE emphasizes “mind guiding movement, breath coordinating with mental focus,” representing essentially a highly integrated training process coupling sensorimotor and higher-order cognitive functions. Research suggests that MBE appears capable of modulating sympathetic nervous system (SNS) and hypothalamic–pituitary–adrenal (HPA) axis regulation, reducing stress reactivity, and thereby enhancing overall health and wellbeing (26). Clinical observations have preliminarily confirmed that regular MBE practice can not only improve gait stability and balance function in PD patients but also delay cognitive decline in high-risk AD populations and significantly enhance patients’ quality of life (27, 28). Multiple meta-analyses and systematic reviews have demonstrated that yoga and mindfulness interventions possess mood-improving effects, proving equally effective as conventional antidepressants in treating depressive and anxiety disorders (29, 30). Several reviews conducted in chronic disease populations have supported the notion that mindfulness helps alleviate psychological states such as anxiety, distress, and depression in patients, holding positive significance for improving quality of life (31–33). However, to date, no systematic review has described the efficacy of MBE in neurodegenerative diseases patients. To address this research gap, the present review aims to synthesize evidence examining the efficacy of MBE in patients with neurodegenerative diseases.

However, although neurodegenerative diseases encompass a broad spectrum of disorders, AD and PD were selected as the sole target conditions in the present study, given their highest global prevalence and disease burden, coupled with the fact that existing randomized controlled trial evidence on MBE has been predominantly concentrated on these two diseases. We conducted rigorous literature screening and data synthesis to determine the efficacy of MBE on motor and cognitive impairments associated with PD and AD, aiming to formulate consensus protocols for these impairments and ultimately provide reliable evidence-based support for clinical practice.

## 2 Methods

Systematic reviews and meta-analyses were conducted following the guidelines of the Preferred Reporting Items for Systematic Review and Meta-analysis (PRISMA) (34). The details of the PRISMA checklist can be found in eTable 1 in the Supplementary materials. The protocol for this systematic review has been registered on the International Registry of Prospective Systematic Reviews (PROSPERO) with registration number CRD420261345852.

### 2.1 Literature search and selection

The comprehensive search was conducted across PubMed, Embase, Cochrane Library, and Web of Science from database inception to January 30, 2026. We searched combinations of the following keywords: disease terms (Neurodegenerative Diseases) or (Parkinson’s disease, PD) or (Alzheimer’s disease, AD), and intervention terms (mindfulness) or (Tai Chi) or (yoga) or (Qigong). No filters were applied to the databases. Additionally, we carefully examined meta-analyses related to the study topic and thoroughly reviewed reference lists to ensure comprehensive coverage of all relevant literature (see Text 1 in the Supplementary materials).

Two independent evaluators, ZMW and ZY, employed Endnote 2025 to screen and review the literature, while author WS resolved any disputes arising from the literature screening process. The literature was initially screened based on titles and abstracts, after which eligible full texts of the studies were obtained for secondary screening.

### 2.2 Inclusion and exclusion criteria

We searched only evidence from studies published in peer-reviewed journals, and the PICOS principles were used to determine this study’s inclusion and exclusion criteria; manuscripts published only online were also included in our review.

P (Population):

(1) PD group: Participants were required to meet internationally recognized diagnostic criteria for PD (e.g., UK Parkinson’s Disease Society Brain Bank criteria or Movement Disorder Society clinical diagnostic criteria) and to present with motor dysfunction. Motor impairment severity was stipulated as mild to moderately severe (Hoehn and Yahr stages I–IV).
(2) AD group: Participants were required to belong to one of the following two categories:

1) Patients with confirmed AD: Meeting the National Institute of Neurological and Communicative Disorders and Stroke and the Alzheimer’s Disease and Related Disorders Association criteria, and Statistical Manual of Mental Disorders, 4th Edition/5th Edition diagnostic criteria, or National Institute on Aging and Alzheimer’s Association core clinical criteria; individuals with cognitive impairment were required to a MoCA score below 26 or an MMSE score below 24.
2) Individuals at high risk for AD: Self-reported subjective cognitive decline (SCD) relative to functioning in the prior year, with concomitant presence of one or more cardiovascular risk factors (assessed by the Cerebrovascular Risk Factor Prediction Chart and hematologic testing).

(I) Intervention: Included studies were required to employ MBE as the core intervention in the experimental group. Specifically, this encompassed Tai Chi, yoga, Qigong, and training programs primarily comprising mindfulness meditation. The included MBE interventions were classified into the following three tiers:

Category A: Traditional MBE—Interventions centered on a single traditional mindfulness-based exercise, with mindfulness components embedded within the movement sequence and no additional therapeutic exercise modalities superimposed. This included:

1) Tai Chi: Slow, continuous sequence practice emphasizing intention-guided movement, breath coordination, and dynamic weight shifting;
2) Yoga: Integration of postures, breathing techniques, and meditation/relaxation, including Hatha yoga, Kundalini yoga, and power yoga;
3) Qigong: Practices centered on regulating the body, breath, and mind, including traditional forms such as Wu Qin Xi, Ba Duan Jin, and Liu Zi Jue;
4) Walking meditation: Slow-paced walking with mindfulness awareness as the core component, without adjunctive aerobic endurance or resistance strength objectives.

Category B: Mindfulness training—Standardized mindfulness-based programs incorporating chair yoga.

Category C: Combined MBE—Interventions grounded in mindfulness or traditional MBE but systematically combined with additional therapeutic exercise components (e.g., resistance training, balance training).

C (Comparison): Interventions for the control group included usual care, routine health education, or physical therapy.

O (Outcomes): Motor symptoms and cognitive function were designated as primary outcomes, while secondary outcomes encompassed psychological symptoms, sleep, activities of daily living, and health-related quality of life: (1) Unified Parkinson’s Disease Rating Scale (UPDRS-III); (2) Timed Up and Go Test (TUG); (3) Berg Balance Scale (BBS); (4) Montreal Cognitive Assessment (MoCA); (5) Mini-Mental State Examination (MMSE); (6) Beck Depression Inventory (BDI); (7) Parkinson’s Disease Sleep Scale (PDSS); (8) Activities of Daily Living (ADL); (9) Parkinson’s Disease Questionnaire-39 (PDQ-39).

S (Study Design): All included studies were randomized controlled trials.

The exclusion criteria were duplicate studies, animal studies, reviews, conferences, Non-English studies, and studies that were not RCTs were excluded.

### 2.3 Data extraction

Two independent reviewers (ZMW and ZY) extracted data from eligible articles, including publication year, data source, gender, age, education level, disease duration, intervention methods, and outcome measures. For unavailable data, we contacted authors via telephone and email. Outcome results for each study were extracted as mean ± standard deviation (Mean ± SD). When studies reported data in different formats, such as quartiles or confidence intervals, conversions were performed according to the *Cochrane Handbook* (35). For studies reporting effect estimates graphically, we used the web-based graph digitization tool^1^ to estimate effect sizes from figures. When a study assessed outcomes at multiple time points, we selected the immediate post-treatment data. Data were cross-checked to minimize potential errors, and disagreements were resolved through discussion with the corresponding author (WS).

### 2.4 Quality assessment

Two reviewers (ZY and ZMW) assessed the methodological quality of included studies using the Cochrane Risk of Bias Assessment Tool (36). This tool covers seven key sources of bias, including selection bias, performance bias, detection bias, attrition bias, reporting bias, and other bias. Each article was classified as “low risk,” “high risk,” or “unclear risk” for each type of bias. We used the Grading of Recommendations Assessment, Development and Evaluation (GRADE) approach to evaluate the quality of evidence for cognitive and mood symptoms. Quality assessment was conducted according to the GRADE handbook (37). The GRADEpro Guideline Development Tool (GDT) was used to generate the assessment results. In cases of disagreement, the corresponding author served as arbitrator.

### 2.5 Statistical analysis

All meta-analyses were performed using Review Manager 5.4 software. For continuous variables, mean difference (MD) or standardized mean difference (SMD) with 95% confidence intervals (CIs) were calculated. The *I*^2^ statistic and *p*-values were used to assess heterogeneity among studies. Significant heterogeneity was considered present if *I*^2^ > 50% or *p* < 0.1, indicating substantial variation across studies. A random-effects model was applied when *I*^2^ > 50%; otherwise, a fixed-effects model was used for data analysis. Subgroup or sensitivity analyses were conducted when necessary to explore sources of significant heterogeneity. Sensitivity analyses were performed using the leave-one-out method to determine the robustness and reliability of the pooled results. As more than ten articles were included in this review, publication bias was assessed using funnel plots.

## 3 Results

### 3.1 Study characteristics

**Figure 1:**
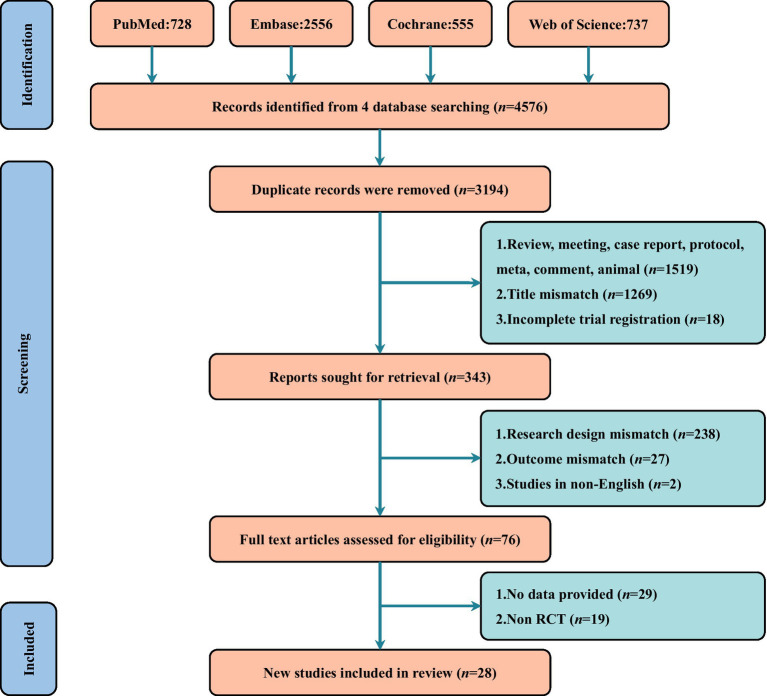
Flowchart of the screening process.

According to the predefined search strategy, a total of 4,576 articles were retrieved (PubMed = 728, Embase = 2,556, Cochrane = 555, Web of Science = 737). After excluding 1,382 duplicate articles, 3,118 articles that did not meet the inclusion and exclusion criteria were excluded through careful review of titles and abstracts. Following comprehensive full-text review of 76 articles, studies with unavailable data or low quality were excluded. Ultimately, 28 articles meeting the study criteria were included (Figure 1). Table 1 summarizes the characteristics of the included studies (detailed characteristics and eligibility assessment can be seen in Supplementary Table 2).

**Table 1:** Characteristics of the included studies.

References	Years	Country	Age (Y)	Sex (M/F)	Disease duration (Y)	Education level (Y)	Sample	Intervention	Duration of treatment	Measured outcome
Adrienne et al. (28)	2024	USA	C:67.54 ± 9.30 E:65.45 ± 9.11	all women	NR	C:15.72 ± 1.99 E:16.15 ± 1.90	C:37 E:26	C:MET E:Kundalini Yoga	12 weeks; 1 session/week; 60-min/day; a 12-min daily homework practice	BDI
Beatrix et al. (60)	2022	USA	C:64.0 ± 6.48 E:60.9 ± 6.66	all women	NR	C:16.1 ± 1.66 E:15.7 ± 2.06	C:11 E:11	C:MET E:Kundalini Yoga	12 weeks; 1 session/week; 60-min/day; a 12-min daily homework practice	BDI
Choi et al. (38)	2016	Korea	C:65.54 ± 6.8 E:60.81 ± 7.6	NR	C:5.2 ± 2.7 E:5.2 ± 2.7	NR	C:9 E:11	C:no intervention E:TTC	12 weeks; 3 session/week; 60-min/day	TUG, ADL
Domingo et al. (61)	2016	Spain	65–75/76–85/≥86 28/65/27	54/66	1/2–3/4–5/≥6 9/43/35/33	3/6/12/15 73/13/21/9	C:29 E:41	C:donepezil E:donepezil+MBAS	two years; 3 session/week; 90-min/day	MMSE
Gao et al. (39)	2014	Hong Kong, China	C:68.28 ± 8.53 E:69.54 ± 7.32	C:27/12 E:23/14	C:8.37 ± 8.24 E:9.15 ± 8.58	NR	C:39 E:37	C:no intervention E:TC	12 weeks; 3 session/week; 60-min/day	UPDRS-III, TUG, BBS
Gloria et al. (40)	2018	USA	C:62 ± 7.77 E:65.7 ± 3.86	C:9/7 E:7/9	C:2.9 ± 2.20 E:2.9 ± 2.38	C:17.56 ± 2.31 E:20.2 ± 8.46	C:13 E:12	C:UC E:UC + TC	6 months; 2 session/week; 60-min/day; a minimum of 60 min per week outside of class	UPDRS-III, TUG, PDQ-39
Huang et al. (62)	2019	China	C:81.9 ± 6.1 E:81.9 ± 6.0	C:14/26 E:12/28	NR	C:6.1 ± 0.13 E:5.94 ± 1.28	C:38 E:36	C:routine treatments E:routine treatments +TC	10 months; 3 session/week; 20-min/day	MoCA, MMSE
Joe et al. (41)	2013	USA	C:65 ± 7 E:66 ± 11	C:4/2 E:7/8	C:6.83 ± 1.83 E:8.08 ± 5.42	C:15 ± 2 E:16 ± 3	C:6 E:15	C:no intervention E:TC	16 weeks; 3 session/week; 60-min/day	ADL, PDQ-39
Kwok et al. (42)	2019	Hong Kong, China	C:63.5 ± 9.3 E:63.7 ± 8.2	C:28/39 E:37/34	NR	Illiterate C:6 E:19	C:59 E:58	C: SRTE + home-based practice E:Hatha yoga + home-based practice	8 weeks; 1 session/week; 90-min/day; a minimum of 20-min home-based practice twice a week	UPDRS-III, TUG
Kwok et al. (43)	2025	Hong Kong, China	C:63.3 ± 7.5 E:66.9 ± 7.9	C:20/34 E:28/24	NR	Illiterate C:3 E:7	C:54 E:52	C:routine outpatient services E: Hatha yoga	8 weeks; 1 session/week; 90-min/day	UPDRS-III
Lee et al. (44)	2018	Korea	C:65.7 ± 6.4 E:65.8 ± 7.2	C:7/9 E:10/15	C:4.4 ± 3.0 E:4.5 ± 3.3	NR	C:16 E:25	C:routine treatment E:routine treatment + Turo	8 weeks; 2 session/week; 60-min/day	UPDRS-III, BBS, BDI, ADL
Li et al. (45)	2012	USA	C:69 ± 9 E: 68 ± 9	C:39/26 E:45/20	C:6 ± 5 E:8 ± 9	NR	C:65 E:65	C:SE E:TC	24 weeks; 2 session/week; 60-min/day	UPDRS-III, TUG
Li et al. a (46)	2022	China	C:70 ± 5.59 E:67.57 ± 3.95	C:16/4 E:13/7	C:7.76 ± 4.55 E:6.83 ± 4.09	C:14.55 ± 1.36 E:14.40 ± 1.47	C:20 E:20	C:SE E:WQX	12 weeks; 2 session/week; 90-min/day	UPDRS-III, TUG, PDQ-39
Li et al. b (47)	2022	China	C:63.25 ± 6.70 E:65.87 ± 6.13	C:6/10 E:5/10	C:6.13 ± 1.96 E:5.60 ± 1.72	NR	C:16 E:15	C:no intervention E:HQE	12 weeks; 5 session/week; 60-min/day	UPDRS-III, TUG
Li et al. c (48)	2022	China	C:61.9 ± 6.76 E:62.7 ± 5.51	C:19/13 E:17/15	C:4.32 ± 2.46 E:5.91 ± 4.01	C:12.40 ± 2.83 E:13.60 ± 2.71	C:17 E:32	C:no-exercise E:TC	12 months	UPDRS-III, TUG, BBS
Li et al. (49)	2024	China	C:60.48 ± 11.52 E:65.59 ± 9.16	C:10/17 E:13/14	C:7.56 ± 7.95 E:4.59 ± 3.70	C:7.52 ± 3.84 E:7.07 ± 4.67	C:27 E:27	C:usual physical activities E:Baduanjin	4 weeks; 5 session/week; 40-min/day	UPDRS-III, PDQ-39
Lin et al. (63)	2019	Taiwan, China	C:76.8 ± 6.9 E:76.7 ± 11.0	NR	NR	NR	C:10 E:11	C:SE E:STC6FA	8 weeks; 2 session/week; 45-min/day	MMSE
Lin et al. (50)	2023	China	C:64.93 ± 6.76 E:65.60 ± 4.53	C:7/8 E:9/6	NR	NR	C:15 E:15	C:TPT E:TC	12 weeks; 2 session/week; 60-min/day	UPDRS-III, BBS, MoCA, MMSE, PDQ-39
Liu et al. (51)	2016	China	C:62.5 ± 3.13 E:65.84 ± 5.45	C:14/12 E:11/17	NR	NR	C:18 E:23	C:drug therapy + regular daily activities E:drug therapy + HQE	10 weeks; 5 session/week; 60-min/day	TUG
Ni et al. (52)	2016	USA	C:74.9 ± 8.3 E:71.2 ± 6.5	C:4/6 E:11/2	C:5.9 ± 6.2 E:6.9 ± 6.3	NR	C:10 E:13	C:health education classes E:High-Speed Yoga	12 weeks; 2 session/week; 60-min/day	UPDRS-III, TUG, BBS
Son et al. (53)	2018	Korea	<60/60–69/ ≥70C:3/21/6 E:6/17/14	C:9/21 E:14/19	<3/≥3C:24/6 E:21/12	Below middle school C:12 E:16	C:30 E:33	C:UC E:MMBCEP	8 weeks; included 6 sessions; 2 h/1 session	MoCA, PDSS, ADL
Thunga et al. (54)	2022	India	C:68.08 ± 6.2 E:69 ± 8.03	C:7/5 E:8/4	C:3.5 ± 1.44 E:3.58 ± 1.56	NR	C:12 E:12	C:physiotherapy E:Yoga	12 weeks; 2 session/week; 60-min/day	UPDRS-III, BDI, ADL, PDQ-39
Timo et al. (27)	2021	Germany	C:66.50 ± 19.26 E:60.50 ± 24.44	C: 7/9 E:8/6	NR	C:15.50 ± 7.41 E:15.50 ± 8.15	C:16 E:14	C:waitlist-control E:IPSUM	8 weeks; 1 session/week; 2 h/day	UPDRS-III, BDI, PDSS, PDQ-39
Wan et al. (55)	2021	China	C:67.03 ± 7.47 E:64.95 ± 7.83	C:11/9 E:8/12	C:3.25 ± 1.73 E:3.63 ± 1.52	NR	C:20 E:20	C:routine medicine E:routine medicine + HQE	12 weeks; 4 session/week; 60-min/day	TUG
Wang et al. (56)	2022	China	C:67.95 ± 4.86 E:68.83 ± 4.35	C:10/12 E:7/16	C:6.09 ± 3.85 E:6.63 ± 4.01	C:11.86 ± 2.64 E:13 ± 2.39	C:22 E:23	C:SE E:WQX	24 weeks; 3 session/week; 90-min/day	UPDRS-III, TUG, MoCA, PDSS, PDQ-39
Witoon M et al. (57)	2022	Thailand	C:61.3 ± 10.5 E:61.3 ± 7.8	C:7/9 E:7/10	C:4.6 ± 4.5 E:4.1 ± 3.1	NR	C:16 E:17	C:standard treatment E:standard treatment + walking meditation	12 weeks; 3 session/week; 60-min/day	UPDRS-III, TUG
Xiao et al. (58)	2016	China	C:66.52 ± 2.13 E:68.17 ± 2.27	C:34/14 E:33/15	C:6.15 ± 2.63 E:5.45 ± 3.61	NR	C:44 E:45	C:30-min walking E:30-min walking + Baduanjin	6 months; 4 session/week; 45-min/day	UPDRS-III, TUG, BBS, PDSS
Yin et al. (59)	2025	China	C:58.15 ± 7.09 E:58.80 ± 8.61	C:12/14 E:12/13	C:6.15 ± 4.47 E:6.05 ± 3.07	NR	C:26 E:25	C:original medications + rehabilitation program E:original medications + Liuzijue	12 weeks; 5 session/week; 30-min/day	UPDRS-III, PDQ-39

ADL, activities of daily living; BBS, berg balance scale; BDI, beck depression inventory; C, control group; E, experimental group; F, female; HQE, health qigong exercise; IPSUM, insight into Parkinson’s disease symptoms by using mindfulness; M, male; MBAS, mindfulness-based Alzheimer’s stimulation; MET, memory enhancement training; MI, music intervention; MMSE, minimum mental state examination; MMBCEP, mindfulness meditation-based complex exercise program; MoCA, Montreal cognitive assessment; PDQ-39, Parkinson’s disease questionnaire-39; PDSS, Parkinson’s disease sleep scale; SE, stretching exercise; SRTE, stretching and resistance training exercise; STC6FA, simplified tai chi 6-form apparatus; TC, tai chi; TPT, traditional physical therapy; TTC, therapeutic tai chi; TUG, timed up and go test; UC, usual care; UPDRS-III, unified Parkinson’s disease rating scale; WQX, Wu Qin Xi; Y, year.

The 28 RCT articles published between 2012 and 2025 comprised a total of 1, 429 participants. All 28 studies employed MBE. 23 articles were related to PD (27, 38–59), and 5 articles were related to AD (28, 60–63). Tai Chi was used in articles (38–41, 45, 48, 50, 62, 63); yoga was used in articles (28, 42, 43, 52, 54, 60); Qigong was used in articles (44, 46, 47, 49, 51, 55, 56, 58, 59); Walking meditation was used in articles (57); Mindfulness training was used in articles (27, 61); Combined MBE was used in articles (53).

PD involved 9 outcome measures: UPDRS-III, TUG, BBS, MoCA, MMSE, BDI, PDSS, ADL, and PDQ-39. AD involved 3 outcome measures: MoCA, MMSE, and BDI. Specifically, 18 articles used the UPDRS-III scale (27, 39, 40, 42–50, 52, 54, 56–59), 14 articles used the TUG scale (38–40, 42, 45–48, 51, 52, 55–58), 6 articles used the BBS scale (39, 44, 48, 50, 52, 58), 4 articles used the MoCA scale (50, 53, 56, 62), 4 articles used the MMSE scale (50, 61–63), 5 articles used the BDI scale (27, 28, 44, 54, 60), 4 articles used the PDSS scale (27, 53, 56, 58), 5 articles used the ADL scale (38, 41, 44, 53, 54), and 9 articles used the PDQ-39 scale (27, 40, 41, 46, 49, 50, 54, 56, 59).

### 3.2 Risk of bias assessment

**Figure 2:**
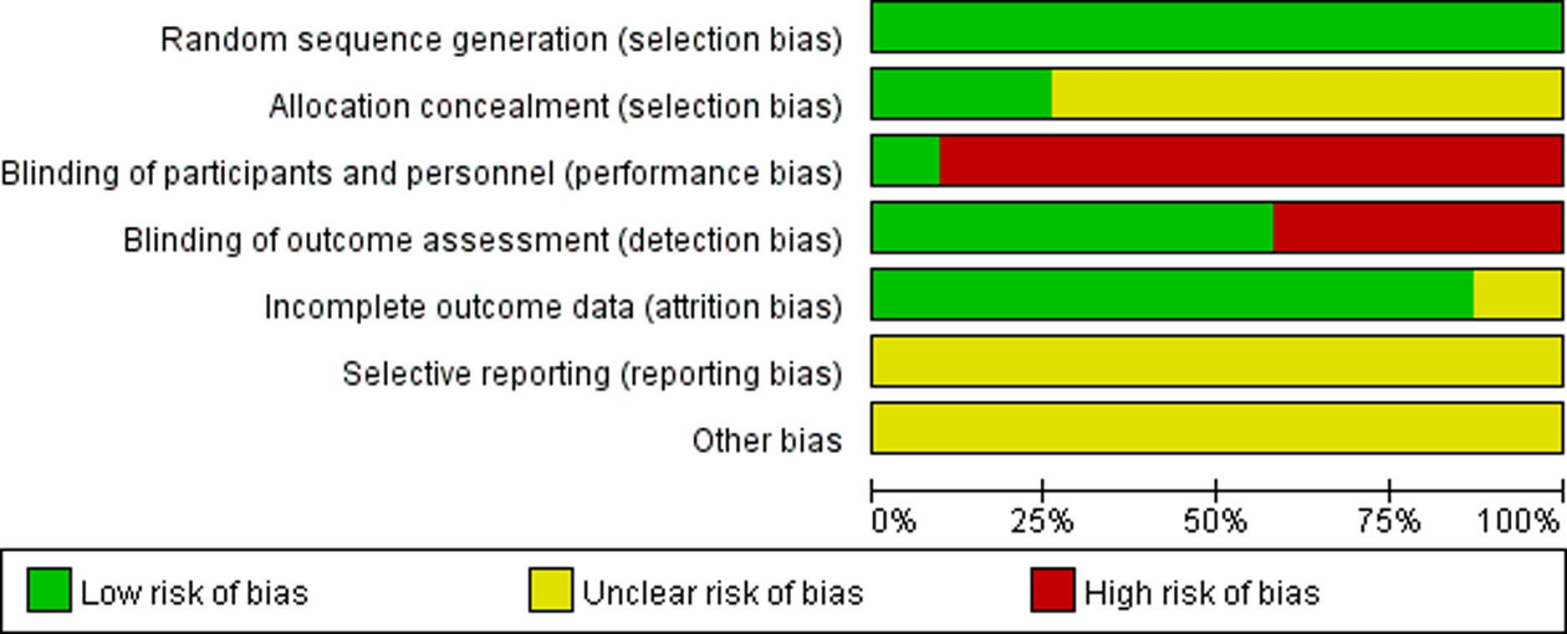
The risk of bias in the included studies.

**Figure 3:**
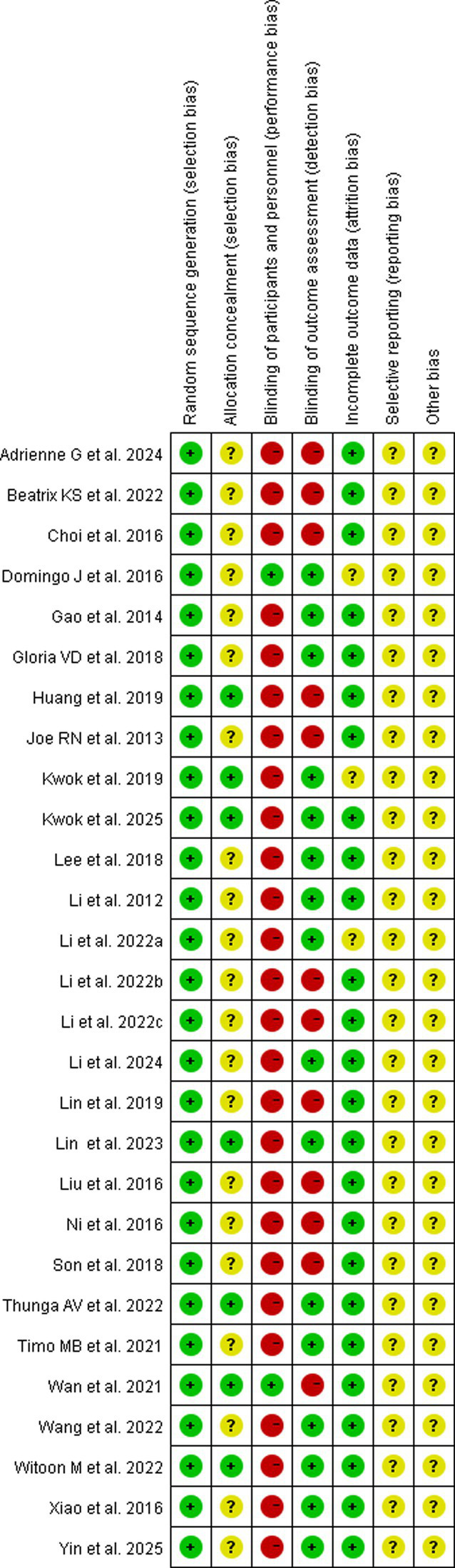
Risk of bias summary: each risk of bias item for each included study.

All 28 included articles provided detailed descriptions of the randomization procedures. 7 studies employed adequate allocation concealment methods, such as using opaque envelopes. 21 studies did not specify the allocation method and were therefore considered “unclear risk.” 2 studies implemented participant blinding, and 16 studies implemented assessor blinding. Consequently, studies without blinding were regarded as high risk (Figures 2, 3).

### 3.3 Motor function

**Figure 4:**
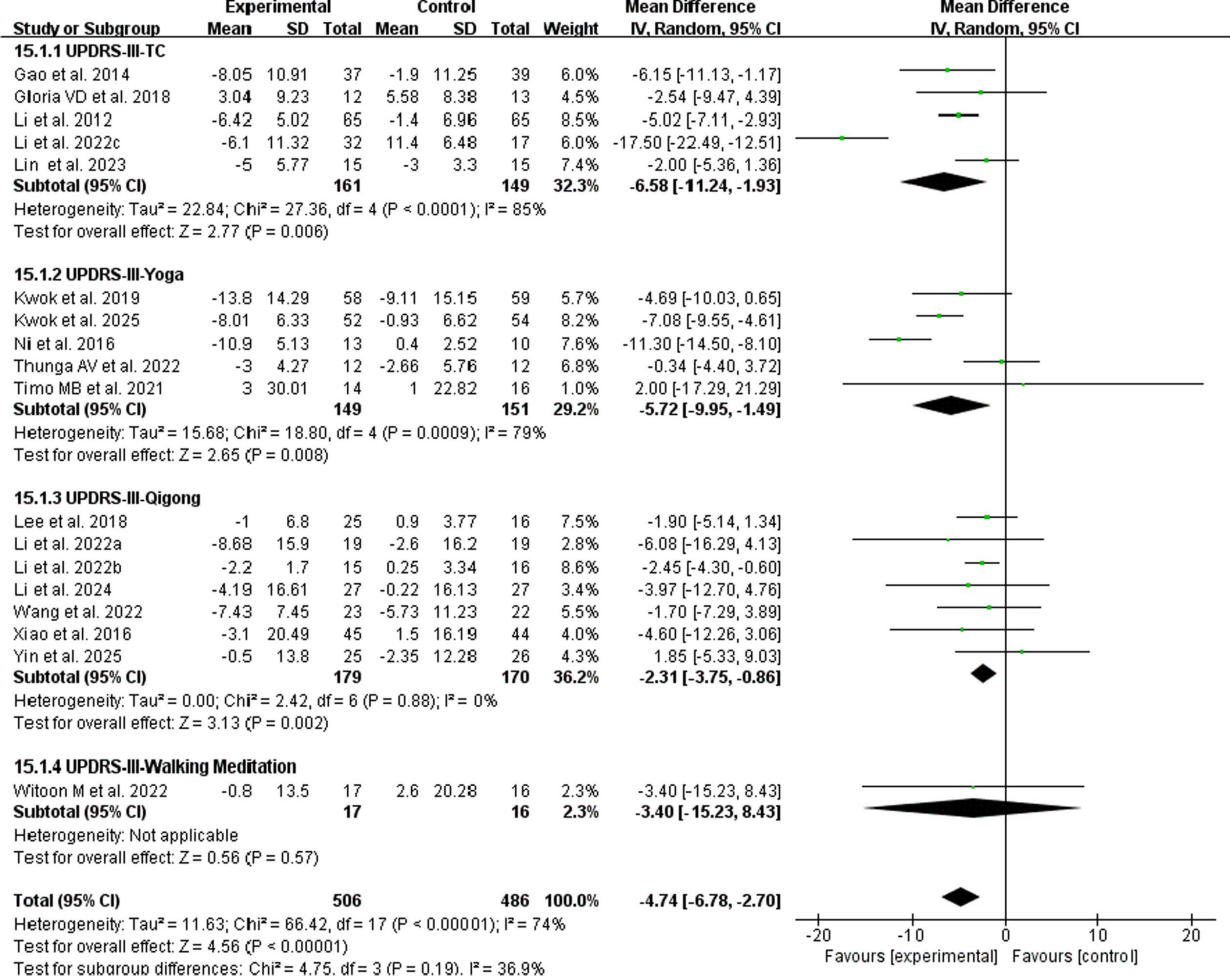
Forest plot of MBE for UPDRS-III.

Figure 4 presents the forest plot for the UPDRS-III analysis. Data on motor function outcomes were provided by 18 studies involving 992 participants. Overall, compared with the control group, MBE significantly improved motor impairments caused by PD (MD: −4.74, 95% CI [−6.78, −2.70], *p* < 0.00001). Further subgroup analyses revealed that Tai Chi demonstrated positive effects on motor impairment improvement (MD: −6.58, 95% CI [−11.24, −1.93], *p* = 0.006); yoga demonstrated positive effects on motor impairment improvement (MD: −5.72, 95% CI [−9.95, −1.49], *p* = 0.008); and Qigong demonstrated positive effects on motor impairment improvement (MD: −2.31, 95% CI [−3.75, −0.86], *p* = 0.002). In contrast, walking meditation did not show significant effects on motor impairment improvement (MD: −3.40, 95% CI [−15.23, 8.43], *p* = 0.57). Sensitivity analysis using the leave-one-out method showed no significant changes in effect sizes or heterogeneity, indicating that the pooled results were robust (see Supplementary Figure 1).

**Figure 5:**
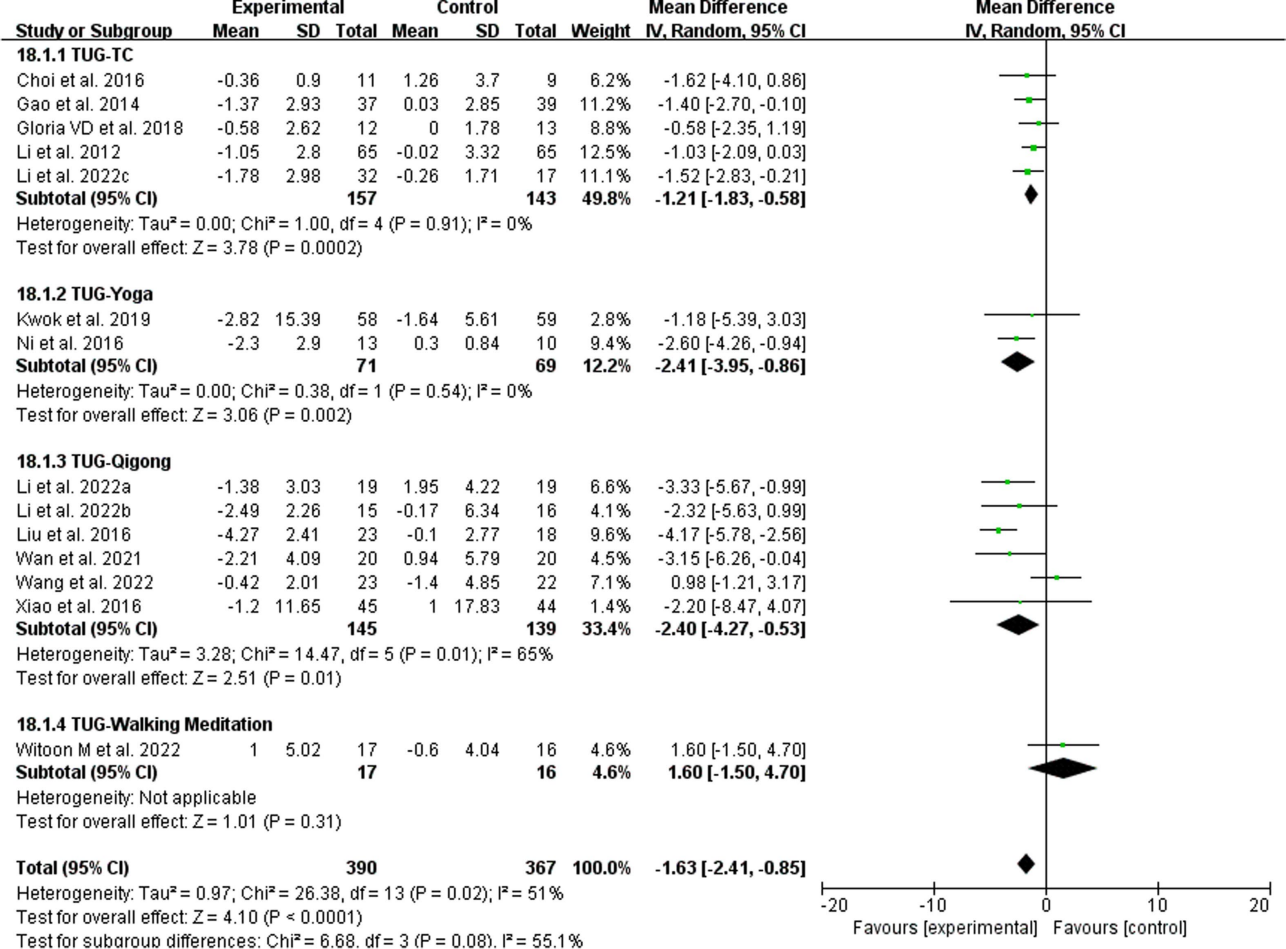
Forest plot of MBE for TUG.

Figure 5 displays the forest plot for the TUG, another motor function assessment scale. The pooled results from 14 studies (757 participants) demonstrated that MBE significantly reduced TUG scores, consistent with the aforementioned positive findings, indicating that MBE contributes to improved motor function in patients with PD (MD: −1.63, 95% CI [−2.41, −0.85], *p* < 0.0001). In further subgroup analyses, we found that Tai Chi, yoga, and Qigong showed similar results in reducing TUG scores (MD: −1.21, 95% CI [−1.83, −0.58], *p* = 0.0002; MD: −2.41, 95% CI [−3.95, −0.86], *p* = 0.002 and MD: −2.40, 95% CI [−4.27, −0.53], *p* = 0.01, respectively). Similarly, walking meditation did not demonstrate significant effects on motor impairment improvement (MD: 1.60, 95% CI [−1.50, 4.70], *p* = 0.31). Sensitivity analysis using the leave-one-out method showed no significant changes in effect sizes or heterogeneity, indicating that the pooled results were robust (see Supplementary Figure 2).

**Figure 6:**
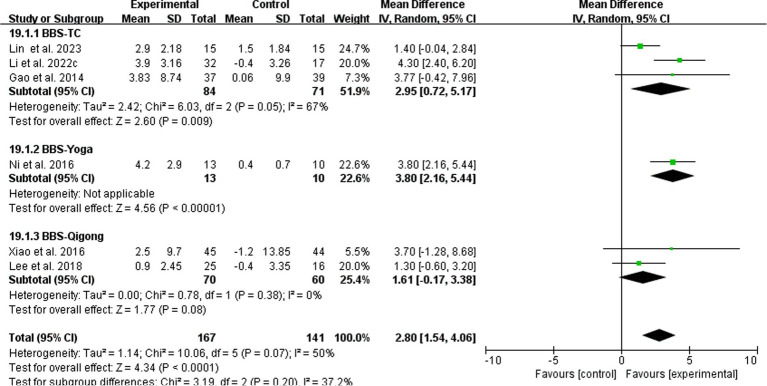
Forest plot of MBE for BBS.

Figure 6 presents the forest plot for the BBS, a balance function assessment scale. Data were provided by 6 studies involving 308 participants. The results demonstrated that MBE had significant effects on improving balance function in patients with PD (MD: 2.80, 95% CI [1.54, 4.06], *p* < 0.0001). Further subgroup analyses revealed that Tai Chi demonstrated positive effects on balance impairment improvement (MD: 2.95, 95% CI [0.72, 5.17], *p* = 0.009); and yoga demonstrated positive effects on balance impairment improvement (MD: 3.80, 95% CI [2.16, 5.44], *p* < 0.00001); whereas Qigong did not show significant effects on balance impairment improvement (MD: 1.61, 95% CI [−0.17, 3.38], *p* = 0.08). Sensitivity analysis using the leave-one-out method showed no significant changes in effect sizes or heterogeneity, indicating that the pooled results were robust (see Supplementary Figure 3).

### 3.4 Cognitive function

**Figure 7:**
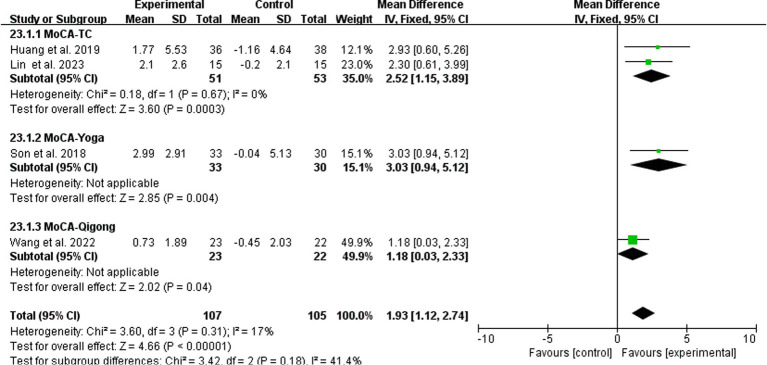
Forest plot of MBE for MoCA.

**Figure 8:**
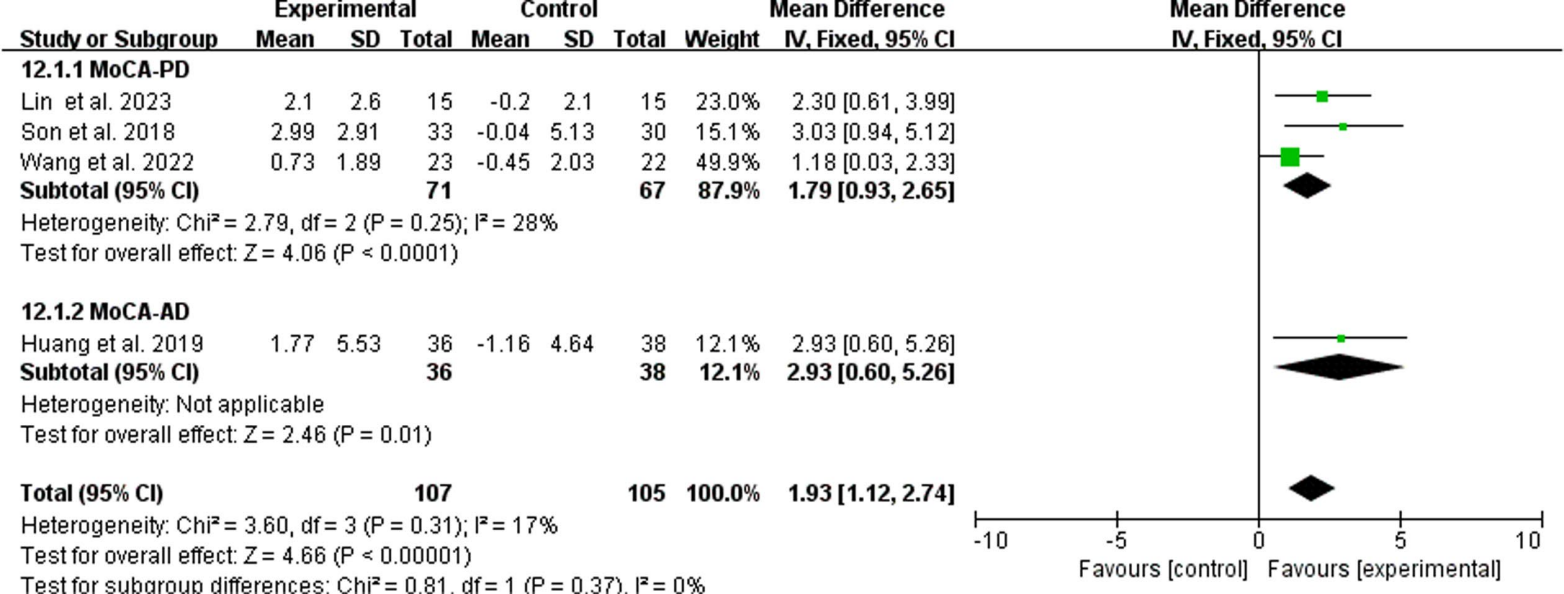
Forest plot of MBE for MoCA.

Figure 7 presents the forest plot for cognitive function assessed by the MoCA. Four studies (212 participants) used the MoCA to evaluate participants’ cognitive function. The pooled results from these four studies demonstrated that MBE had significant effects on improving cognitive function in patients with PD and AD (MD: 1.93, 95% CI [1.12, 2.74], *p* < 0.00001). In further subgroup analyses, we found that Tai Chi, yoga, and Qigong showed positive effects in improving MoCA scores (MD: 2.52, 95% CI [1.15, 3.89], *p* = 0.0003; MD: 3.03, 95% CI [0.94, 5.12], *p* = 0.004; and MD: 1.18, 95% CI [0.03, 2.33], *p* = 0.04, respectively). Results from another subgroup analysis showed that MBE had significant effects on cognitive impairment improvement in patients with PD (MD: 1.79, 95% CI [0.93, 2.65], *p* < 0.0001); similarly, MBE also demonstrated significant effects on cognitive impairment improvement in patients with AD (MD: 2.93, 95% CI [0.60, 5.26], *p* = 0.01) (Figure 8). Sensitivity analysis using the leave-one-out method showed no significant changes in effect sizes or heterogeneity, indicating that the pooled results were robust (see Supplementary Figure 4).

**Figure 9:**
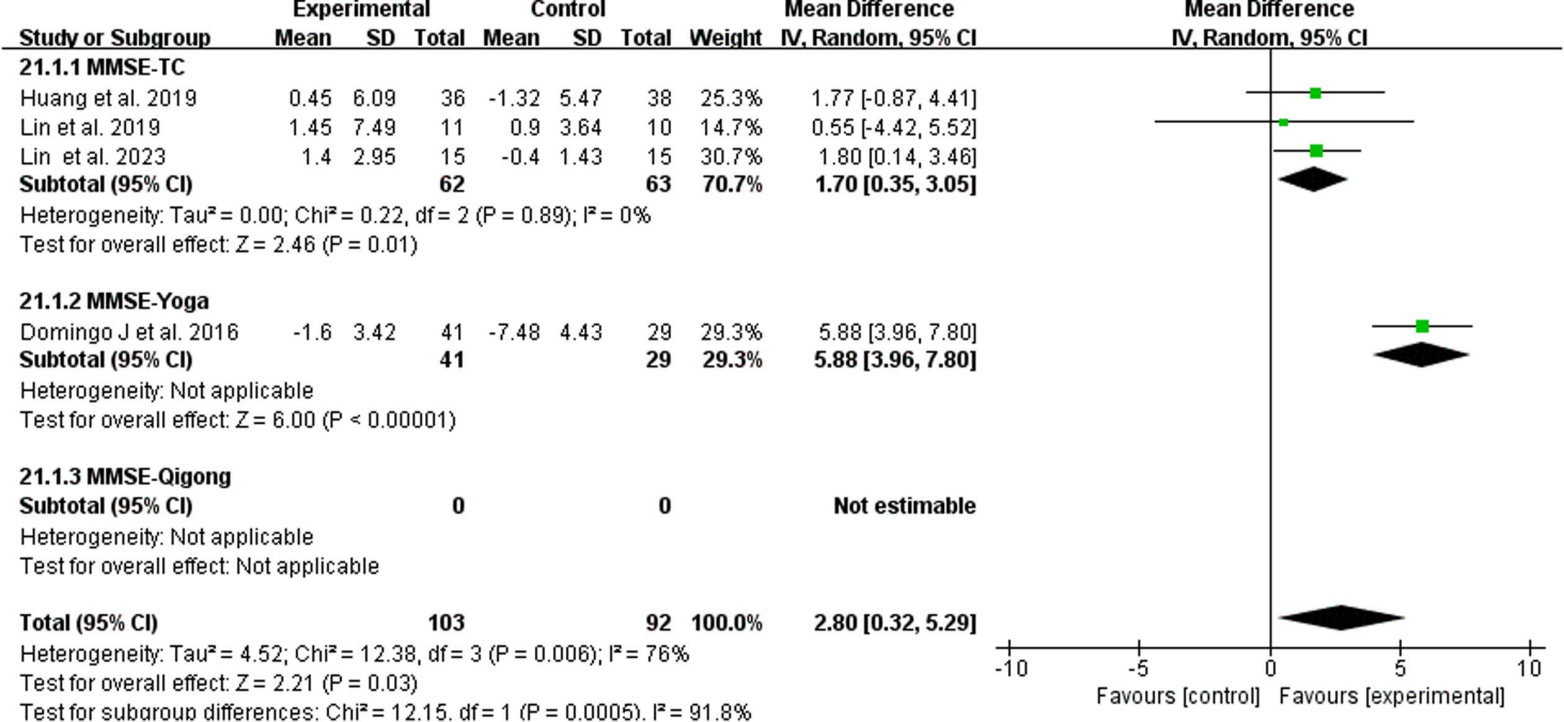
Forest plot of MBE for MMSE.

**Figure 10:**
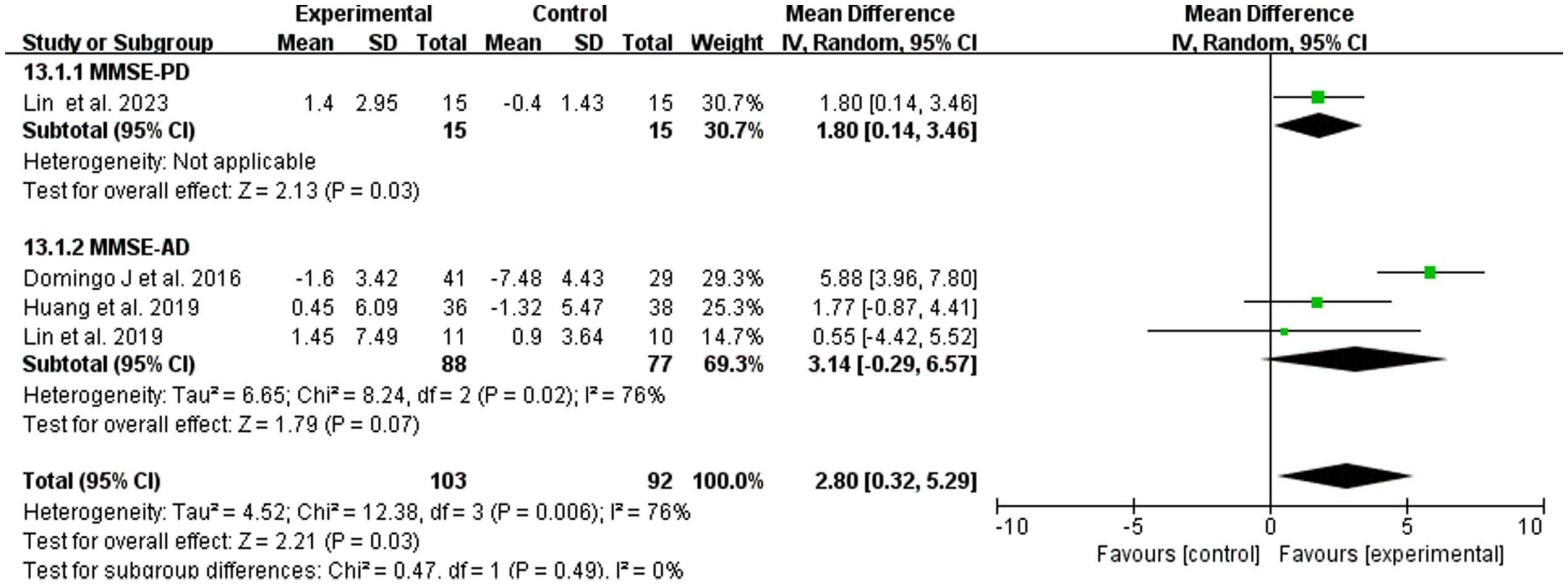
Forest plot of MBE for MMSE.

Figure 9 presents the forest plot for the MMSE, another cognitive function assessment scale. The pooled results from 4 studies (195 participants) demonstrated that MBE significantly improved MMSE scores, consistent with the aforementioned positive findings, indicating that MBE contributes to improved cognitive function in patients with PD and AD (MD: 2.80, 95% CI [0.32, 5.29], *p* = 0.03). Interestingly, in further subgroup analyses, we found that Tai Chi and yoga showed similar results in improving MMSE scores (MD: 1.70, 95% CI [0.35, 3.05], *p* = 0.01; and MD: 5.88, 95% CI [3.96, 7.80], *p* < 0.00001, respectively). In another subgroup analysis, MBE showed pronounced effects on cognitive impairment improvement in patients with PD (MD: 1.80, 95% CI [0.14, 3.46], *p* = 0.03). In contrast, MBE did not show significant effects on cognitive impairment improvement in patients with AD (MD: 3.14, 95% CI [−0.29, 6.57], *p* = 0.07; Figure 10). However, after excluding the study by Domingo J et al., significant fluctuations were observed in the effect size and heterogeneity of the pooled MMSE results (see Supplementary Figure 5).

### 3.5 Psychological symptoms

**Figure 11:**
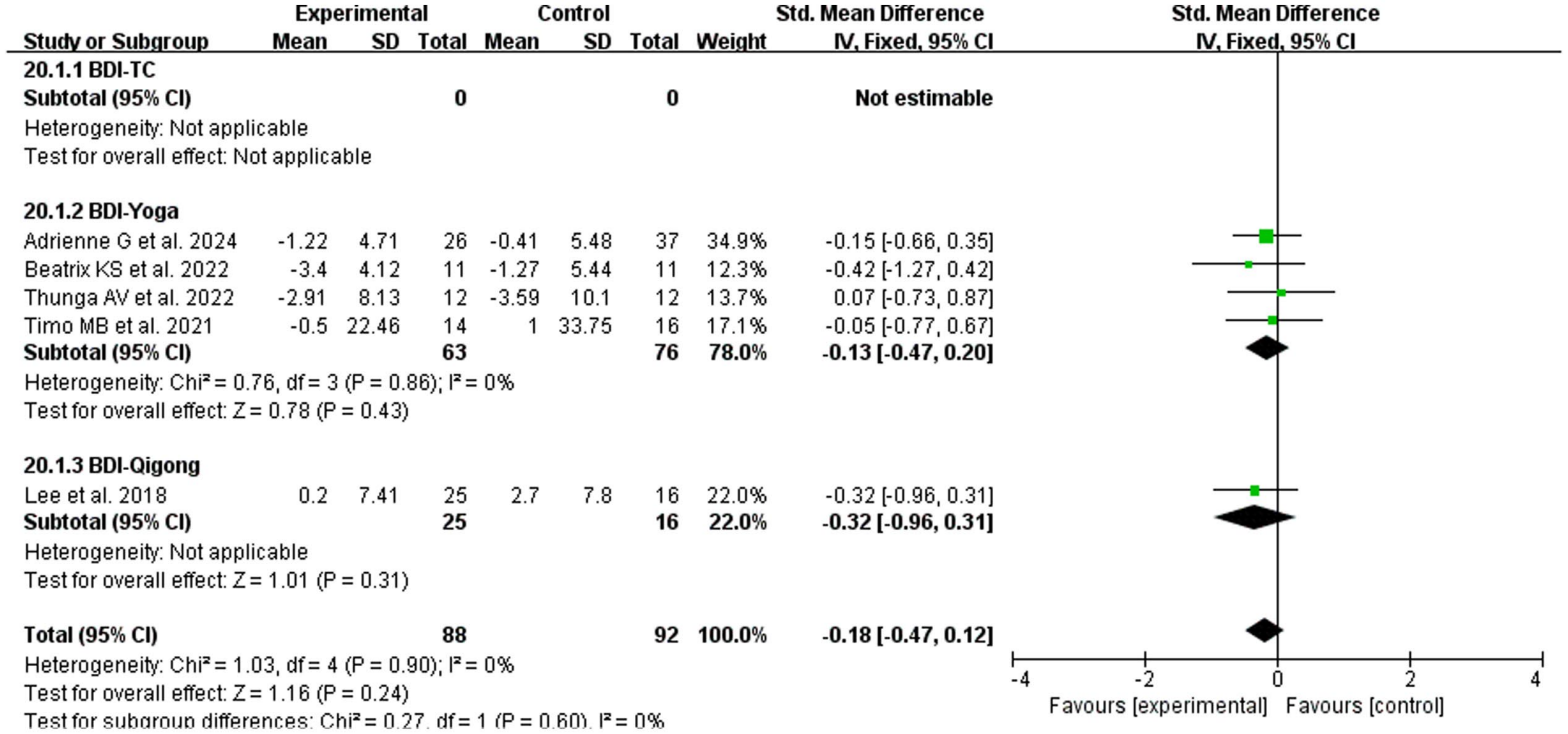
Forest plot of MBE for BDI.

**Figure 12:**
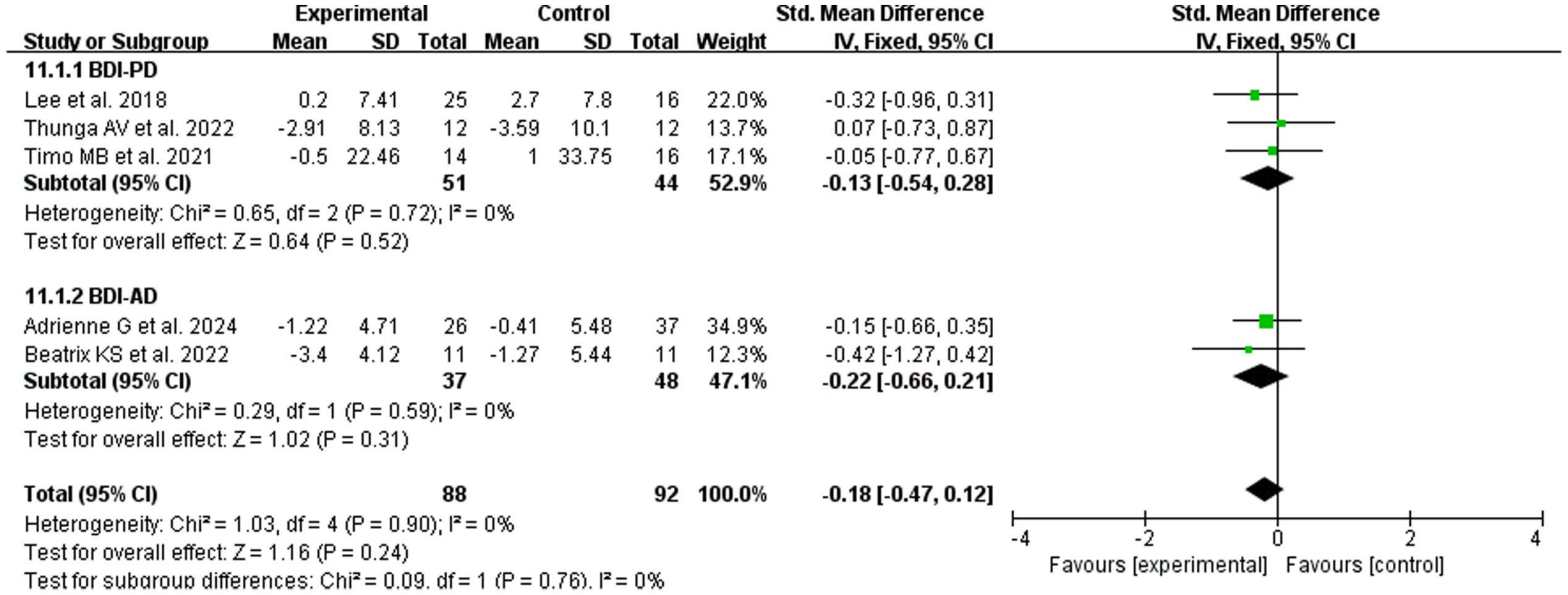
Forest plot of MBE for BDI.

Figure 11 presents the forest plot for depression scores assessed by the BDI. Five studies (180 participants) used the BDI to evaluate depressive symptoms in participants. The pooled results from these five studies demonstrated that MBE did not significantly improve depressive symptoms in patients with PD and AD (SMD: −0.18, 95% CI [−0.47, 0.12], *p* = 0.24). Further subgroup analyses indicated that neither yoga nor Qigong showed favorable performance in improving BDI scores, regardless of whether for patients with PD or AD (Figure 12). Sensitivity analysis using the leave-one-out method showed no significant changes in effect sizes or heterogeneity, indicating that the pooled results were robust (see Supplementary Figure 6).

### 3.6 Sleep

**Figure 13:**
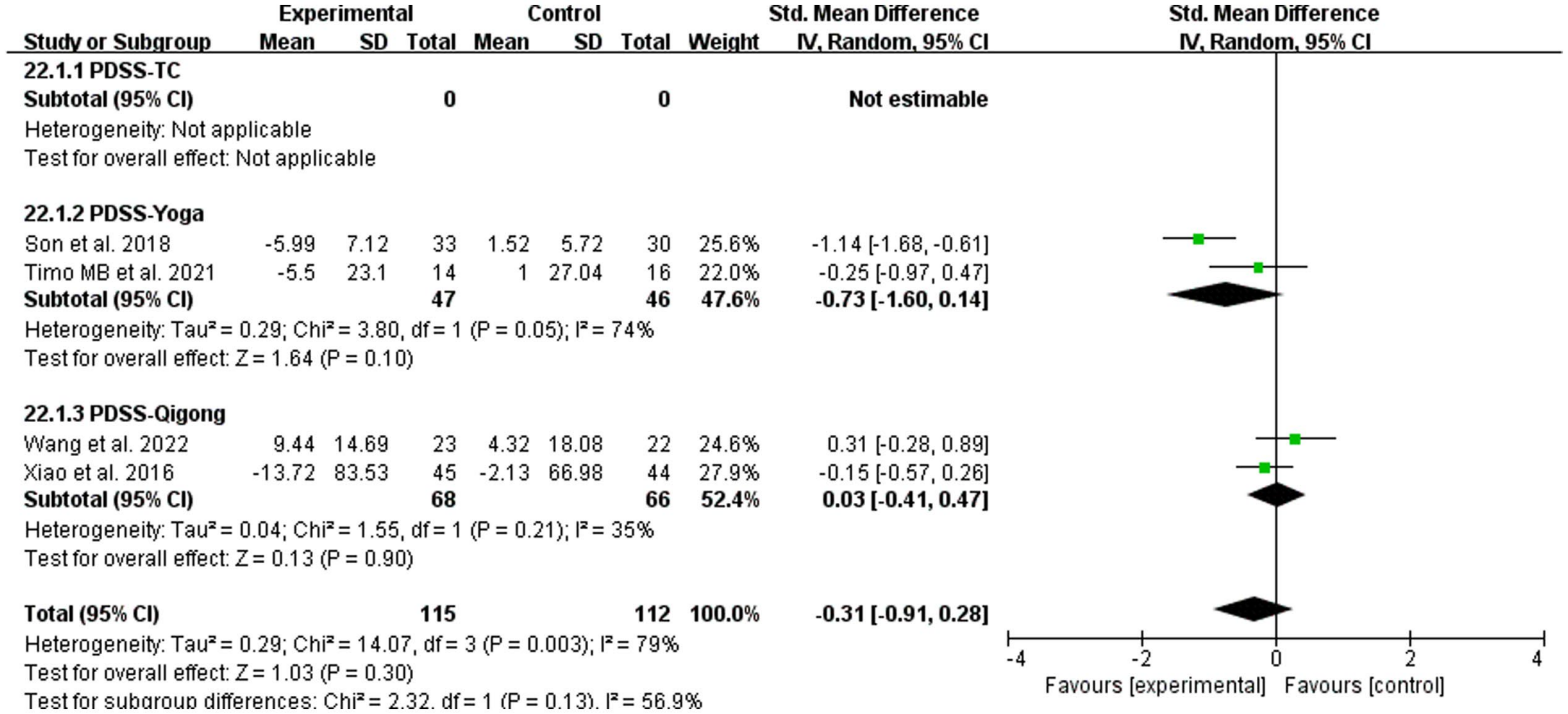
Forest plot of MBE for PDSS.

Figure 13 presents the forest plot for the PDSS. Four studies (212 participants) used the PDSS to evaluate sleep function in participants. The pooled results from these four studies demonstrated that MBE did not significantly improve sleep symptoms in patients with PD (SMD: −0.31, 95% CI [−0.91, 0.28], *p* = 0.30). Further subgroup analyses indicated that neither yoga nor Qigong showed favorable performance. However, after excluding the study by Son et al., significant fluctuations were observed in the effect size and heterogeneity of the pooled PDSS results (see Supplementary Figure 7).

### 3.7 Activities of daily living

**Figure 14:**
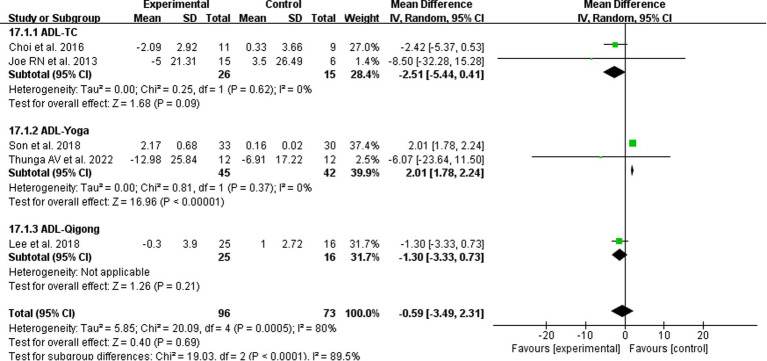
Forest plot of MBE for ADL.

Figure 14 presents the forest plot for ADL. ADL was selected to assess the activities of daily living ability in participants with PD across 5 studies (169 participants). Unfortunately, MBE did not significantly improve ADL scores, and the effect size of MBE intervention was not statistically significant (MD: −0.59, 95% CI [−3.49, 2.31], *p* = 0.69). Interestingly, in further subgroup analyses, we found that yoga demonstrated a beneficial effect on the improvement of activities of daily living (MD: 2.01, 95% CI [1.78, 2.24], *p* < 0.00001), whereas neither Tai Chi nor Qigong showed significant effects (MD: −2.51, 95% CI [−5.44, 0.41], *p* = 0.09; and MD: −1.30, 95% CI [−3.33, 0.73], *p* = 0.21, respectively). However, after excluding the study by Son et al., significant fluctuations were observed in the effect size and heterogeneity of the pooled ADL results (see Supplementary Figure 8).

### 3.8 Health-related quality of life

**Figure 15:**
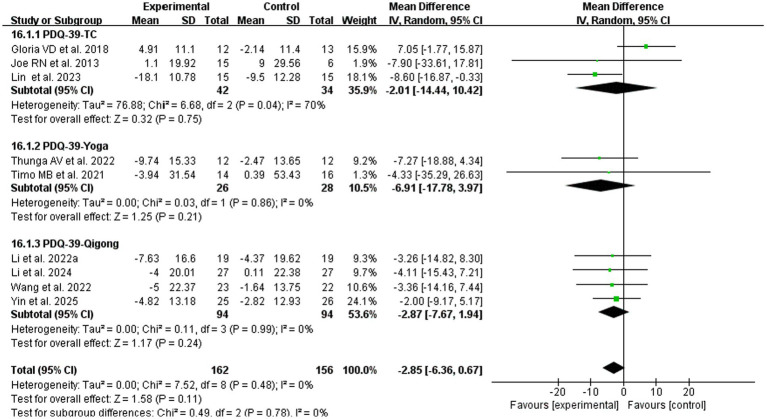
Forest plot of MBE for PDQ-39.

Figure 15 presents the forest plot for the PDQ-39. PDQ-39 was selected to assess health-related quality of life in participants with PD across 9 studies (318 participants). Unfortunately, MBE did not significantly improve PDQ-39 scores, and the effect size of MBE intervention was not statistically significant (MD: −2.85, 95% CI [−6.36, 0.67], *p* = 0.11). At the same time, subgroup analyses indicated that none of the exercise modalities—Tai Chi, yoga, or Qigong—demonstrated favorable performance in improving PDQ-39 scores. Sensitivity analysis using the leave-one-out method showed no significant changes in effect sizes or heterogeneity, indicating that the pooled results were robust (see Supplementary Figure 9).

### 3.9 Publication bias and quality of evidence

**Figure 16:**
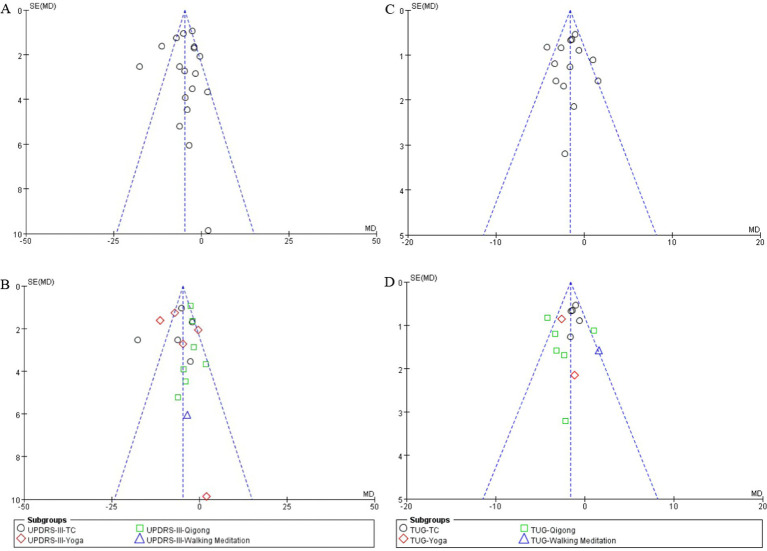
The funnel plot of MBE for UPDRS-III and TUG. **(A)** UPDRS-III; **(B)** UPDRS-III subgroup analysis; **(C)** TUG; **(D)** TUG subgroup analysis.

Publication bias was assessed using funnel plots. Formal funnel plot construction and symmetry evaluation were performed for outcome measures with ≥10 included studies, namely UPDRS-III and TUG. For outcome measures with fewer than 10 included studies, including MoCA, MMSE, BDI, PDSS, ADL, and PDQ-39, funnel plots were considered uninformative and were therefore not generated; accordingly, no definitive conclusions regarding publication bias were drawn for these outcomes. Visual inspection of the funnel plot (Figure 16) revealed that the study points were distributed approximately symmetrically around the top of the funnel, with the majority of studies falling outside the region of statistical significance (*p* < 0.05), suggesting a low likelihood of publication bias.

The GRADE approach was employed to rate the quality of evidence for the primary outcomes. The results indicated that UPDRS-III, TUG, and BBS were rated as moderate-quality evidence due to inadequate allocation concealment. MoCA and MMSE were rated as moderate-quality evidence owing to small sample sizes. BDI, PDSS, ADL, and PDQ-39 were rated as low-quality evidence due to imprecision of effect estimates and inadequate allocation concealment (specific details can be found in Supplementary Table 3).

## 4 Discussion

Before interpreting the findings of this review, it is imperative to emphasize the fundamental constraints imposed by methodological limitations of the included studies on the credibility of the evidence. Among the 28 included RCTs, only 7 implemented adequate allocation concealment, and merely 2 employed participant blinding. Double-blinding is inherently difficult to achieve due to the nature of MBE interventions. Consequently, this spectrum of bias risk implies that all significant effects reported in this review should be interpreted with caution, as they may have been inflated by methodological deficiencies. The following discussions of motor, cognitive, and non-motor symptoms are predicated upon this methodological caveat.

This study summarizes the latest evidence regarding the effects of MBE on motor and cognitive domains in patients with PD and AD. We carefully selected eligible studies from multiple databases according to strict inclusion and exclusion criteria. All included studies were randomized controlled trials, using usual care, routine health education, or physical therapy as controls. Our pooled findings demonstrated that MBE exerts significant positive effects on motor and cognitive domains in patients with PD and AD, which is largely consistent with previous review results. The significant improvements in UPDRS-III scores, TUG test times, and BBS scores indicate that MBE can effectively enhance patients’ motor control capacity, functional mobility, and postural balance, which is highly consistent with the meta-analysis results by Song R et al. (64). This effect may stem from the unique “motor-cognitive dual-task” training properties of MBE—by emphasizing slow, conscious movement control and proprioceptive awareness, the intervention may promote functional reorganization of the prefrontal-basal ganglia-cerebellar circuitry, modulate cortical neuroplasticity, improve dopaminergic neurotransmission, and increase regional cerebral blood flow (65, 66), thereby reducing symptoms such as tremor, rigidity, and bradykinesia. Research has confirmed significant changes in brain structure following mindfulness interventions, with increased gray matter density in brain regions associated with motor function in PD (67). However, it must be emphasized that this improvement was primarily confined to the motor symptom domain and does not imply that MBE could serve as a comprehensive substitute for existing standard treatments for PD.

Notably, the concurrent improvement in MoCA and MMSE scores further supports that cognitive function enhancement may originate from the modulation of prefrontal executive function and default mode network by mindfulness practice. Sustained attentional focus and memory training for complex movement sequences strengthen functional connectivity between the dorsal attention network and frontoparietal network, while improved working memory updating capacity is closely associated with optimized local neural efficiency in the prefrontal cortex (68–70). This observation is consistent with the meta-analysis results by Liu X et al. (71). Interestingly, a certain degree of heterogeneity was present in the pooled results for MoCA and MMSE. In the sensitivity analysis for MMSE, we found that excluding the study by Domingo J and colleagues (61) caused significant changes in the heterogeneity of pooled results, suggesting that the MMSE pooled findings may lack robustness. Through funnel plots, we ruled out the possibility of publication bias. The heterogeneity may derive from differences in intervention protocols and trial duration. Although both mindfulness-based awareness training (MBAT) and Tai Chi fall within the mindfulness domain, they represent fundamentally distinct intervention modalities. MBAT primarily relies on cognitive regulation, whereas Tai Chi indirectly integrates mindfulness elements into physical movement, with a primary focus on mind–body integration and motor coordination. And the included studies spanned a wide range of intervention periods, from the shortest at 8 weeks to the longest at 24 months. Specifically, short-term interventions may primarily induce acute plastic changes in neural functional connectivity, manifested as rapid improvements in attention and executive function (72). In contrast, long-term interventions may be accompanied by structural neuroplastic remodeling, including increased prefrontal cortical gray matter volume and improved white matter integrity (73, 74); this delayed neuroanatomical alteration exhibits fundamentally different patterns of influence on cognitive function compared to short-term effects. Furthermore, over an ultra-long duration of 24 months, the natural progression of diseases leading to continuous dopaminergic neuronal loss and accelerated cortical atrophy may form a dynamic interplay with the compensatory effects of intervention (75, 76), resulting in non-linear trajectories of MMSE score changes across different time points. Therefore, excessive span in study duration may lead to discrepancies in efficacy outcomes, which may partially account for the heterogeneity observed in cognitive results for MBE. However, due to the limited number of included studies, these results should be interpreted with caution. Future research should explicitly define core components and develop reproducible, standardized protocols, employing individual participant data meta-analysis or piecewise linear mixed models to precisely parse the dose–response relationship between intervention duration and cognitive improvement, and identify potential optimal intervention windows.

More intriguingly, we found that the MoCA scale demonstrated significant effects of MBE on cognitive impairment improvement in both PD and AD patients; however, MMSE results indicated that MBE demonstrated beneficial effects on cognitive domains in patients with PD. In contrast, MBE did not demonstrate significant effects on cognitive domains in AD patients. Analysis of underlying reasons suggests that this discrepancy may stem from differences in scale sensitivity. MoCA is more sensitive to the detection of mild cognitive impairment and cognitive domains including executive function, attention, and visuospatial ability, whereas MMSE focuses on orientation, immediate memory, and language ability, exhibiting ceiling effects in identifying early cognitive decline (77). Due to older age and more extensive neurodegeneration, AD patients may have baseline MMSE scores already in the moderate-to-severe range, presenting floor effects that render subtle cognitive improvements from MBE difficult to manifest as statistically significant changes on this scale. In contrast, PD patients exhibit relatively milder cognitive impairment, allowing MMSE to still capture improvement space in orientation and language function. Therefore, although MoCA captured potential improvements in attention and executive function in AD patients, the structural limitations of MMSE combined with more severe baseline cognitive impairment and multi-system pathology in AD patients collectively contributed to the absence of significant effects on this scale. However, given the limited number of included studies, the results lacked statistical power and should be interpreted with caution.

It is also noteworthy that, despite the beneficial effects of MBE on motor symptoms in PD, the pooled effects on BDI, PDSS, ADL, and PDQ-39 did not achieve statistical significance. This may be attributable to several factors. First, non-motor symptoms in PD involve distinct neural circuits (78), and the mechanism by which MBE improves motor control via the prefrontal-basal ganglia circuit may not directly modulate non-motor networks. Second, the intervention dose may have been insufficient to influence non-motor symptoms. While improvements in motor symptoms may manifest within several weeks, alterations in emotional regulation and sleep architecture may require longer intervention durations or higher mindfulness doses. However, after excluding the study by Son and colleagues (53), significant fluctuations were observed in the effect sizes and heterogeneity of the pooled PDSS and ADL results. Through funnel plots, we ruled out the possibility of publication bias. The heterogeneity may derive from differences in trial design protocols. The study by Son et al. was designed as a “complex exercise program,” which essentially constituted a modified protocol grounded in yoga principles and integrated with ball-based balance training. This multimodal intervention differs essentially from pure Tai Chi, yoga, or Qigong in terms of exercise intensity, cognitive load, and neural mechanisms. According to relevant dose–response meta-analyses, different exercise types exhibit significant differences in effects on neurotrophic factors, and the optimal dosage range for mindfulness intervention is 260–340 METs-min/week (79). The combined intervention by Son and colleagues (53) may have exceeded this range, leading to deviation in effect sizes. However, due to the limited number of included studies, these results should be interpreted with caution. Future research should employ more stringent inclusion criteria, restricting intervention types to pure MBE rather than combined interventions, to validate the effect patterns observed in this meta-analysis.

## 5 Conclusion

Through this systematic review and meta-analysis, we observed that MBE demonstrated potential beneficial effects on motor and cognitive domains in patients with PD and AD; however, the pooled effects on BDI, PDSS, ADL, and PDQ-39 did not reach statistical significance. These findings suggest that MBE may serve as a beneficial adjunctive non-pharmacological intervention within comprehensive treatment regimens for PD, particularly for patients presenting with motor complications and early cognitive decline, whereas the evidence for its efficacy in improving non-motor symptoms remains insufficient. In the AD domain, subgroup analyses indicated a favorable signal of MBE on cognitive function in AD patients. Nevertheless, given the limited number of AD-related studies included and the fact that some studies enrolled individuals at high risk for AD rather than confirmed AD cases, the therapeutic efficacy of MBE on cognitive function in AD patients remains inconclusive and should be regarded as a preliminary exploratory direction warranting further validation. Pending additional high-quality evidence, MBE should be considered an adjunctive option within integrated care protocols, with its actual clinical benefit requiring confirmation through larger-scale studies employing more stringent diagnostic criteria. Given the inherent challenges in implementing double-blinding due to the nature of MBE interventions, coupled with inadequate allocation concealment, the present findings should be interpreted with caution owing to the substantial risks of performance and selection bias.

## 6 Limitations

Our current study has several limitations. First, this study included only PD and AD among neurodegenerative diseases; therefore, the conclusions cannot be extrapolated to other neurodegenerative conditions such as Huntington’s disease or amyotrophic lateral sclerosis. Future research targeting specific neurodegenerative diseases is warranted. Second, few included studies employed allocation concealment, leading to an elevated risk of selection bias that may exaggerate intervention effect sizes. Third, due to the inherent nature of MBE interventions, implementing double-blinding presents intrinsic difficulties; participants’ and assessors’ awareness of group allocation may introduce expectancy effects and assessment bias, particularly evident in subjectively reported outcome measures. Fourth, despite the establishment of a hierarchical classification system, the limited number of studies resulted in insufficient statistical power; consequently, these stratified findings are merely suggestive and do not permit definitive conclusions. Moreover, traditional MBE modalities exhibit heterogeneity in movement forms, breathing patterns, pedagogical systems, and school affiliations, which, compounded by variations in intervention intensity and total duration across studies, further complicates effect evaluation and cross-study comparisons. Future research should conduct dose–response meta-analyses to systematically explore the associations between different intervention parameters and outcome measures, thereby elucidating optimal intervention protocols. Finally, the relatively limited sample size of AD patients, combined with the inclusion of both confirmed AD cases and individuals at high risk for AD, may have introduced sample heterogeneity and potential selection bias, thereby affecting the reliability of the results; these findings should therefore be interpreted with caution.

## Data Availability

The original contributions presented in the study are included in the article/Supplementary material, further inquiries can be directed to the corresponding authors.

## Footnotes

1
www.plotdigitizer.sourceforge.net
