# Supplementary material for: Effects of mindfulness-based exercise on Parkinson’s disease and Alzheimer’s disease: a systematic review and meta-analysis
Source: Front Neurol. 2026 Jul 30;17:1860499. doi: 10.3389/fneur.2026.1860499 (PMC13470669; doi:10.3389/fneur.2026.1860499)
Supplement: Supplementary file 1 [file Data_Sheet_1.pdf]

Supplementary Material

eTable 1 PRISMA 2020 Checklist			
**Section and Topic**	**Item #**	**Checklist item**	**Location where item is reported**
**TITLE**			
**Title**	**1**	**Identify the report as a systematic review.**	**Title**
**ABSTRACT**			
**Abstract**	**2**	**See the PRISMA 2020 for Abstracts checklist.**	**Abstract**
**INTRODUCTION**			

**Rationale**	**3**	**Describe the rationale for the review in the context of existing knowledge.**	**Introduction**
**Objectives**	**4**	**Provide an explicit statement of the objective(s) or question(s) the review addresses.**	**Introduction**
**METHODS**			
**Eligibility criteria**	**5**	**Specify the inclusion and exclusion criteria for the review and how studies were grouped for the syntheses.**	**Methods**
**Information sources**	**6**	**Specify all databases, registers, websites, organisations, reference lists and other sources searched or consulted to identify**	**Methods**

		**studies. Specify the date when each source was last searched or consulted.**	
**Search strategy**	**7**	**Present the full search strategies for all databases, registers and websites, including any filters and limits used.**	**Supplementary Material**
**Selection process**	**8**	**Specify the methods used to decide whether a study met the inclusion criteria of the review, including how many reviewers screened each record and each report retrieved, whether they worked independently, and if applicable, details of automation tools used in the process.**	**eTable 2**

**Data collection process**	**9**	**Specify the methods used to collect data from reports, including how many reviewers collected data from each report, whether they worked independently, any processes for obtaining or confirming data from study investigators, and if applicable, details of automation tools used in the process.**	**Methods**
**Data items**	**10a**	**List and define all outcomes for which data were sought. Specify whether all results that were compatible with each outcome domain in each study were sought (e.g. for all measures, time points,**	**Results**

		**analyses), and if not, the methods used to decide which results to collect.**	
	**10b**	**List and define all other variables for which data were sought (e.g. participant and intervention characteristics, funding sources). Describe any assumptions made about any missing or unclear information.**	**Table 1**
**Study risk of bias assessment**	**11**	**Specify the methods used to assess risk of bias in the included studies, including details of the tool(s) used, how many reviewers assessed each study and**	**Methods**

		**whether they worked independently, and if applicable, details of automation tools used in the process.**	
**Effect measures**	**12**	**Specify for each outcome the effect measure(s) (e.g. risk ratio, mean difference) used in the synthesis or presentation of results.**	**Methods**
**Synthesis methods**	**13a**	**Describe the processes used to decide which studies were eligible for each synthesis (e.g. tabulating the study intervention characteristics and comparing against the planned groups**	**Results and eTable 2**

	**for each synthesis (item #5)).**	
**13b**	**Describe any methods required to prepare the data for presentation or synthesis, such as handling of missing summary statistics, or data conversions.**	**Methods**
**13c**	**Describe any methods used to tabulate or visually display results of individual studies and syntheses.**	**Results**
**13d**	**Describe any methods used to synthesize results and provide a rationale for the choice(s). If meta-analysis was performed, describe the model(s),**	**Methods**

		**method(s) to identify the presence and extent of statistical heterogeneity, and software package(s) used.**	
	**13e**	**Describe any methods used to explore possible causes of heterogeneity among study results (e.g. subgroup analysis, meta-regression).**	**Methods**
	**13f**	**Describe any sensitivity analyses conducted to assess robustness of the synthesized results.**	**Methods**
**Reporting bias**	**14**	**Describe any methods used to assess risk of bias due to missing results in a**	**Methods**

**assessment**		**synthesis (arising from reporting biases).**	
**Certainty assessment**	**15**	**Describe any methods used to assess certainty (or confidence) in the body of evidence for an outcome.**	**Methods**
**RESULTS**			
**Study selection**	**16a**	**Describe the results of the search and selection process, from the number of records identified in the search to the number of studies included in the review, ideally using a flow diagram.**	**Results**
	**16b**	**Cite studies that might appear to meet the inclusion criteria, but which were**	**This review focuses on the effects of mindfulness-based exercise on Parkinson's Disease and Alzheimer's Disease, with very specific inclusion criteria. Therefore, studies either**

		excluded, and explain why they were excluded.	clearly meet the criteria or clearly do not, with no ambiguous cases.
Study characteristics	17	Cite each included study and present its characteristics.	Results
Risk of bias in studies	18	Present assessments of risk of bias for each included study.	Results
Results of individual studies	19	For all outcomes, present, for each study: (a) summary statistics for each group (where appropriate) and (b) an effect estimate and its precision (e.g. confidence/credible interval), ideally using structured tables or plots.	Results

**Results of syntheses**	**20a**	**For each synthesis, briefly summarise the characteristics and risk of bias among contributing studies.**	**Results**
	**20b**	**Present results of all statistical syntheses conducted. If meta-analysis was done, present for each the summary estimate and its precision (e.g. confidence/credible interval) and measures of statistical heterogeneity. If comparing groups, describe the direction of the effect.**	**Results**
	**20c**	**Present results of all investigations of possible causes of heterogeneity among**	**Discussion**

		**study results.**	
	**20d**	**Present results of all sensitivity analyses conducted to assess the robustness of the synthesized results.**	**eFigure 1-eFigure 9**
**Reporting biases**	**21**	**Present assessments of risk of bias due to missing results (arising from reporting biases) for each synthesis assessed.**	**Results**
**Certainty of evidence**	**22**	**Present assessments of certainty (or confidence) in the body of evidence for each outcome assessed.**	**Results**
**DISCUSSION**			

**Discussion**	**23a**	**Provide a general interpretation of the results in the context of other evidence.**	**Discussion**
	**23b**	**Discuss any limitations of the evidence included in the review.**	**Discussion**
	**23c**	**Discuss any limitations of the review processes used.**	**Discussion**
	**23d**	**Discuss implications of the results for practice, policy, and future research.**	**Discussion**
**OTHER INFORMATION**			
**Registration**	**24a**	**Provide registration information for the review, including register name and**	**Methods**

**and protocol**		**registration number, or state that the review was not registered.**	
	**24b**	**Indicate where the review protocol can be accessed, or state that a protocol was not prepared.**	**Methods and Protocol access link:** https://www.crd.york.ac.uk/prospero/display_record.php?RecordID=1345852
	**24c**	**Describe and explain any amendments to information provided at registration or in the protocol.**	**This study was conducted in strict accordance with the pre-registered protocol. No modifications were made to the study objectives, methodological design, or analysis plan. The only change involved the registered title, which was revised solely to improve terminological precision without altering the substantive content of the protocol.**
**Support**	**25**	**Describe sources of financial or non-financial support for the review, and the role of the funders or sponsors in the**	**Funding**

		**review.**	
**Competing interests**	**26**	**Declare any competing interests of review authors.**	**Declarations**
**Availability of data, code and other materials**	**27**	**Report which of the following are publicly available and where they can be found: template data collection forms; data extracted from included studies; data used for all analyses; analytic code; any other materials used in the review.**	**Declarations**

Text 1 The complete search strategies for four databases

Pubmed

Search: (("Neurodegenerative Diseases"[Mesh]) OR (Neurodegenerative Disease)) OR (Degenerative Neurologic Disorders)) OR (Degenerative Neurologic Disorder)) OR (Neurologic Disorder, Degenerative)) OR (Neurologic Disorders, Degenerative)) OR (Nervous System Degenerative Diseases)) OR (Neurodegenerative Disorders)) OR (Neurodegenerative Disorder)) OR (Degenerative Diseases, Nervous System)) OR (Degenerative Diseases, Neurologic)) OR (Neurologic Degenerative Disease)) OR (Neurologic Degenerative Conditions)) OR (Degenerative Condition, Neurologic)) OR (Degenerative Conditions, Neurologic)) OR (Neurologic Degenerative Condition)) OR (Neurologic Degenerative Diseases)) OR (Degenerative Neurologic Diseases)) OR (Degenerative Neurologic Disease)) OR (Neurologic Disease, Degenerative)) OR (Neurologic Diseases, Degenerative)) OR (Degenerative Diseases, Central Nervous System)) OR (Degenerative Diseases, Spinal Cord)) OR (Parkinson Disease)) OR (Idiopathic Parkinson Disease)) OR (Idiopathic Parkinson's Disease)) OR (Lewy Body Parkinson Disease)) OR (Lewy Body Parkinson's Disease)) OR (Paralysis Agitans)) OR (Parkinson Disease, Idiopathic)) OR (Parkinson's Disease)) OR (Parkinson's Disease, Idiopathic)) OR (Parkinson's Disease, Lewy Body)) OR (Primary Parkinsonism)) OR (Parkinsonism, Primary)) OR (Alzheimer Syndrome)) OR (Alzheimer-Type Dementia (ATD))) OR (Alzheimer Type Dementia (ATD))) OR (Dementia, Alzheimer-Type (ATD))) OR (Alzheimer's Diseases)) OR (Alzheimer Diseases)) OR (Alzheimers Diseases)) OR (Alzheimer Dementia)) OR (Alzheimer Dementias)) OR (Dementia, Alzheimer)) OR (Alzheimer's Disease)) OR (Dementia, Senile)) OR (Senile Dementia)) OR (Dementia, Alzheimer Type)) OR (Alzheimer Type Dementia)) OR (Senile Dementia, Alzheimer Type)) OR (Alzheimer Type Senile Dementia)) OR (Primary Senile Degenerative Dementia)) OR (Alzheimer Sclerosis)) OR (Sclerosis, Alzheimer)) OR (Dementia, Primary Senile Degenerative)) OR (Dementia, Presenile)) OR (Presenile Dementia)) OR (Acute Confusional Senile Dementia)) OR (Senile Dementia, Acute Confusional)) OR (Alzheimer Disease, Early Onset)) OR (Early Onset Alzheimer Disease)) OR (Presenile Alzheimer Dementia)) OR (Alzheimer Disease, Late Onset)) OR (Late Onset Alzheimer Disease)) OR (Alzheimer's Disease, Focal Onset)) OR (Focal Onset Alzheimer's Disease)) OR (Familial Alzheimer Disease (FAD))) OR (Alzheimer Disease, Familial (FAD))) OR (Familial Alzheimer Diseases (FAD))) AND ((((((((((((((((((("Mindfulness"[Mesh]) OR (Mindfulness Meditation)) OR (Meditation, Mindfulness)) OR (Mindfulness Meditations)) OR (Tai Ji)) OR (ai-ji)) OR (Tai Chi)) OR (Chi, Tai)) OR (Tai Chi Chuan)) OR (Taiji)) OR

(Taijiquan)) OR (T'ai Chi)) OR (Tai Ji Quan)) OR (Ji Quan, Tai)) OR (Quan, Tai Ji)) OR (Yoga)) OR (Qigong)) OR (Ch'i Kung)) OR (Qi Gong))

Embase

#24

#10 AND #23

2,556

#23

#13 OR #16 OR #19 OR #22

54,863

#22

#20 OR #21

3,076

#21

'chi kung' OR **'chigung'** OR **'qi gong'** OR **'qigong'**

3,076

#20

'qigong'/exp

1,787

#19

#17 OR #18

20,025

#18

'yogic meditation' OR **'yoga'**

19,892

#17

'yoga'/exp

16,091

#16

#14 OR #15

6,230

#15

'tai chi chuan' OR **'tai ji'** OR **'taiji quan'** OR **'taijiquan'** OR **'tai chi'**

6,230

#14

'tai chi'/exp

5,339

#13

#11 OR #12

31,594

#12

'mindfulness'

31,594

#11

'mindfulness'/exp

26,556

#10

#3 OR #6 OR #9

1,117,936

#9

#7 OR #8

379,445

#8

'alzeimer disease' OR **'alzeimer`s disease'** OR **'alzeimers disease'** OR **'alzheimer**

dementia' OR **'alzheimer fibrillary change'** OR **'alzheimer fibrillary lesion'** OR **'alzheimer neurofibrillary change'** OR **'alzheimer neurofibrillary degeneration'** OR **'alzheimer neuron degeneration'** OR **'alzheimer perusini disease'** OR **'alzheimer sclerosis'** OR **'alzheimer syndrome'** OR **'alzheimer`s disease'** OR **'alzheimers disease'** OR **'cortical sclerosis, diffuse'** OR **'dementia, alzheimer'** OR **'diffuse cortical sclerosis'** OR **'late onset alzheimer disease'** OR **'alzheimer disease'**

379,445

#7

'alzheimer disease'/exp

313,750

#6

#4 OR #5

278,043

#5

'idiopathic parkinsonism' OR **'lewy bodies of parkinson disease'** OR **'lewy bodies of parkinson`s disease'** OR **'lewy bodies of parkinsons disease'** OR **'lewy body parkinson disease'** OR **'lewy body parkinson`s disease'** OR **'lewy body parkinsons disease'** OR **'paralysis agitans'** OR **'parkinson dementia complex'** OR **'parkinson`s disease'** OR **'parkinsons disease'** OR **'primary parkinsonism'** OR **'parkinson disease'**

277,637

#4

'parkinson disease'/exp

238,055

#3

#1 OR #2

1,040,543

#2

'neurodegenerative disease' OR **'neurodegenerative diseases'** OR **'degenerative disease'**

207,810

#1

'degenerative disease'/exp

976,374

Cochrane

Search Name: Neurodegenerative Disease

Last Saved: 30/01/2026 15:44:30

Comment:

ID Search

#1 MeSH descriptor: [Neurodegenerative Diseases] explode all trees

#2 (Neurodegenerative Diseases) OR (Degenerative Diseases, Central Nervous System) OR (Degenerative Neurologic Disorders) OR (Degenerative Conditions, Neurologic) OR (Neurodegenerative Disease)

#3 (Neurodegenerative Disease) OR (Neurologic Degenerative Diseases) OR (Degenerative Neurologic Disorder) OR (Neurodegenerative Disorder) OR (Neurologic Disease, Degenerative)

#4 (Neurologic Disease, Degenerative) OR (Degenerative Diseases, Nervous System) OR (Degenerative Diseases, Neurologic) OR (Neurologic Degenerative Disease) OR (Neurodegenerative Disorders)

#5 (Neurodegenerative Disorders) OR (Nervous System Degenerative Diseases) OR (Degenerative Condition, Neurologic) OR (Neurologic Degenerative Condition) OR (Neurologic Degenerative Conditions)

#6 (Neurologic Degenerative Conditions) OR (Neurologic Disorder, Degenerative) OR (Degenerative Neurologic Diseases) OR (Neurologic Disorders, Degenerative) OR (Neurologic Diseases, Degenerative)

#7 (Neurologic Diseases, Degenerative) OR (Degenerative Neurologic Disease) OR (Degenerative Diseases, Spinal Cord) OR (Parkinson Disease) OR (Lewy Body Parkinson's Disease)

#8 (Lewy Body Parkinson's Disease) OR (Idiopathic Parkinson's Disease) OR (Parkinson Disease, Idiopathic) OR (Parkinson's Disease) OR (Primary Parkinsonism)

#9 (Primary Parkinsonism) OR (Parkinson's Disease, Lewy Body) OR (Paralysis Agitans) OR (Parkinson's Disease, Idiopathic) OR (Lewy Body Parkinson Disease)

#10 (Lewy Body Parkinson Disease) OR (Idiopathic Parkinson Disease) OR (Parkinsonism, Primary) OR (Alzheimer Disease) OR (Familial Alzheimer Diseases (FAD))

#11 (Familial Alzheimer Diseases (FAD)) OR (Alzheimer Disease, Familial (FAD)) OR (Familial Alzheimer Disease (FAD)) OR (Alzheimer's Disease, Focal Onset) OR (Focal Onset Alzheimer's Disease)

#12 (Focal Onset Alzheimer's Disease) OR (Acute Confusional Senile Dementia) OR (Senile Dementia, Acute Confusional) OR (Alzheimer Disease, Late Onset) OR (Late Onset Alzheimer Disease)

#13 (Late Onset Alzheimer Disease) OR (Early Onset Alzheimer Disease) OR (Alzheimer Disease, Early Onset) OR (Presenile Alzheimer Dementia) OR (Dementia, Presenile)

#14 (Dementia, Presenile) OR (Presenile Dementia) OR (Alzheimer's Diseases) OR (Alzheimers Diseases) OR (Alzheimer Type Dementia (ATD))

#15 (Alzheimer Type Dementia (ATD)) OR (Alzheimer Dementia) OR (Alzheimer Type Dementia) OR (Senile Dementia, Alzheimer Type) OR (Alzheimer Type Senile Dementia)

#16 (Alzheimer Type Senile Dementia) OR (Senile Dementia) OR (Dementia, Senile) OR (Dementia, Primary Senile Degenerative) OR (Dementia, Alzheimer Type)

#17 (Dementia, Alzheimer Type) OR (Dementia, Alzheimer) OR (Alzheimer Diseases) OR (Alzheimer-Type Dementia (ATD)) OR (Alzheimer's Disease)

#18 (Alzheimer's Disease) OR (Dementia, Alzheimer-Type (ATD)) OR (Alzheimer Dementias) OR (Primary Senile Degenerative Dementia) OR (Sclerosis, Alzheimer)

#19 (Sclerosis, Alzheimer) OR (Alzheimer Sclerosis) OR (Alzheimer Syndrome)

#20 #1 OR #2 OR #3 OR #4 OR #5 OR #6 OR #7 OR #8 OR #9 OR #10 #11 OR #12 OR #13 OR #14 OR #15 OR #16 OR #17 OR #18 OR #19

#21 MeSH descriptor: [Mindfulness] explode all trees

#22 (Mindfulness) OR (Mindfulness Meditation) OR (Meditation, Mindfulness) OR (Mindfulness Meditations) OR (Tai Ji)

#23 (Tai Ji) OR (Tai Chi Chuan) OR (Tai-ji) OR (Taijiquan) OR (T'ai Chi)

#24 (T'ai Chi) OR (Quan, Tai Ji) OR (Tai Ji Quan) OR (Chi, Tai) OR (Tai Chi)

#25 (Tai Chi) OR (Ji Quan, Tai) OR (Taiji) OR (Yoga) OR (Qigong)

#26 (Qigong) OR (Ch'i Kung) OR (Qi Gong)

#27 #21 OR #22 OR #23 OR #24 OR #25 OR #26

#28 #20 AND #27

Web of Science

TS=(Neurodegenerative Diseases OR Neurodegenerative Disease OR Degenerative Neurologic Disorders OR Degenerative Neurologic Disorder OR Nervous System Degenerative Diseases OR Neurodegenerative Disorders OR Neurodegenerative Disorder OR Neurologic Degenerative Disease OR Neurologic Degenerative Conditions OR Neurologic Degenerative Condition OR Neurologic Degenerative Diseases OR Degenerative Neurologic Diseases OR Degenerative Neurologic Disease OR Parkinson Disease OR Idiopathic Parkinson Disease OR Idiopathic Parkinson's Disease OR Lewy Body Parkinson Disease OR Lewy Body Parkinson's Disease OR Paralysis Agitans OR Parkinson's Disease OR Primary Parkinsonism OR Alzheimer Syndrome OR Alzheimer-Type Dementia (ATD) OR Alzheimer Type Dementia (ATD) OR Alzheimer's Diseases OR Alzheimer Diseases OR Alzheimers Diseases OR Alzheimer DementiaOR Alzheimer Dementias OR Alzheimer's Disease OR Senile Dementia OR Alzheimer Type Dementia OR Alzheimer Type Senile Dementia OR Primary Senile Degenerative Dementia OR Alzheimer Sclerosis OR Presenile Dementia OR Acute Confusional Senile Dementia OR Early Onset Alzheimer Disease OR Presenile Alzheimer Dementia OR Late Onset Alzheimer Disease OR Focal Onset Alzheimer's Disease OR Familial Alzheimer Disease (FAD) OR Familial Alzheimer Diseases (FAD))

TS=(Mindfulness OR Mindfulness Meditation OR Meditation, Mindfulness OR Mindfulness Meditations OR Tai Ji OR ai-ji OR Tai Chi OR Chi, Tai OR Tai Chi Chuan OR Taiji OR Taijiquan OR T'ai Chi OR Tai Ji Quan OR Ji Quan, Tai OR Quan, Tai Ji OR Yoga OR Qigong OR Ch'i Kung OR Qi Gong)
#1 AND #2

eTable 2 Detailed characteristics and eligibility assessment

References	Disease Name	Diagnostic Criteria	Stage of disease	Hoehn&Yahr (stage Ⅰ.Ⅱ.Ⅲ)	Baseline cognitive impairment (MoCA/MMSE)	Baseline movement disorder (UPDRS-III/TUG)	Does it fit PICOS-P?	Intervention Classification
Adrienne G et al.	High-risk group fo AD	SCD+CVRFs	High-risk	NR	NR	NR	√	Category A
Beatrix KS et al.	High-risk group for AD	SCD+CVRFs	High-risk	NR	NR	NR	√	Category A
Choi et al.	PD	NR	H&Y I–III	C:1.8±0.3<br>E:1.6±0.6	NR	TUG<br>C:8.06±2.91<br>E:7.39±0.89	√	Category A
Domingo J et al.	AD	NINCDS-ARDA criteria	Mild to moderate	NR	MMSE<br>C:21.60±2.42<br>E:22.46±2.8	NR	√	Category B
Gao et al.	PD	NR	H&Y I–III	C:6/12/21<br>E:1/19/17	NR	UPDRS III<br>C:30.62±9.90<br>E:31.86±11.49<br>TUG<br>C:11.58±2.83<br>E:10.89±3.17	√	Category A
Gloria VD et al.	PD	NR	H&Y I–III	2/2.5<br>C:13/3<br>E:11/5	NR	UPDRS-III<br>C:20.63±8.69<br>E:26.38±9.64	√	Category A

References	Disease Name	Diagnostic Criteria	Stage of disease	Hoehn&Yahr (stage Ⅰ.Ⅱ.Ⅲ)	Baseline cognitive impairment (MoCA/MMSE)	Baseline movement disorder (UPDRS-III/TUG)	Does it fit PICOS-P?	Intervention Classification
						TUG<br>C:9.72±1.88<br>E:9.69±2.71		
Huang et al.	AD	DSM-IV diagnostic criteria	Mild to moderate	NR	MoCA<br>C:13.32±4.56<br>E:13.06±5.34<br>MMSE<br>C:20.79±5.16<br>E:20.72±6.56	NR	√	Category A
Joe RN et al.	PD	UKPDSBB criteria	H&Y I–III	C:2–3<br>E:2–3	NR	NR	√	Category A
Kwok et al.	PD	NR	H&Y I–III	C:2/19/46<br>E:0/23/48	NR	UPDRS III<br>C:31.64±15.59<br>E:34.90±14.88<br>TUG<br>C:14.05±6.04<br>E:17.54±15.95	√	Category A
Kwok et al.	PD	NR	H&Y I–III	C:0/18/36<br>E:0/18/34	NR	UPDRS-III<br>C:36.91±6.17<br>E:37.40±6.14	√	Category A

References	Disease Name	Diagnostic Criteria	Stage of disease	Hoehn&Yahr (stage Ⅰ.Ⅱ.Ⅲ)	Baseline cognitive impairment (MoCA/MMSE)	Baseline movement disorder (UPDRS-III/TUG)	Does it fit PICOS-P?	Intervention Classification
Lee et al.	PD	NR	H&Y I–III	C:7/5/4<br>E:7/10/8	NR	UPDRS-III<br>C:34.3±7.2<br>E:38.9±13.5	√	Category A
Li et al.	PD	NR	H&Y I–III	C:8/28/29<br>E:9/34/22	NR	UPDRS-III<br>C:15.06±6.17<br>E:15.28±5.59<br>TUG<br>C:8.69±3.18<br>E:8.60±2.90	√	Category A
Li et al. a	PD	UKPDSBB criteria	H&Y I–III	C:7/9/4<br>E:10/7/3	NR	UPDRS-III<br>C:24.15±15.89<br>E:21.63±18.36<br>TUG<br>C:11.24±3.90<br>E:11.43±3.48	√	Category A
Li et al. b	PD	UKPDSBB criteria	H&Y I–III	NR	NR	UPDRS-III<br>C:14.88±2.42<br>E:14.67±1.63<br>TUG<br>C:13.78±7.29<br>E:11.49±2.26	√	Category A

References	Disease Name	Diagnostic Criteria	Stage of disease	Hoehn&Yahr (stage Ⅰ.Ⅱ.Ⅲ)	Baseline cognitive impairment (MoCA/MMSE)	Baseline movement disorder (UPDRS-III/TUG)	Does it fit PICOS-P?	Intervention Classification
Li et al. c	PD	UKPDSBB criteria and MDS clinical diagnostic criteria	H&Y I–III	1.0/1.5/2.0/2.5<br>C:1/11/13/7<br>E:9/5/13/5	NR	UPDRS-III<br>C:19.30±4.87<br>E:25.20±12.50<br>TUG<br>C:10.56±1.98<br>E:12.23±2.74	√	Category A
Li et al.	PD	MDS clinical diagnostic criteria	H&Y I–III	C:2.56±0.56<br>E:2.57±0.57	NR	UPDRS-III<br>C:38.52±16.023<br>E:40.67±16.964	√	Category A
Lin et al.	AD	NINDS-ADRDA criteria	Mild to moderate	NR	MMSE<br>C:21.20±4.19<br>E:17.64±7.42	NR	√	Category A
Lin et al.	PD	NR	H&Y I–III	C:1.60±0.57<br>E:1.50±0.97	MoCA<br>C:24.47±3.48<br>E:24.27±2.99<br>MMSE<br>C:28.07±1.91<br>E:26.93±2.05	UPDRS-III<br>C:16.73±13.40<br>E:15.40±10.21	√	Category A
Liu et al.	PD	NR	Mild to moderate	NR	NR	TUG<br>C:9.19±2.97<br>E:11.19±2.78	√	Category A

References	Disease Name	Diagnostic Criteria	Stage of disease	Hoehn&Yahr (stage Ⅰ.Ⅱ.Ⅲ)	Baseline cognitive impairment (MoCA/MMSE)	Baseline movement disorder (UPDRS-III/TUG)	Does it fit PICOS-P?	Intervention Classification
Ni et al.	PD	NR	H&Y I–III	C:2.1±0.7<br>E:2.2±0.7	NR	UPDRS-III<br>C:27.6±7.8<br>E:28.15±11<br>TUG<br>C:10.2±2.4<br>E:10.27±3.9	√	Category A
Son et al.	PD	NR	H&Y I–III	C:0/30/0<br>E:2/29/2	MoCA<br>C:21.48±5.13<br>E:22.87±2.54	NR	√	Category C
Thunga AV et al.	PD	Queen Square Brain Bank	H&Y I–III	C:2/5/5<br>E:2/2/8	NR	UPDRS-III<br>C:19.08±6.07<br>E:15.25±3.77	√	Category A
Timo MB et al.	PD	MDS clinical diagnostic criteria	H&Y I–III	C:2.00±0.74<br>E:2.00±1.48	NR	UPDRS-III<br>C:31±20.74<br>E:32±30.37	√	Category B
Wan et al.	PD	NR	H&Y I–IV	NR	NR	TUG<br>C:11.77±5.52<br>E:11.47±4.67	√	Category A
Wang et al.	PD	MDS clinical diagnostic	H&Y I–III	C:1.23±0.40<br>E:1.28±0.45	MoCA<br>C:27.5±1.77	UPDRS-III<br>C:21.18±12.89	√	Category A

**References**	**Disease Name**	**Diagnostic Criteria**	**Stage of disease**	**Hoehn&Yahr (stage Ⅰ.Ⅱ.Ⅲ)**	**Baseline cognitive impairment (MoCA/MMSE)**	**Baseline movement disorder (UPDRS-III/TUG)**	**Does it fit PICOS-P?**	**Intervention Classification**
		criteria			E:26.70±1.55	E:20.78±7.57<br>TUG<br>C:12.59±5.58<br>E:9.96±2.17		
Witoon M et al.	PD	UKPDSBB criteria	H&Y I–III	C:4/5/7<br>E:4/10/3	NR	UPDRS-III<br>C:36.1±18.3<br>E:29.5±14.5<br>TUG<br>C:13.9±4.5<br>E:12.6±3.0	√	Category A
Xiao et al.	PD	NR	H&Y I–III	C:2.1±0.23<br>E:2.2±0.21	NR	UPDRS-III<br>C:26.9±2.05<br>E:27.4±2.51<br>TUG<br>C:12.1±1.69<br>E:12.4±1.77	√	Category A
Yin et al.	PD	Chinese PD Criteria 2016	H&Y I–III	C:2±0.37<br>E:2±0.74	NR	UPDRS-III<br>C:22.65±11.91<br>E:20.65±12.98	√	Category A

Sensitivity Analysis

eFigure 1. shows the sensitivity analysis of UPDRS-III

Study or Subgroup	Experimental Mean	Experimental SD	Experimental Total	Control Mean	Control SD	Control Total	Weight	Mean Difference IV, Random, 95% CI
Gao et al. 2014	-8.05	10.91	37	-1.9	11.25	39	0.0%	-6.15 [-11.13, -1.17]
Gloria VD et al. 2018	3.04	9.23	12	5.58	8.38	13	4.8%	-2.54 [-9.47, 4.39]
Kwok et al. 2019	-13.8	14.29	58	-9.11	15.15	59	6.1%	-4.69 [-10.03, 0.65]
Kwok et al. 2025	-8.01	6.33	52	-0.93	6.62	54	8.6%	-7.08 [-9.55, -4.61]
Lee et al. 2018	-1	6.8	25	0.9	3.77	16	8.0%	-1.90 [-5.14, 1.34]
Li et al. 2012	-6.42	5.02	65	-1.4	6.96	65	8.9%	-5.02 [-7.11, -2.93]
Li et al. 2022a	-8.68	15.9	19	-2.6	16.2	19	3.0%	-6.08 [-16.29, 4.13]
Li et al. 2022b	-2.2	1.7	15	0.25	3.34	16	9.1%	-2.45 [-4.30, -0.60]
Li et al. 2022c	-6.1	11.32	32	11.4	6.48	17	6.4%	-17.50 [-22.49, -12.51]
Li et al. 2024	-4.19	16.61	27	-0.22	16.13	27	3.7%	-3.97 [-12.70, 4.76]
Lin et al. 2023	-5	5.77	15	-3	3.3	15	7.8%	-2.00 [-5.36, 1.36]
Ni et al. 2016	-10.9	5.13	13	0.4	2.52	10	8.0%	-11.30 [-14.50, -8.10]
Thunga AV et al. 2022	-3	4.27	12	-2.66	5.76	12	7.2%	-0.34 [-4.40, 3.72]
Timo MB et al. 2021	3	30.01	14	1	22.82	16	1.1%	2.00 [-17.29, 21.29]
Wang et al. 2022	-7.43	7.45	23	-5.73	11.23	22	5.8%	-1.70 [-7.29, 3.89]
Witoon M et al. 2022	-0.8	13.5	17	2.6	20.28	16	2.5%	-3.40 [-15.23, 8.43]
Xiao et al. 2016	-3.1	20.49	45	1.5	16.19	44	4.3%	-4.60 [-12.26, 3.06]
Yin et al. 2025	-0.5	13.8	25	-2.35	12.28	26	4.6%	1.85 [-5.33, 9.03]
**Total (95% CI)**			**469**			**447**	**100.0%**	**-4.64 [-6.79, -2.50]**

Heterogeneity: Tau² = 12.27; Chi² = 66.07, df = 16 (P < 0.00001); I² = 76%
Test for overall effect: Z = 4.25 (P < 0.0001)

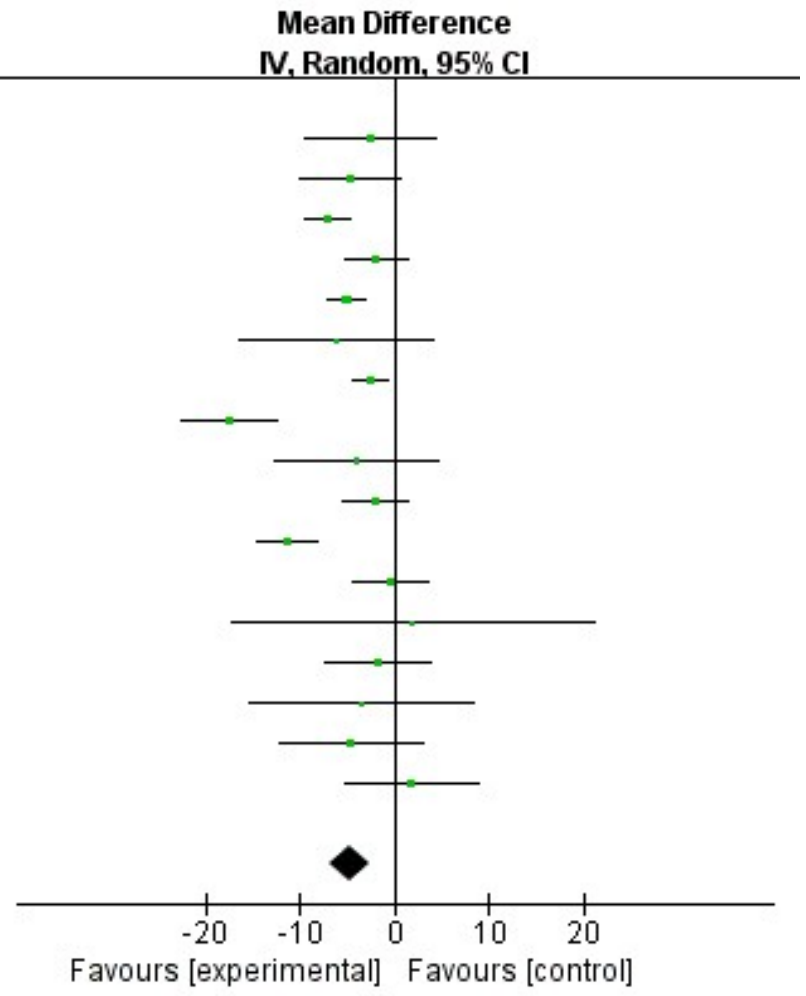

Study or Subgroup	Experimental Mean	Experimental SD	Experimental Total	Control Mean	Control SD	Control Total	Weight	Mean Difference IV, Random, 95% CI
Gao et al. 2014	-8.05	10.91	37	-1.9	11.25	39	6.3%	-6.15 [-11.13, -1.17]
Gloria VD et al. 2018	3.04	9.23	12	5.58	8.38	13	0.0%	-2.54 [-9.47, 4.39]
Kwok et al. 2019	-13.8	14.29	58	-9.11	15.15	59	6.0%	-4.69 [-10.03, 0.65]
Kwok et al. 2025	-8.01	6.33	52	-0.93	6.62	54	8.5%	-7.08 [-9.55, -4.61]
Lee et al. 2018	-1	6.8	25	0.9	3.77	16	7.9%	-1.90 [-5.14, 1.34]
Li et al. 2012	-6.42	5.02	65	-1.4	6.96	65	8.8%	-5.02 [-7.11, -2.93]
Li et al. 2022a	-8.68	15.9	19	-2.6	16.2	19	3.0%	-6.08 [-16.29, 4.13]
Li et al. 2022b	-2.2	1.7	15	0.25	3.34	16	9.0%	-2.45 [-4.30, -0.60]
Li et al. 2022c	-6.1	11.32	32	11.4	6.48	17	6.3%	-17.50 [-22.49, -12.51]
Li et al. 2024	-4.19	16.61	27	-0.22	16.13	27	3.6%	-3.97 [-12.70, 4.76]
Lin et al. 2023	-5	5.77	15	-3	3.3	15	7.7%	-2.00 [-5.36, 1.36]
Ni et al. 2016	-10.9	5.13	13	0.4	2.52	10	7.9%	-11.30 [-14.50, -8.10]
Thunga AV et al. 2022	-3	4.27	12	-2.66	5.76	12	7.1%	-0.34 [-4.40, 3.72]
Timo MB et al. 2021	3	30.01	14	1	22.82	16	1.1%	2.00 [-17.29, 21.29]
Wang et al. 2022	-7.43	7.45	23	-5.73	11.23	22	5.7%	-1.70 [-7.29, 3.89]
Witoon M et al. 2022	-0.8	13.5	17	2.6	20.28	16	2.4%	-3.40 [-15.23, 8.43]
Xiao et al. 2016	-3.1	20.49	45	1.5	16.19	44	4.2%	-4.60 [-12.26, 3.06]
Yin et al. 2025	-0.5	13.8	25	-2.35	12.28	26	4.6%	1.85 [-5.33, 9.03]
**Total (95% CI)**			**494**			**473**	**100.0%**	**-4.84 [-6.95, -2.73]**

Heterogeneity: Tau² = 12.03; Chi² = 66.06, df = 16 (P < 0.00001); I² = 76%
Test for overall effect: Z = 4.50 (P < 0.00001)

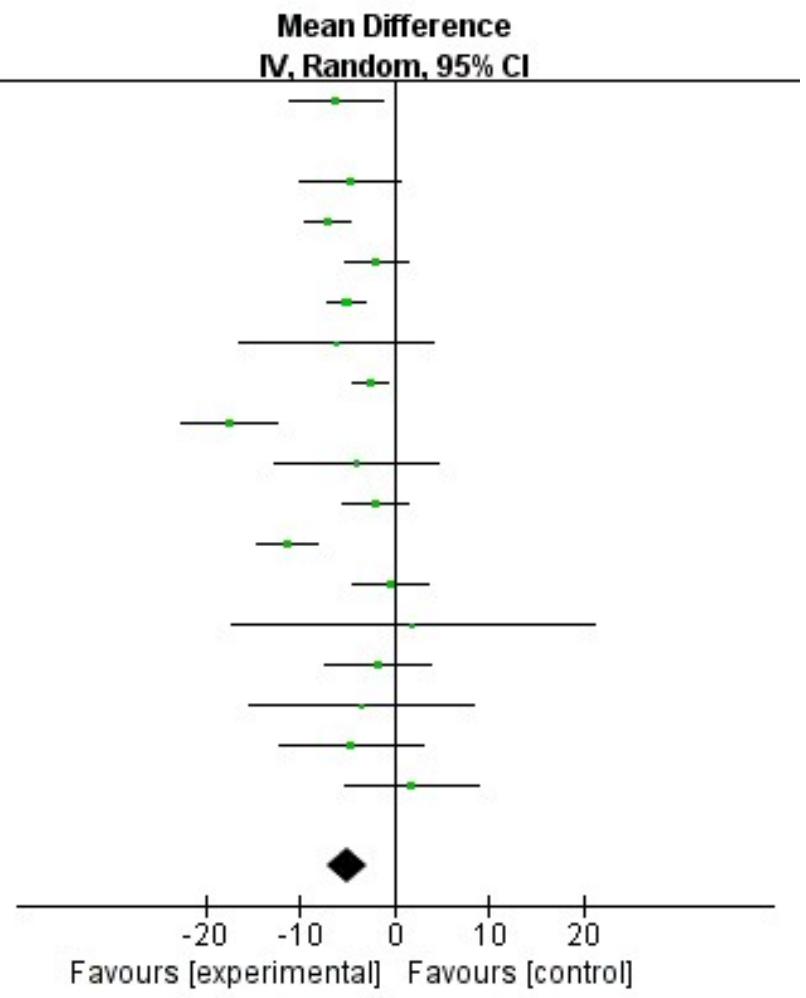

Study or Subgroup	Experimental Mean	Experimental SD	Experimental Total	Control Mean	Control SD	Control Total	Weight	Mean Difference IV, Random, 95% CI
Gao et al. 2014	-8.05	10.91	37	-1.9	11.25	39	6.4%	-6.15 [-11.13, -1.17]
Gloria VD et al. 2018	3.04	9.23	12	5.58	8.38	13	4.8%	-2.54 [-9.47, 4.39]
Kwok et al. 2019	-13.8	14.29	58	-9.11	15.15	59	0.0%	-4.69 [-10.03, 0.65]
Kwok et al. 2025	-8.01	6.33	52	-0.93	6.62	54	8.6%	-7.08 [-9.55, -4.61]
Lee et al. 2018	-1	6.8	25	0.9	3.77	16	7.9%	-1.90 [-5.14, 1.34]
Li et al. 2012	-6.42	5.02	65	-1.4	6.96	65	8.9%	-5.02 [-7.11, -2.93]
Li et al. 2022a	-8.68	15.9	19	-2.6	16.2	19	3.0%	-6.08 [-16.29, 4.13]
Li et al. 2022b	-2.2	1.7	15	0.25	3.34	16	9.0%	-2.45 [-4.30, -0.60]
Li et al. 2022c	-6.1	11.32	32	11.4	6.48	17	6.4%	-17.50 [-22.49, -12.51]
Li et al. 2024	-4.19	16.61	27	-0.22	16.13	27	3.7%	-3.97 [-12.70, 4.76]
Lin et al. 2023	-5	5.77	15	-3	3.3	15	7.8%	-2.00 [-5.36, 1.36]
Ni et al. 2016	-10.9	5.13	13	0.4	2.52	10	8.0%	-11.30 [-14.50, -8.10]
Thunga AV et al. 2022	-3	4.27	12	-2.66	5.76	12	7.2%	-0.34 [-4.40, 3.72]
Timo MB et al. 2021	3	30.01	14	1	22.82	16	1.1%	2.00 [-17.29, 21.29]
Wang et al. 2022	-7.43	7.45	23	-5.73	11.23	22	5.8%	-1.70 [-7.29, 3.89]
Witoon M et al. 2022	-0.8	13.5	17	2.6	20.28	16	2.4%	-3.40 [-15.23, 8.43]
Xiao et al. 2016	-3.1	20.49	45	1.5	16.19	44	4.3%	-4.60 [-12.26, 3.06]
Yin et al. 2025	-0.5	13.8	25	-2.35	12.28	26	4.6%	1.85 [-5.33, 9.03]
**Total (95% CI)**			**448**			**427**	**100.0%**	**-4.74 [-6.88, -2.60]**

Heterogeneity: Tau² = 12.29; Chi² = 66.42, df = 16 (P < 0.00001); I² = 76%
Test for overall effect: Z = 4.34 (P < 0.0001)

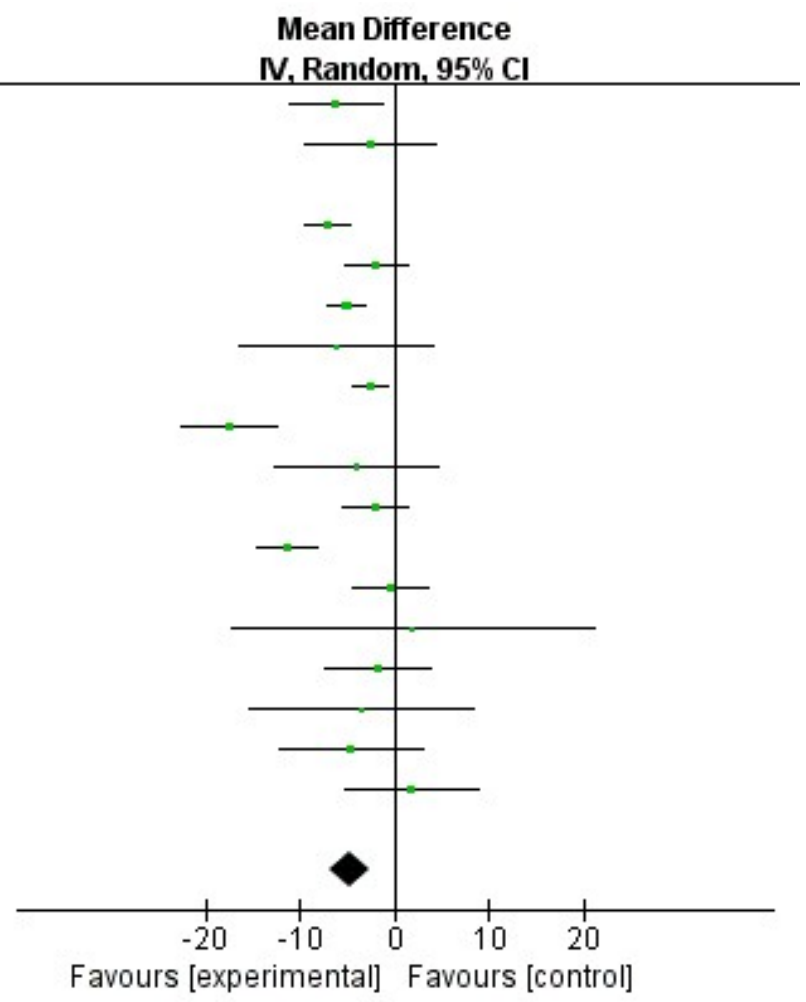

Study or Subgroup	Experimental Mean	Experimental SD	Experimental Total	Control Mean	Control SD	Control Total	Weight	Mean Difference IV, Random, 95% CI
Gao et al. 2014	-8.05	10.91	37	-1.9	11.25	39	6.5%	-6.15 [-11.13, -1.17]
Gloria VD et al. 2018	3.04	9.23	12	5.58	8.38	13	5.0%	-2.54 [-9.47, 4.39]
Kwok et al. 2019	-13.8	14.29	58	-9.11	15.15	59	6.2%	-4.69 [-10.03, 0.65]
Kwok et al. 2025	-8.01	6.33	52	-0.93	6.62	54	0.0%	-7.08 [-9.55, -4.61]
Lee et al. 2018	-1	6.8	25	0.9	3.77	16	8.1%	-1.90 [-5.14, 1.34]
Li et al. 2012	-6.42	5.02	65	-1.4	6.96	65	9.0%	-5.02 [-7.11, -2.93]
Li et al. 2022a	-8.68	15.9	19	-2.6	16.2	19	3.1%	-6.08 [-16.29, 4.13]
Li et al. 2022b	-2.2	1.7	15	0.25	3.34	16	9.2%	-2.45 [-4.30, -0.60]
Li et al. 2022c	-6.1	11.32	32	11.4	6.48	17	6.5%	-17.50 [-22.49, -12.51]
Li et al. 2024	-4.19	16.61	27	-0.22	16.13	27	3.8%	-3.97 [-12.70, 4.76]
Lin et al. 2023	-5	5.77	15	-3	3.3	15	8.0%	-2.00 [-5.36, 1.36]
Ni et al. 2016	-10.9	5.13	13	0.4	2.52	10	8.1%	-11.30 [-14.50, -8.10]
Thunga AV et al. 2022	-3	4.27	12	-2.66	5.76	12	7.4%	-0.34 [-4.40, 3.72]
Timo MB et al. 2021	3	30.01	14	1	22.82	16	1.1%	2.00 [-17.29, 21.29]
Wang et al. 2022	-7.43	7.45	23	-5.73	11.23	22	6.0%	-1.70 [-7.29, 3.89]
Witoon M et al. 2022	-0.8	13.5	17	2.6	20.28	16	2.6%	-3.40 [-15.23, 8.43]
Xiao et al. 2016	-3.1	20.49	45	1.5	16.19	44	4.5%	-4.60 [-12.26, 3.06]
Yin et al. 2025	-0.5	13.8	25	-2.35	12.28	26	4.8%	1.85 [-5.33, 9.03]
**Total (95% CI)**			**454**			**432**	**100.0%**	**-4.52 [-6.72, -2.33]**

Heterogeneity: Tau² = 12.76; Chi² = 62.21, df = 16 (P < 0.00001); I² = 74%
Test for overall effect: Z = 4.04 (P < 0.0001)

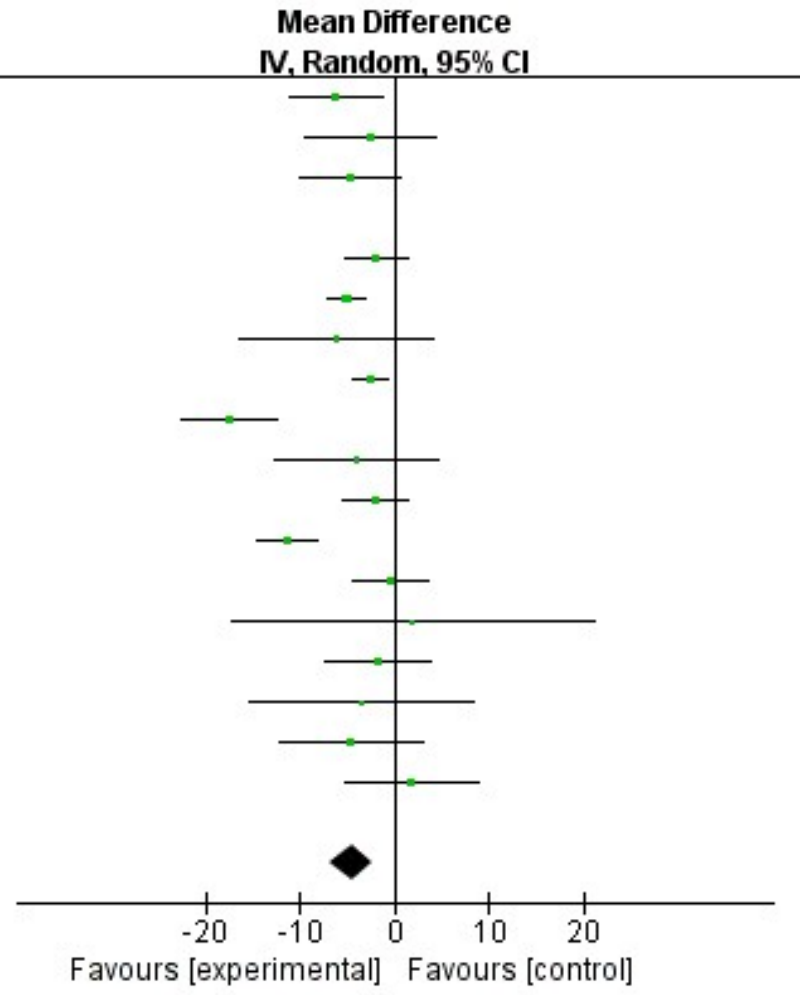

Study or Subgroup	Experimental Mean	Experimental SD	Experimental Total	Control Mean	Control SD	Control Total	Weight	Mean Difference IV, Random, 95% CI
Gao et al. 2014	-8.05	10.91	37	-1.9	11.25	39	6.5%	-6.15 [-11.13, -1.17]
Gloria VD et al. 2018	3.04	9.23	12	5.58	8.38	13	4.9%	-2.54 [-9.47, 4.39]
Kwok et al. 2019	-13.8	14.29	58	-9.11	15.15	59	6.2%	-4.69 [-10.03, 0.65]
Kwok et al. 2025	-8.01	6.33	52	-0.93	6.62	54	8.8%	-7.08 [-9.55, -4.61]
Lee et al. 2018	-1	6.8	25	0.9	3.77	16	0.0%	-1.90 [-5.14, 1.34]
Li et al. 2012	-6.42	5.02	65	-1.4	6.96	65	9.1%	-5.02 [-7.11, -2.93]
Li et al. 2022a	-8.68	15.9	19	-2.6	16.2	19	3.1%	-6.08 [-16.29, 4.13]
Li et al. 2022b	-2.2	1.7	15	0.25	3.34	16	9.2%	-2.45 [-4.30, -0.60]
Li et al. 2022c	-6.1	11.32	32	11.4	6.48	17	6.5%	-17.50 [-22.49, -12.51]
Li et al. 2024	-4.19	16.61	27	-0.22	16.13	27	3.8%	-3.97 [-12.70, 4.76]
Lin et al. 2023	-5	5.77	15	-3	3.3	15	8.0%	-2.00 [-5.36, 1.36]
Ni et al. 2016	-10.9	5.13	13	0.4	2.52	10	8.1%	-11.30 [-14.50, -8.10]
Thunga AV et al. 2022	-3	4.27	12	-2.66	5.76	12	7.3%	-0.34 [-4.40, 3.72]
Timo MB et al. 2021	3	30.01	14	1	22.82	16	1.1%	2.00 [-17.29, 21.29]
Wang et al. 2022	-7.43	7.45	23	-5.73	11.23	22	5.9%	-1.70 [-7.29, 3.89]
Witoon M et al. 2022	-0.8	13.5	17	2.6	20.28	16	2.5%	-3.40 [-15.23, 8.43]
Xiao et al. 2016	-3.1	20.49	45	1.5	16.19	44	4.4%	-4.60 [-12.26, 3.06]
Yin et al. 2025	-0.5	13.8	25	-2.35	12.28	26	4.7%	1.85 [-5.33, 9.03]
**Total (95% CI)**			**481**			**470**	**100.0%**	**-4.96 [-7.12, -2.81]**

Heterogeneity: Tau² = 12.26; Chi² = 63.39, df = 16 (P < 0.00001); I² = 75%
Test for overall effect: Z = 4.51 (P < 0.00001)

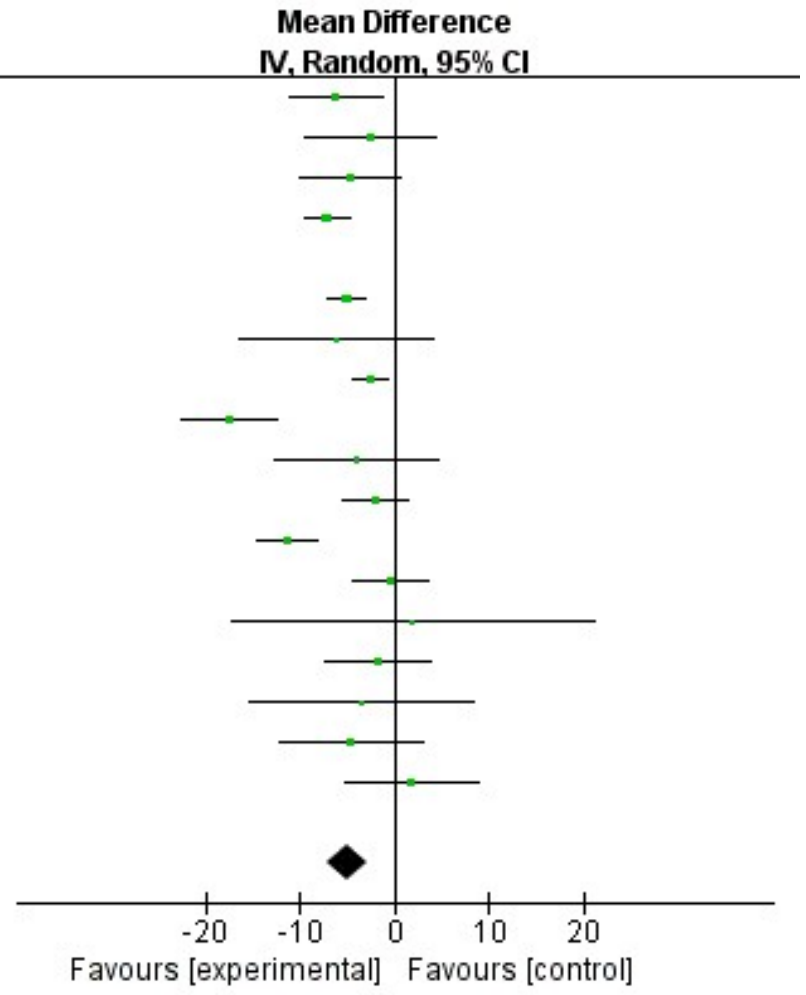

Study or Subgroup	Experimental Mean	Experimental SD	Experimental Total	Control Mean	Control SD	Control Total	Weight	Mean Difference IV, Random, 95% CI
Gao et al. 2014	-8.05	10.91	37	-1.9	11.25	39	6.6%	-6.15 [-11.13, -1.17]
Gloria VD et al. 2018	3.04	9.23	12	5.58	8.38	13	5.1%	-2.54 [-9.47, 4.39]
Kwok et al. 2019	-13.8	14.29	58	-9.11	15.15	59	6.3%	-4.69 [-10.03, 0.65]
Kwok et al. 2025	-8.01	6.33	52	-0.93	6.62	54	8.5%	-7.08 [-9.55, -4.61]
Lee et al. 2018	-1	6.8	25	0.9	3.77	16	8.0%	-1.90 [-5.14, 1.34]
Li et al. 2012	-6.42	5.02	65	-1.4	6.96	65	0.0%	-5.02 [-7.11, -2.93]
Li et al. 2022a	-8.68	15.9	19	-2.6	16.2	19	3.3%	-6.08 [-16.29, 4.13]
Li et al. 2022b	-2.2	1.7	15	0.25	3.34	16	8.9%	-2.45 [-4.30, -0.60]
Li et al. 2022c	-6.1	11.32	32	11.4	6.48	17	6.6%	-17.50 [-22.49, -12.51]
Li et al. 2024	-4.19	16.61	27	-0.22	16.13	27	4.0%	-3.97 [-12.70, 4.76]
Lin et al. 2023	-5	5.77	15	-3	3.3	15	7.9%	-2.00 [-5.36, 1.36]
Ni et al. 2016	-10.9	5.13	13	0.4	2.52	10	8.0%	-11.30 [-14.50, -8.10]
Thunga AV et al. 2022	-3	4.27	12	-2.66	5.76	12	7.3%	-0.34 [-4.40, 3.72]
Timo MB et al. 2021	3	30.01	14	1	22.82	16	1.2%	2.00 [-17.29, 21.29]
Wang et al. 2022	-7.43	7.45	23	-5.73	11.23	22	6.1%	-1.70 [-7.29, 3.89]
Witoon M et al. 2022	-0.8	13.5	17	2.6	20.28	16	2.7%	-3.40 [-15.23, 8.43]
Xiao et al. 2016	-3.1	20.49	45	1.5	16.19	44	4.6%	-4.60 [-12.26, 3.06]
Yin et al. 2025	-0.5	13.8	25	-2.35	12.28	26	4.9%	1.85 [-5.33, 9.03]
**Total (95% CI)**			**441**			**421**	**100.0%**	**-4.69 [-7.00, -2.38]**

Heterogeneity: Tau² = 14.68; Chi² = 66.29, df = 16 (P < 0.00001); I² = 76%
Test for overall effect: Z = 3.98 (P < 0.0001)

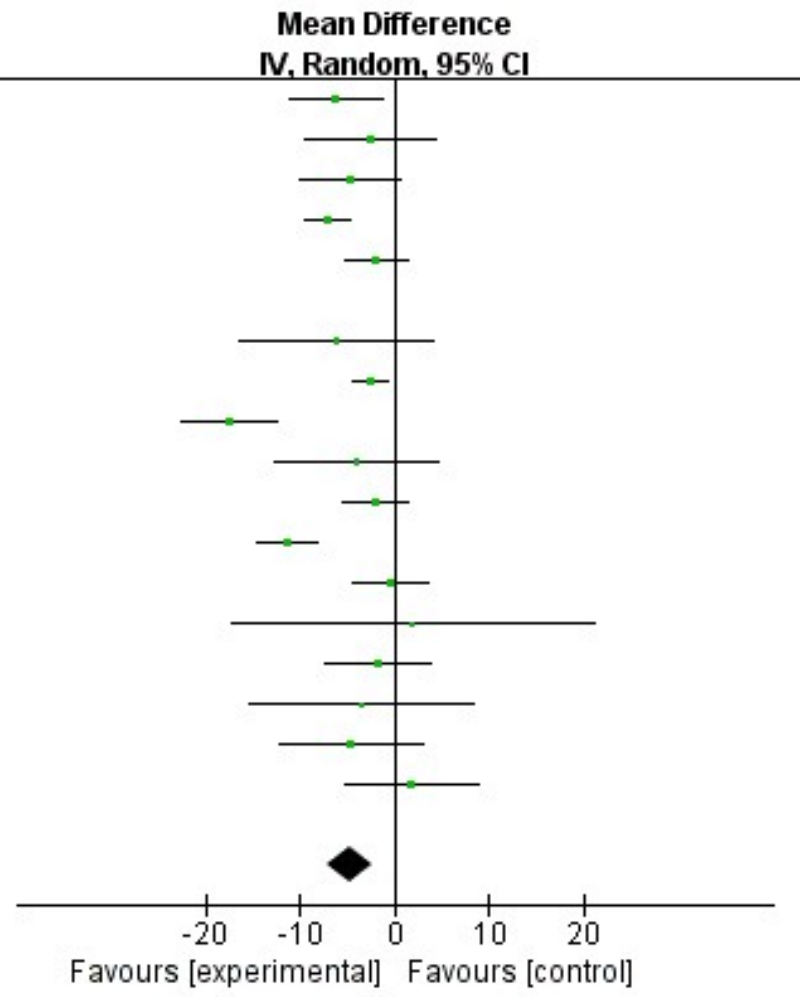

Study or Subgroup	Experimental Mean	Experimental SD	Experimental Total	Control Mean	Control SD	Control Total	Weight	Mean Difference IV, Random, 95% CI
Gao et al. 2014	-8.05	10.91	37	-1.9	11.25	39	6.2%	-6.15 [-11.13, -1.17]
Gloria VD et al. 2018	3.04	9.23	12	5.58	8.38	13	4.6%	-2.54 [-9.47, 4.39]
Kwok et al. 2019	-13.8	14.29	58	-9.11	15.15	59	5.9%	-4.69 [-10.03, 0.65]
Kwok et al. 2025	-8.01	6.33	52	-0.93	6.62	54	8.4%	-7.08 [-9.55, -4.61]
Lee et al. 2018	-1	6.8	25	0.9	3.77	16	7.7%	-1.90 [-5.14, 1.34]
Li et al. 2012	-6.42	5.02	65	-1.4	6.96	65	8.7%	-5.02 [-7.11, -2.93]
Li et al. 2022a	-8.68	15.9	19	-2.6	16.2	19	0.0%	-6.08 [-16.29, 4.13]
Li et al. 2022b	-2.2	1.7	15	0.25	3.34	16	8.8%	-2.45 [-4.30, -0.60]
Li et al. 2022c	-6.1	11.32	32	11.4	6.48	17	6.2%	-17.50 [-22.49, -12.51]
Li et al. 2024	-4.19	16.61	27	-0.22	16.13	27	3.6%	-3.97 [-12.70, 4.76]
Lin et al. 2023	-5	5.77	15	-3	3.3	15	7.6%	-2.00 [-5.36, 1.36]
Ni et al. 2016	-10.9	5.13	13	0.4	2.52	10	7.8%	-11.30 [-14.50, -8.10]
Thunga AV et al. 2022	-3	4.27	12	-2.66	5.76	12	7.0%	-0.34 [-4.40, 3.72]
Timo MB et al. 2021	3	30.01	14	1	22.82	16	1.0%	2.00 [-17.29, 21.29]
Wang et al. 2022	-7.43	7.45	23	-5.73	11.23	22	5.6%	-1.70 [-7.29, 3.89]
Witoon M et al. 2022	-0.8	13.5	17	2.6	20.28	16	2.3%	-3.40 [-15.23, 8.43]
Xiao et al. 2016	-3.1	20.49	45	1.5	16.19	44	4.2%	-4.60 [-12.26, 3.06]
Yin et al. 2025	-0.5	13.8	25	-2.35	12.28	26	4.5%	1.85 [-5.33, 9.03]
**Total (95% CI)**			**487**			**467**	**100.0%**	**-4.70 [-6.79, -2.61]**

Heterogeneity: Tau² = 11.96; Chi² = 66.35, df = 16 (P < 0.00001); I² = 76%
Test for overall effect: Z = 4.41 (P < 0.0001)

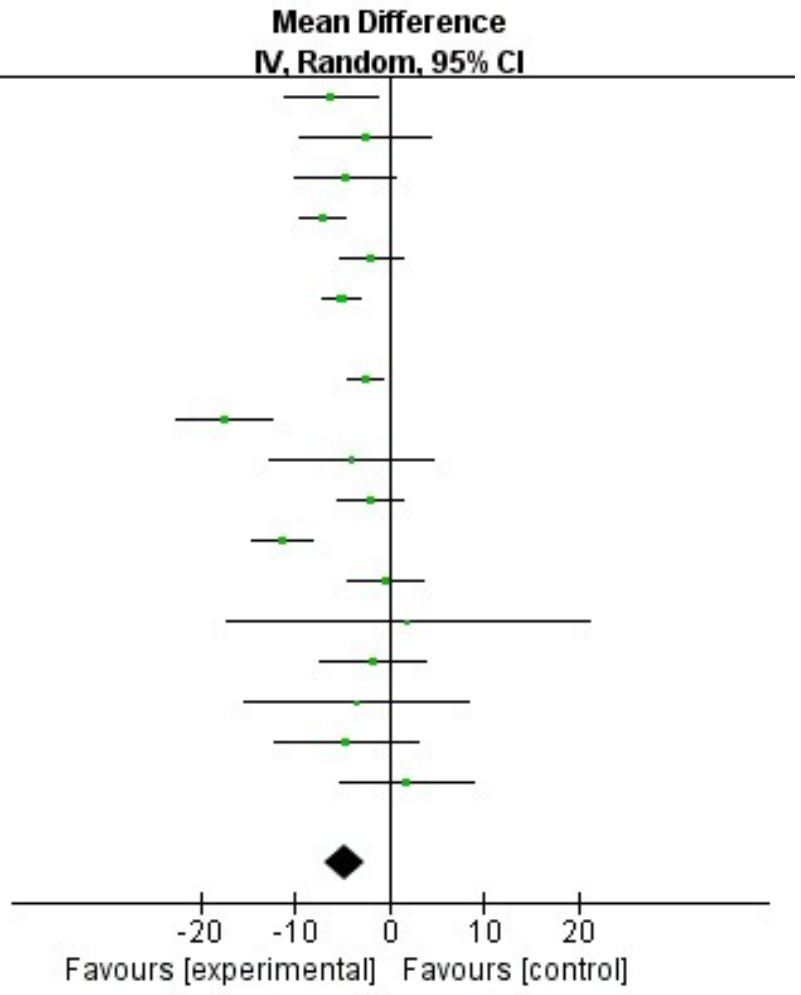

Study or Subgroup	Experimental Mean	Experimental SD	Experimental Total	Control Mean	Control SD	Control Total	Weight	Mean Difference IV, Random, 95% CI
Gao et al. 2014	-8.05	10.91	37	-1.9	11.25	39	6.6%	-6.15 [-11.13, -1.17]
Gloria VD et al. 2018	3.04	9.23	12	5.58	8.38	13	5.0%	-2.54 [-9.47, 4.39]
Kwok et al. 2019	-13.8	14.29	58	-9.11	15.15	59	6.3%	-4.69 [-10.03, 0.65]
Kwok et al. 2025	-8.01	6.33	52	-0.93	6.62	54	8.7%	-7.08 [-9.55, -4.61]
Lee et al. 2018	-1	6.8	25	0.9	3.77	16	8.1%	-1.90 [-5.14, 1.34]
Li et al. 2012	-6.42	5.02	65	-1.4	6.96	65	9.0%	-5.02 [-7.11, -2.93]
Li et al. 2022a	-8.68	15.9	19	-2.6	16.2	19	3.2%	-6.08 [-16.29, 4.13]
Li et al. 2022b	-2.2	1.7	15	0.25	3.34	16	0.0%	-2.45 [-4.30, -0.60]
Li et al. 2022c	-6.1	11.32	32	11.4	6.48	17	6.6%	-17.50 [-22.49, -12.51]
Li et al. 2024	-4.19	16.61	27	-0.22	16.13	27	3.9%	-3.97 [-12.70, 4.76]
Lin et al. 2023	-5	5.77	15	-3	3.3	15	8.0%	-2.00 [-5.36, 1.36]
Ni et al. 2016	-10.9	5.13	13	0.4	2.52	10	8.1%	-11.30 [-14.50, -8.10]
Thunga AV et al. 2022	-3	4.27	12	-2.66	5.76	12	7.4%	-0.34 [-4.40, 3.72]
Timo MB et al. 2021	3	30.01	14	1	22.82	16	1.2%	2.00 [-17.29, 21.29]
Wang et al. 2022	-7.43	7.45	23	-5.73	11.23	22	6.0%	-1.70 [-7.29, 3.89]
Witoon M et al. 2022	-0.8	13.5	17	2.6	20.28	16	2.6%	-3.40 [-15.23, 8.43]
Xiao et al. 2016	-3.1	20.49	45	1.5	16.19	44	4.5%	-4.60 [-12.26, 3.06]
Yin et al. 2025	-0.5	13.8	25	-2.35	12.28	26	4.8%	1.85 [-5.33, 9.03]
**Total (95% CI)**			**491**			**470**	**100.0%**	**-4.93 [-7.16, -2.71]**

Heterogeneity: Tau² = 13.18; Chi² = 59.22, df = 16 (P < 0.00001); I² = 73%
Test for overall effect: Z = 4.35 (P < 0.0001)

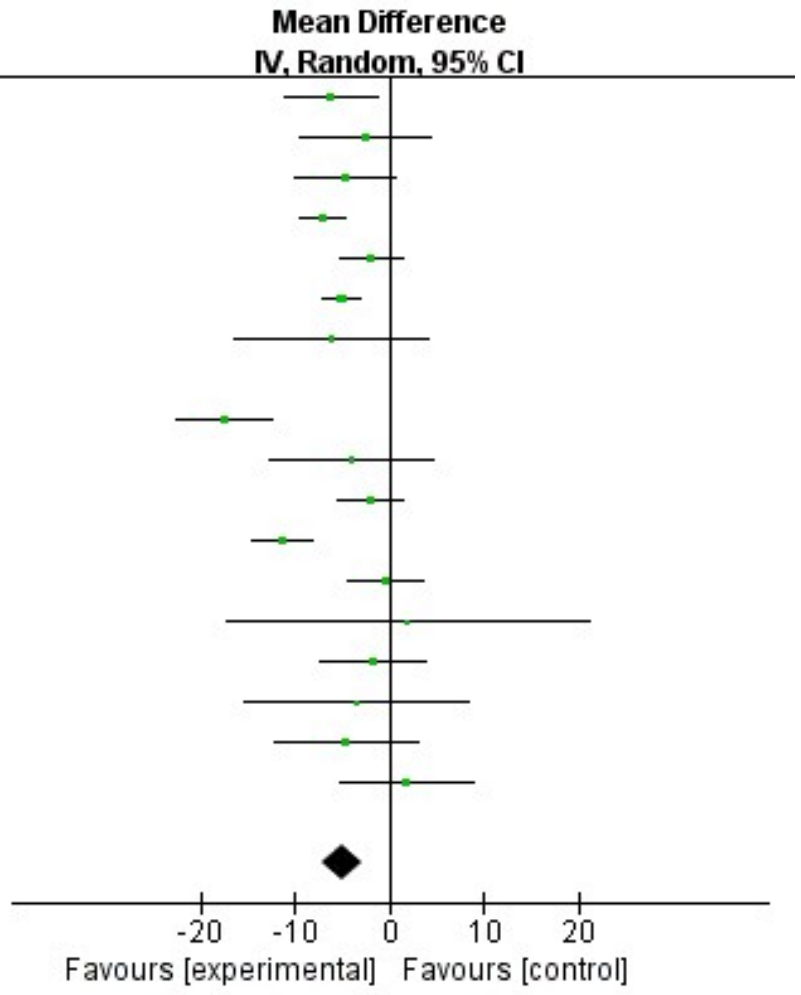

Study or Subgroup	Experimental Mean	Experimental SD	Experimental Total	Control Mean	Control SD	Control Total	Weight	Mean Difference IV, Random, 95% CI
Gao et al. 2014	-8.05	10.91	37	-1.9	11.25	39	6.0%	-6.15 [-11.13, -1.17]
Gloria VD et al. 2018	3.04	9.23	12	5.58	8.38	13	4.0%	-2.54 [-9.47, 4.39]
Kwok et al. 2019	-13.8	14.29	58	-9.11	15.15	59	5.6%	-4.69 [-10.03, 0.65]
Kwok et al. 2025	-8.01	6.33	52	-0.93	6.62	54	9.9%	-7.08 [-9.55, -4.61]
Lee et al. 2018	-1	6.8	25	0.9	3.77	16	8.6%	-1.90 [-5.14, 1.34]
Li et al. 2012	-6.42	5.02	65	-1.4	6.96	65	10.5%	-5.02 [-7.11, -2.93]
Li et al. 2022a	-8.68	15.9	19	-2.6	16.2	19	2.2%	-6.08 [-16.29, 4.13]
Li et al. 2022b	-2.2	1.7	15	0.25	3.34	16	10.9%	-2.45 [-4.30, -0.60]
Li et al. 2022c	-6.1	11.32	32	11.4	6.48	17	0.0%	-17.50 [-22.49, -12.51]
Li et al. 2024	-4.19	16.61	27	-0.22	16.13	27	2.9%	-3.97 [-12.70, 4.76]
Lin et al. 2023	-5	5.77	15	-3	3.3	15	8.4%	-2.00 [-5.36, 1.36]
Ni et al. 2016	-10.9	5.13	13	0.4	2.52	10	8.7%	-11.30 [-14.50, -8.10]
Thunga AV et al. 2022	-3	4.27	12	-2.66	5.76	12	7.3%	-0.34 [-4.40, 3.72]
Timo MB et al. 2021	3	30.01	14	1	22.82	16	0.7%	2.00 [-17.29, 21.29]
Wang et al. 2022	-7.43	7.45	23	-5.73	11.23	22	5.3%	-1.70 [-7.29, 3.89]
Witoon M et al. 2022	-0.8	13.5	17	2.6	20.28	16	1.8%	-3.40 [-15.23, 8.43]
Xiao et al. 2016	-3.1	20.49	45	1.5	16.19	44	3.5%	-4.60 [-12.26, 3.06]
Yin et al. 2025	-0.5	13.8	25	-2.35	12.28	26	3.8%	1.85 [-5.33, 9.03]
**Total (95% CI)**			**474**			**469**	**100.0%**	**-4.04 [-5.73, -2.35]**

Heterogeneity: Tau² = 5.92; Chi² = 40.17, df = 16 (P = 0.0007); I² = 60%
Test for overall effect: Z = 4.68 (P < 0.00001)

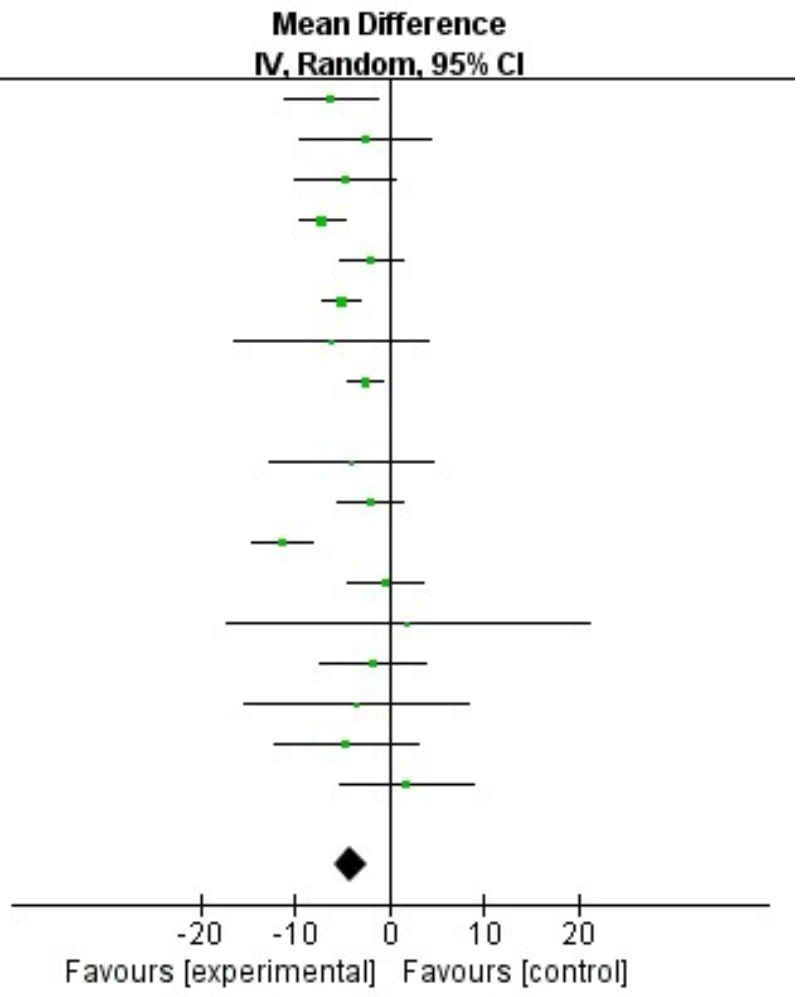

Study or Subgroup	Experimental Mean	Experimental SD	Experimental Total	Control Mean	Control SD	Control Total	Weight	Mean Difference IV, Random, 95% CI
Gao et al. 2014	-8.05	10.91	37	-1.9	11.25	39	6.2%	-6.15 [-11.13, -1.17]
Gloria VD et al. 2018	3.04	9.23	12	5.58	8.38	13	4.7%	-2.54 [-9.47, 4.39]
Kwok et al. 2019	-13.8	14.29	58	-9.11	15.15	59	5.9%	-4.69 [-10.03, 0.65]
Kwok et al. 2025	-8.01	6.33	52	-0.93	6.62	54	8.4%	-7.08 [-9.55, -4.61]
Lee et al. 2018	-1	6.8	25	0.9	3.77	16	7.8%	-1.90 [-5.14, 1.34]
Li et al. 2012	-6.42	5.02	65	-1.4	6.96	65	8.7%	-5.02 [-7.11, -2.93]
Li et al. 2022a	-8.68	15.9	19	-2.6	16.2	19	2.9%	-6.08 [-16.29, 4.13]
Li et al. 2022b	-2.2	1.7	15	0.25	3.34	16	8.9%	-2.45 [-4.30, -0.60]
Li et al. 2022c	-6.1	11.32	32	11.4	6.48	17	6.2%	-17.50 [-22.49, -12.51]
Li et al. 2024	-4.19	16.61	27	-0.22	16.13	27	0.0%	-3.97 [-12.70, 4.76]
Lin et al. 2023	-5	5.77	15	-3	3.3	15	7.7%	-2.00 [-5.36, 1.36]
Ni et al. 2016	-10.9	5.13	13	0.4	2.52	10	7.8%	-11.30 [-14.50, -8.10]
Thunga AV et al. 2022	-3	4.27	12	-2.66	5.76	12	7.0%	-0.34 [-4.40, 3.72]
Timo MB et al. 2021	3	30.01	14	1	22.82	16	1.1%	2.00 [-17.29, 21.29]
Wang et al. 2022	-7.43	7.45	23	-5.73	11.23	22	5.7%	-1.70 [-7.29, 3.89]
Witoon M et al. 2022	-0.8	13.5	17	2.6	20.28	16	2.4%	-3.40 [-15.23, 8.43]
Xiao et al. 2016	-3.1	20.49	45	1.5	16.19	44	4.2%	-4.60 [-12.26, 3.06]
Yin et al. 2025	-0.5	13.8	25	-2.35	12.28	26	4.5%	1.85 [-5.33, 9.03]
**Total (95% CI)**			**479**			**459**	**100.0%**	**-4.76 [-6.86, -2.67]**

Heterogeneity: Tau² = 12.02; Chi² = 66.40, df = 16 (P < 0.00001); I² = 76%
Test for overall effect: Z = 4.45 (P < 0.00001)

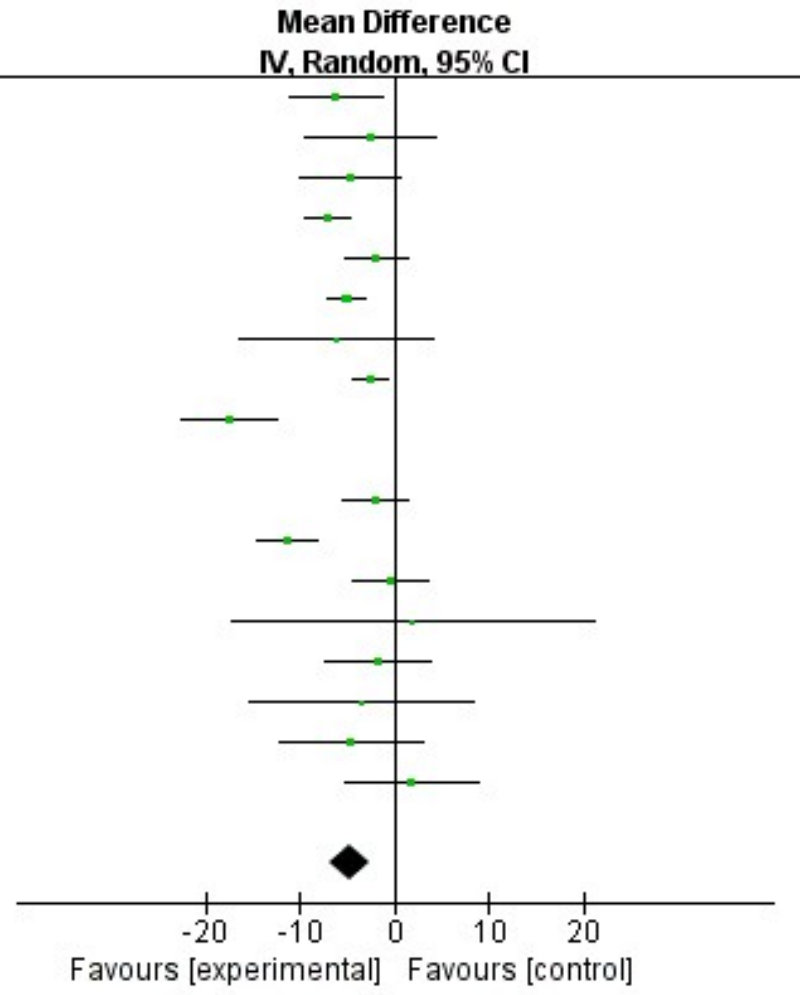

Study or Subgroup	Experimental Mean	Experimental SD	Experimental Total	Control Mean	Control SD	Control Total	Weight	Mean Difference IV, Random, 95% CI
Gao et al. 2014	-8.05	10.91	37	-1.9	11.25	39	6.5%	-6.15 [-11.13, -1.17]
Gloria VD et al. 2018	3.04	9.23	12	5.58	8.38	13	4.9%	-2.54 [-9.47, 4.39]
Kwok et al. 2019	-13.8	14.29	58	-9.11	15.15	59	6.2%	-4.69 [-10.03, 0.65]
Kwok et al. 2025	-8.01	6.33	52	-0.93	6.62	54	8.7%	-7.08 [-9.55, -4.61]
Lee et al. 2018	-1	6.8	25	0.9	3.77	16	8.1%	-1.90 [-5.14, 1.34]
Li et al. 2012	-6.42	5.02	65	-1.4	6.96	65	9.0%	-5.02 [-7.11, -2.93]
Li et al. 2022a	-8.68	15.9	19	-2.6	16.2	19	3.1%	-6.08 [-16.29, 4.13]
Li et al. 2022b	-2.2	1.7	15	0.25	3.34	16	9.2%	-2.45 [-4.30, -0.60]
Li et al. 2022c	-6.1	11.32	32	11.4	6.48	17	6.5%	-17.50 [-22.49, -12.51]
Li et al. 2024	-4.19	16.61	27	-0.22	16.13	27	3.8%	-3.97 [-12.70, 4.76]
Lin et al. 2023	-5	5.77	15	-3	3.3	15	0.0%	-2.00 [-5.36, 1.36]
Ni et al. 2016	-10.9	5.13	13	0.4	2.52	10	8.1%	-11.30 [-14.50, -8.10]
Thunga AV et al. 2022	-3	4.27	12	-2.66	5.76	12	7.3%	-0.34 [-4.40, 3.72]
Timo MB et al. 2021	3	30.01	14	1	22.82	16	1.1%	2.00 [-17.29, 21.29]
Wang et al. 2022	-7.43	7.45	23	-5.73	11.23	22	5.9%	-1.70 [-7.29, 3.89]
Witoon M et al. 2022	-0.8	13.5	17	2.6	20.28	16	2.5%	-3.40 [-15.23, 8.43]
Xiao et al. 2016	-3.1	20.49	45	1.5	16.19	44	4.4%	-4.60 [-12.26, 3.06]
Yin et al. 2025	-0.5	13.8	25	-2.35	12.28	26	4.7%	1.85 [-5.33, 9.03]
**Total (95% CI)**			**491**			**471**	**100.0%**	**-4.95 [-7.11, -2.79]**

Heterogeneity: Tau² = 12.29; Chi² = 63.82, df = 16 (P < 0.00001); I² = 75%
Test for overall effect: Z = 4.50 (P < 0.00001)

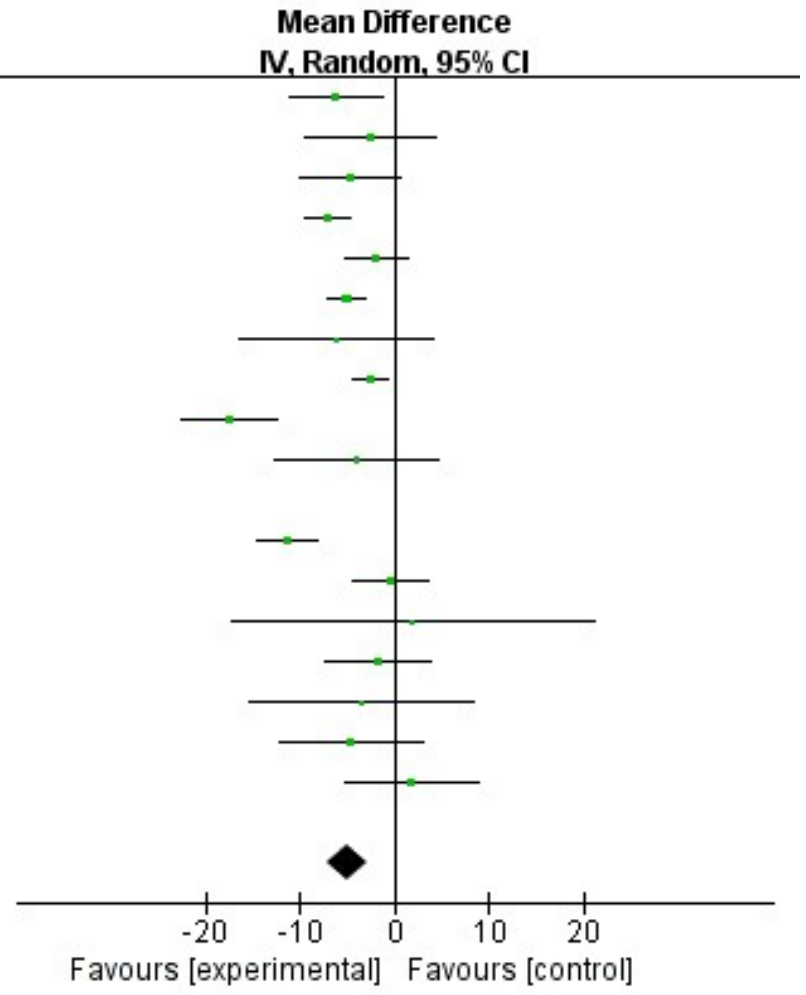

Study or Subgroup	Experimental Mean	Experimental SD	Experimental Total	Control Mean	Control SD	Control Total	Weight	Mean Difference IV, Random, 95% CI
Gao et al. 2014	-8.05	10.91	37	-1.9	11.25	39	6.4%	-6.15 [-11.13, -1.17]
Gloria VD et al. 2018	3.04	9.23	12	5.58	8.38	13	4.5%	-2.54 [-9.47, 4.39]
Kwok et al. 2019	-13.8	14.29	58	-9.11	15.15	59	6.0%	-4.69 [-10.03, 0.65]
Kwok et al. 2025	-8.01	6.33	52	-0.93	6.62	54	9.5%	-7.08 [-9.55, -4.61]
Lee et al. 2018	-1	6.8	25	0.9	3.77	16	8.5%	-1.90 [-5.14, 1.34]
Li et al. 2012	-6.42	5.02	65	-1.4	6.96	65	9.9%	-5.02 [-7.11, -2.93]
Li et al. 2022a	-8.68	15.9	19	-2.6	16.2	19	2.7%	-6.08 [-16.29, 4.13]
Li et al. 2022b	-2.2	1.7	15	0.25	3.34	16	10.2%	-2.45 [-4.30, -0.60]
Li et al. 2022c	-6.1	11.32	32	11.4	6.48	17	6.4%	-17.50 [-22.49, -12.51]
Li et al. 2024	-4.19	16.61	27	-0.22	16.13	27	3.3%	-3.97 [-12.70, 4.76]
Lin et al. 2023	-5	5.77	15	-3	3.3	15	8.3%	-2.00 [-5.36, 1.36]
Ni et al. 2016	-10.9	5.13	13	0.4	2.52	10	0.0%	-11.30 [-14.50, -8.10]
Thunga AV et al. 2022	-3	4.27	12	-2.66	5.76	12	7.4%	-0.34 [-4.40, 3.72]
Timo MB et al. 2021	3	30.01	14	1	22.82	16	0.9%	2.00 [-17.29, 21.29]
Wang et al. 2022	-7.43	7.45	23	-5.73	11.23	22	5.7%	-1.70 [-7.29, 3.89]
Witoon M et al. 2022	-0.8	13.5	17	2.6	20.28	16	2.1%	-3.40 [-15.23, 8.43]
Xiao et al. 2016	-3.1	20.49	45	1.5	16.19	44	4.0%	-4.60 [-12.26, 3.06]
Yin et al. 2025	-0.5	13.8	25	-2.35	12.28	26	4.3%	1.85 [-5.33, 9.03]
**Total (95% CI)**			**493**			**476**	**100.0%**	**-4.21 [-6.12, -2.31]**

Heterogeneity: Tau² = 8.44; Chi² = 48.51, df = 16 (P < 0.0001); I² = 67%
Test for overall effect: Z = 4.33 (P < 0.0001)

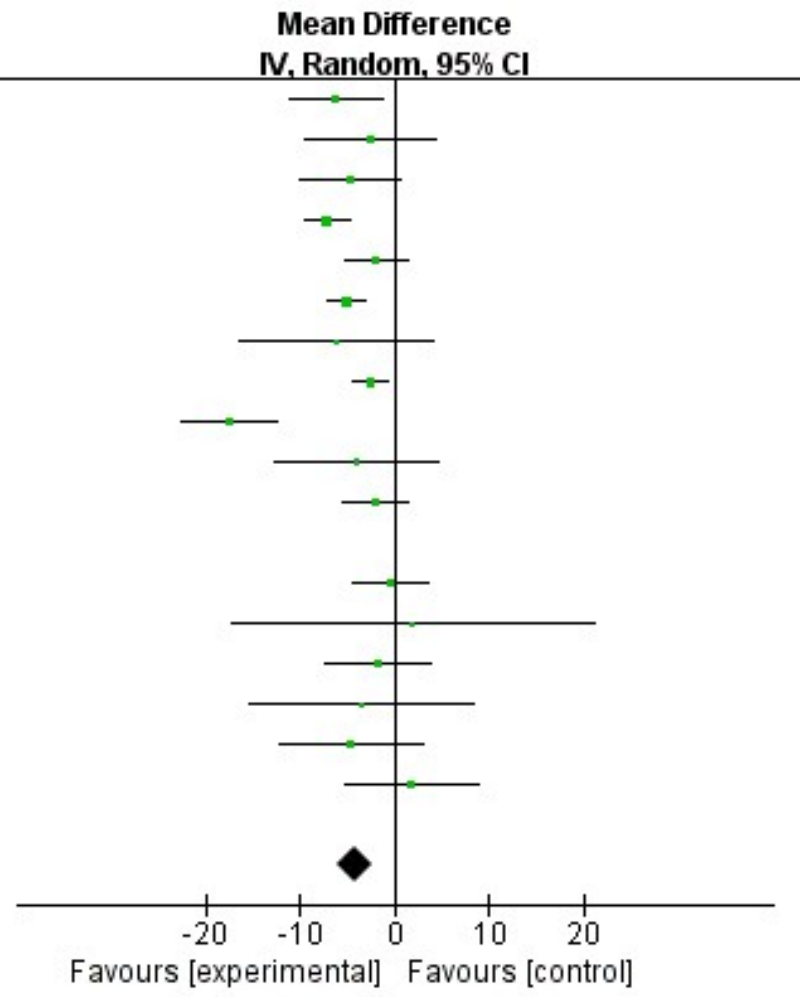

Study or Subgroup	Experimental Mean	Experimental SD	Experimental Total	Control Mean	Control SD	Control Total	Weight	Mean Difference IV, Random, 95% CI
Gao et al. 2014	-8.05	10.91	37	-1.9	11.25	39	6.4%	-6.15 [-11.13, -1.17]
Gloria VD et al. 2018	3.04	9.23	12	5.58	8.38	13	4.8%	-2.54 [-9.47, 4.39]
Kwok et al. 2019	-13.8	14.29	58	-9.11	15.15	59	6.1%	-4.69 [-10.03, 0.65]
Kwok et al. 2025	-8.01	6.33	52	-0.93	6.62	54	8.8%	-7.08 [-9.55, -4.61]
Lee et al. 2018	-1	6.8	25	0.9	3.77	16	8.1%	-1.90 [-5.14, 1.34]
Li et al. 2012	-6.42	5.02	65	-1.4	6.96	65	9.1%	-5.02 [-7.11, -2.93]
Li et al. 2022a	-8.68	15.9	19	-2.6	16.2	19	3.0%	-6.08 [-16.29, 4.13]
Li et al. 2022b	-2.2	1.7	15	0.25	3.34	16	9.3%	-2.45 [-4.30, -0.60]
Li et al. 2022c	-6.1	11.32	32	11.4	6.48	17	6.4%	-17.50 [-22.49, -12.51]
Li et al. 2024	-4.19	16.61	27	-0.22	16.13	27	3.7%	-3.97 [-12.70, 4.76]
Lin et al. 2023	-5	5.77	15	-3	3.3	15	8.0%	-2.00 [-5.36, 1.36]
Ni et al. 2016	-10.9	5.13	13	0.4	2.52	10	8.1%	-11.30 [-14.50, -8.10]
Thunga AV et al. 2022	-3	4.27	12	-2.66	5.76	12	0.0%	-0.34 [-4.40, 3.72]
Timo MB et al. 2021	3	30.01	14	1	22.82	16	1.1%	2.00 [-17.29, 21.29]
Wang et al. 2022	-7.43	7.45	23	-5.73	11.23	22	5.9%	-1.70 [-7.29, 3.89]
Witoon M et al. 2022	-0.8	13.5	17	2.6	20.28	16	2.4%	-3.40 [-15.23, 8.43]
Xiao et al. 2016	-3.1	20.49	45	1.5	16.19	44	4.3%	-4.60 [-12.26, 3.06]
Yin et al. 2025	-0.5	13.8	25	-2.35	12.28	26	4.6%	1.85 [-5.33, 9.03]
**Total (95% CI)**			**494**			**474**	**100.0%**	**-5.06 [-7.16, -2.96]**

Heterogeneity: Tau² = 11.46; Chi² = 61.83, df = 16 (P < 0.00001); I² = 74%
Test for overall effect: Z = 4.72 (P < 0.00001)

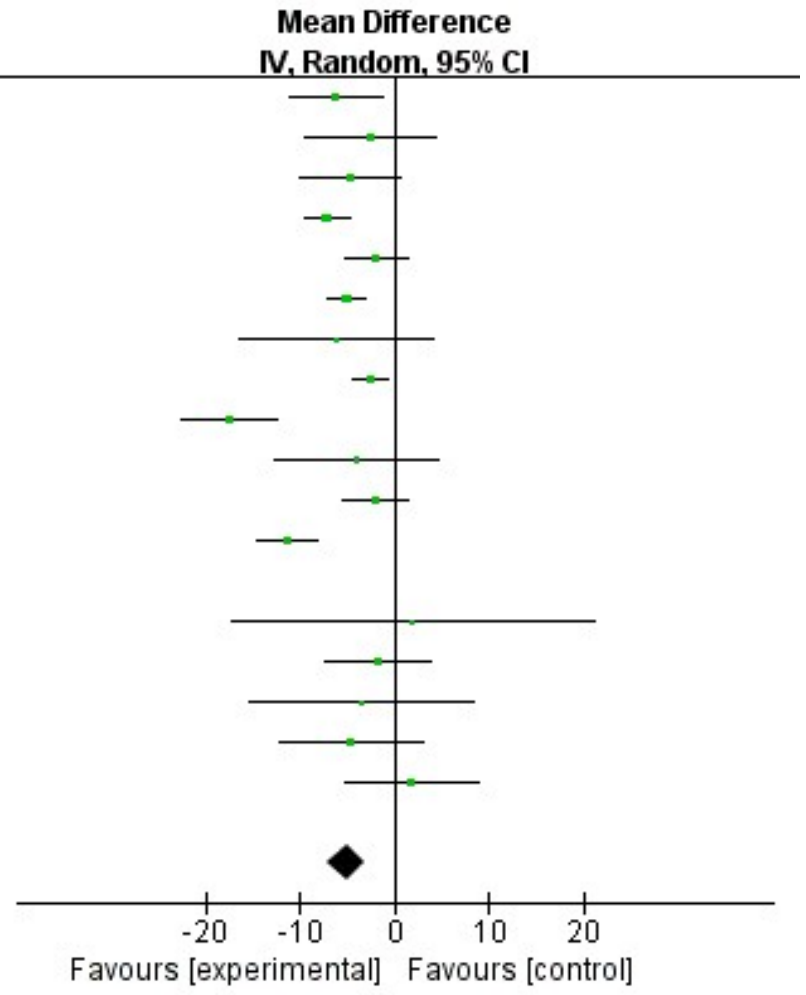

Study or Subgroup	Experimental Mean	Experimental SD	Experimental Total	Control Mean	Control SD	Control Total	Weight	Mean Difference IV, Random, 95% CI
Gao et al. 2014	-8.05	10.91	37	-1.9	11.25	39	6.0%	-6.15 [-11.13, -1.17]
Gloria VD et al. 2018	3.04	9.23	12	5.58	8.38	13	4.5%	-2.54 [-9.47, 4.39]
Kwok et al. 2019	-13.8	14.29	58	-9.11	15.15	59	5.7%	-4.69 [-10.03, 0.65]
Kwok et al. 2025	-8.01	6.33	52	-0.93	6.62	54	8.2%	-7.08 [-9.55, -4.61]
Lee et al. 2018	-1	6.8	25	0.9	3.77	16	7.6%	-1.90 [-5.14, 1.34]
Li et al. 2012	-6.42	5.02	65	-1.4	6.96	65	8.5%	-5.02 [-7.11, -2.93]
Li et al. 2022a	-8.68	15.9	19	-2.6	16.2	19	2.8%	-6.08 [-16.29, 4.13]
Li et al. 2022b	-2.2	1.7	15	0.25	3.34	16	8.7%	-2.45 [-4.30, -0.60]
Li et al. 2022c	-6.1	11.32	32	11.4	6.48	17	6.0%	-17.50 [-22.49, -12.51]
Li et al. 2024	-4.19	16.61	27	-0.22	16.13	27	3.5%	-3.97 [-12.70, 4.76]
Lin et al. 2023	-5	5.77	15	-3	3.3	15	7.5%	-2.00 [-5.36, 1.36]
Ni et al. 2016	-10.9	5.13	13	0.4	2.52	10	7.6%	-11.30 [-14.50, -8.10]
Thunga AV et al. 2022	-3	4.27	12	-2.66	5.76	12	6.9%	-0.34 [-4.40, 3.72]
Timo MB et al. 2021	3	30.01	14	1	22.82	16	0.0%	2.00 [-17.29, 21.29]
Wang et al. 2022	-7.43	7.45	23	-5.73	11.23	22	5.5%	-1.70 [-7.29, 3.89]
Witoon M et al. 2022	-0.8	13.5	17	2.6	20.28	16	2.3%	-3.40 [-15.23, 8.43]
Xiao et al. 2016	-3.1	20.49	45	1.5	16.19	44	4.1%	-4.60 [-12.26, 3.06]
Yin et al. 2025	-0.5	13.8	25	-2.35	12.28	26	4.4%	1.85 [-5.33, 9.03]
**Total (95% CI)**			**492**			**470**	**100.0%**	**-4.81 [-6.87, -2.75]**

Heterogeneity: Tau² = 11.79; Chi² = 65.96, df = 16 (P < 0.00001); I² = 76%
Test for overall effect: Z = 4.58 (P < 0.00001)

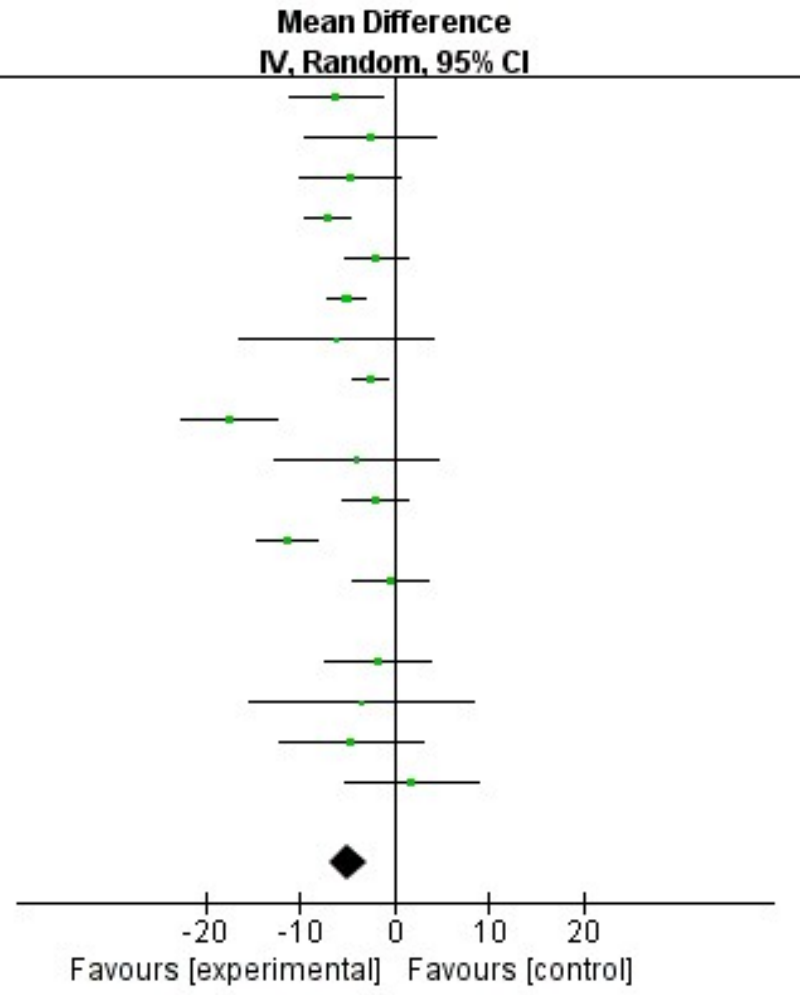

Study or Subgroup	Experimental Mean	Experimental SD	Experimental Total	Control Mean	Control SD	Control Total	Weight	Mean Difference IV, Random, 95% CI
Gao et al. 2014	-8.05	10.91	37	-1.9	11.25	39	6.3%	-6.15 [-11.13, -1.17]
Gloria VD et al. 2018	3.04	9.23	12	5.58	8.38	13	4.8%	-2.54 [-9.47, 4.39]
Kwok et al. 2019	-13.8	14.29	58	-9.11	15.15	59	6.0%	-4.69 [-10.03, 0.65]
Kwok et al. 2025	-8.01	6.33	52	-0.93	6.62	54	8.6%	-7.08 [-9.55, -4.61]
Lee et al. 2018	-1	6.8	25	0.9	3.77	16	7.9%	-1.90 [-5.14, 1.34]
Li et al. 2012	-6.42	5.02	65	-1.4	6.96	65	8.9%	-5.02 [-7.11, -2.93]
Li et al. 2022a	-8.68	15.9	19	-2.6	16.2	19	3.0%	-6.08 [-16.29, 4.13]
Li et al. 2022b	-2.2	1.7	15	0.25	3.34	16	9.1%	-2.45 [-4.30, -0.60]
Li et al. 2022c	-6.1	11.32	32	11.4	6.48	17	6.3%	-17.50 [-22.49, -12.51]
Li et al. 2024	-4.19	16.61	27	-0.22	16.13	27	3.7%	-3.97 [-12.70, 4.76]
Lin et al. 2023	-5	5.77	15	-3	3.3	15	7.8%	-2.00 [-5.36, 1.36]
Ni et al. 2016	-10.9	5.13	13	0.4	2.52	10	8.0%	-11.30 [-14.50, -8.10]
Thunga AV et al. 2022	-3	4.27	12	-2.66	5.76	12	7.2%	-0.34 [-4.40, 3.72]
Timo MB et al. 2021	3	30.01	14	1	22.82	16	1.1%	2.00 [-17.29, 21.29]
Wang et al. 2022	-7.43	7.45	23	-5.73	11.23	22	0.0%	-1.70 [-7.29, 3.89]
Witoon M et al. 2022	-0.8	13.5	17	2.6	20.28	16	2.4%	-3.40 [-15.23, 8.43]
Xiao et al. 2016	-3.1	20.49	45	1.5	16.19	44	4.3%	-4.60 [-12.26, 3.06]
Yin et al. 2025	-0.5	13.8	25	-2.35	12.28	26	4.6%	1.85 [-5.33, 9.03]
**Total (95% CI)**			**483**			**464**	**100.0%**	**-4.91 [-7.03, -2.79]**

Heterogeneity: Tau² = 11.98; Chi² = 65.31, df = 16 (P < 0.00001); I² = 76%
Test for overall effect: Z = 4.55 (P < 0.00001)

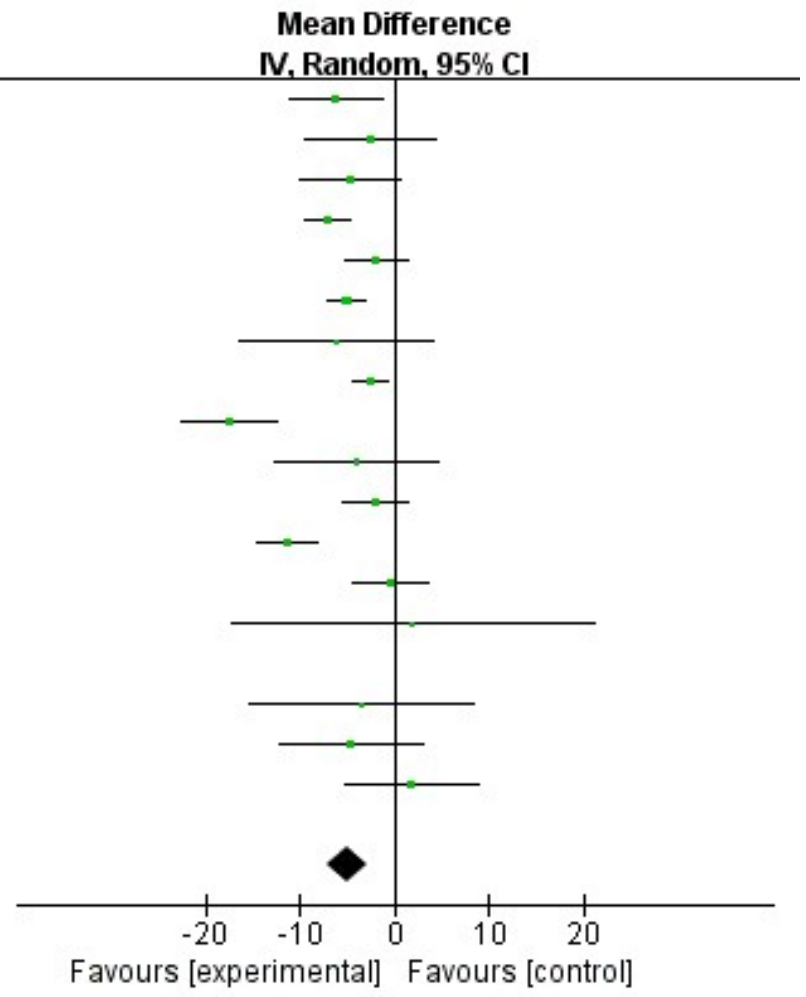

Study or Subgroup	Experimental Mean	Experimental SD	Experimental Total	Control Mean	Control SD	Control Total	Weight	Mean Difference IV, Random, 95% CI
Gao et al. 2014	-8.05	10.91	37	-1.9	11.25	39	6.1%	-6.15 [-11.13, -1.17]
Gloria VD et al. 2018	3.04	9.23	12	5.58	8.38	13	4.6%	-2.54 [-9.47, 4.39]
Kwok et al. 2019	-13.8	14.29	58	-9.11	15.15	59	5.8%	-4.69 [-10.03, 0.65]
Kwok et al. 2025	-8.01	6.33	52	-0.93	6.62	54	8.3%	-7.08 [-9.55, -4.61]
Lee et al. 2018	-1	6.8	25	0.9	3.77	16	7.7%	-1.90 [-5.14, 1.34]
Li et al. 2012	-6.42	5.02	65	-1.4	6.96	65	8.6%	-5.02 [-7.11, -2.93]
Li et al. 2022a	-8.68	15.9	19	-2.6	16.2	19	2.9%	-6.08 [-16.29, 4.13]
Li et al. 2022b	-2.2	1.7	15	0.25	3.34	16	8.8%	-2.45 [-4.30, -0.60]
Li et al. 2022c	-6.1	11.32	32	11.4	6.48	17	6.1%	-17.50 [-22.49, -12.51]
Li et al. 2024	-4.19	16.61	27	-0.22	16.13	27	3.5%	-3.97 [-12.70, 4.76]
Lin et al. 2023	-5	5.77	15	-3	3.3	15	7.6%	-2.00 [-5.36, 1.36]
Ni et al. 2016	-10.9	5.13	13	0.4	2.52	10	7.7%	-11.30 [-14.50, -8.10]
Thunga AV et al. 2022	-3	4.27	12	-2.66	5.76	12	6.9%	-0.34 [-4.40, 3.72]
Timo MB et al. 2021	3	30.01	14	1	22.82	16	1.0%	2.00 [-17.29, 21.29]
Wang et al. 2022	-7.43	7.45	23	-5.73	11.23	22	5.6%	-1.70 [-7.29, 3.89]
Witoon M et al. 2022	-0.8	13.5	17	2.6	20.28	16	0.0%	-3.40 [-15.23, 8.43]
Xiao et al. 2016	-3.1	20.49	45	1.5	16.19	44	4.1%	-4.60 [-12.26, 3.06]
Yin et al. 2025	-0.5	13.8	25	-2.35	12.28	26	4.4%	1.85 [-5.33, 9.03]
**Total (95% CI)**			**489**			**470**	**100.0%**	**-4.77 [-6.85, -2.69]**

Heterogeneity: Tau² = 11.94; Chi² = 66.38, df = 16 (P < 0.00001); I² = 76%
Test for overall effect: Z = 4.49 (P < 0.00001)

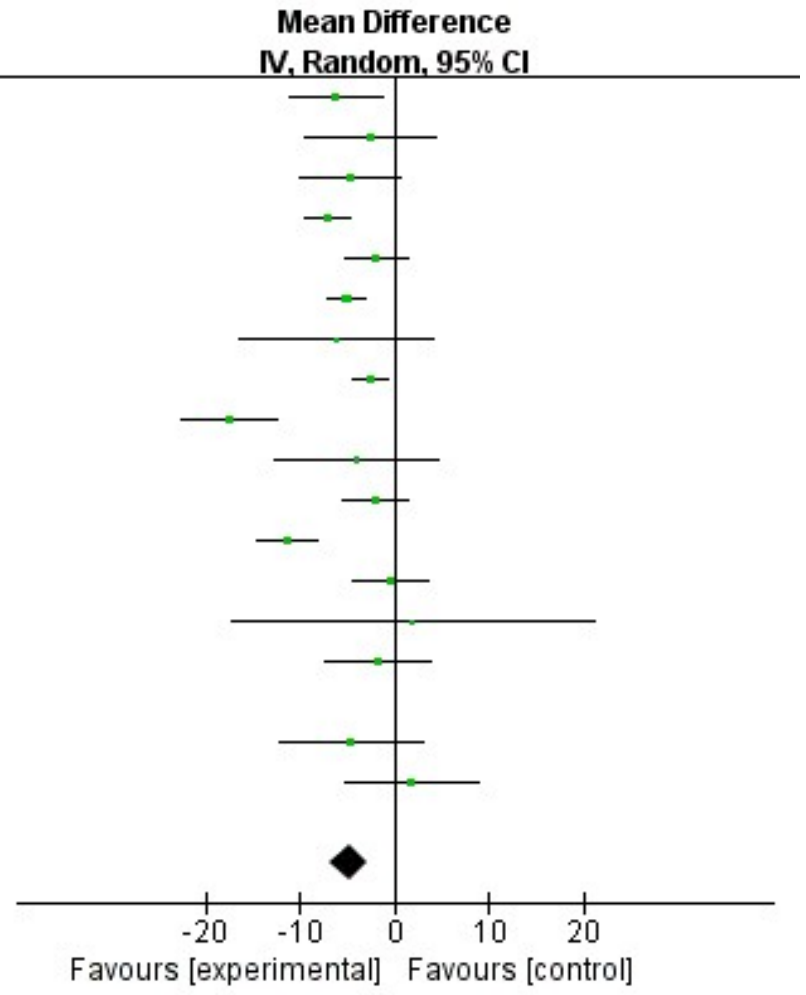

Study or Subgroup	Experimental Mean	Experimental SD	Experimental Total	Control Mean	Control SD	Control Total	Weight	Mean Difference IV, Random, 95% CI
Gao et al. 2014	-8.05	10.91	37	-1.9	11.25	39	6.2%	-6.15 [-11.13, -1.17]
Gloria VD et al. 2018	3.04	9.23	12	5.58	8.38	13	4.7%	-2.54 [-9.47, 4.39]
Kwok et al. 2019	-13.8	14.29	58	-9.11	15.15	59	5.9%	-4.69 [-10.03, 0.65]
Kwok et al. 2025	-8.01	6.33	52	-0.93	6.62	54	8.5%	-7.08 [-9.55, -4.61]
Lee et al. 2018	-1	6.8	25	0.9	3.77	16	7.8%	-1.90 [-5.14, 1.34]
Li et al. 2012	-6.42	5.02	65	-1.4	6.96	65	8.8%	-5.02 [-7.11, -2.93]
Li et al. 2022a	-8.68	15.9	19	-2.6	16.2	19	3.0%	-6.08 [-16.29, 4.13]
Li et al. 2022b	-2.2	1.7	15	0.25	3.34	16	8.9%	-2.45 [-4.30, -0.60]
Li et al. 2022c	-6.1	11.32	32	11.4	6.48	17	6.2%	-17.50 [-22.49, -12.51]
Li et al. 2024	-4.19	16.61	27	-0.22	16.13	27	3.6%	-3.97 [-12.70, 4.76]
Lin et al. 2023	-5	5.77	15	-3	3.3	15	7.7%	-2.00 [-5.36, 1.36]
Ni et al. 2016	-10.9	5.13	13	0.4	2.52	10	7.9%	-11.30 [-14.50, -8.10]
Thunga AV et al. 2022	-3	4.27	12	-2.66	5.76	12	7.1%	-0.34 [-4.40, 3.72]
Timo MB et al. 2021	3	30.01	14	1	22.82	16	1.1%	2.00 [-17.29, 21.29]
Wang et al. 2022	-7.43	7.45	23	-5.73	11.23	22	5.7%	-1.70 [-7.29, 3.89]
Witoon M et al. 2022	-0.8	13.5	17	2.6	20.28	16	2.4%	-3.40 [-15.23, 8.43]
Xiao et al. 2016	-3.1	20.49	45	1.5	16.19	44	0.0%	-4.60 [-12.26, 3.06]
Yin et al. 2025	-0.5	13.8	25	-2.35	12.28	26	4.5%	1.85 [-5.33, 9.03]
**Total (95% CI)**			**461**			**442**	**100.0%**	**-4.74 [-6.85, -2.63]**

Heterogeneity: Tau² = 12.07; Chi² = 66.42, df = 16 (P < 0.00001); I² = 76%
Test for overall effect: Z = 4.41 (P < 0.0001)

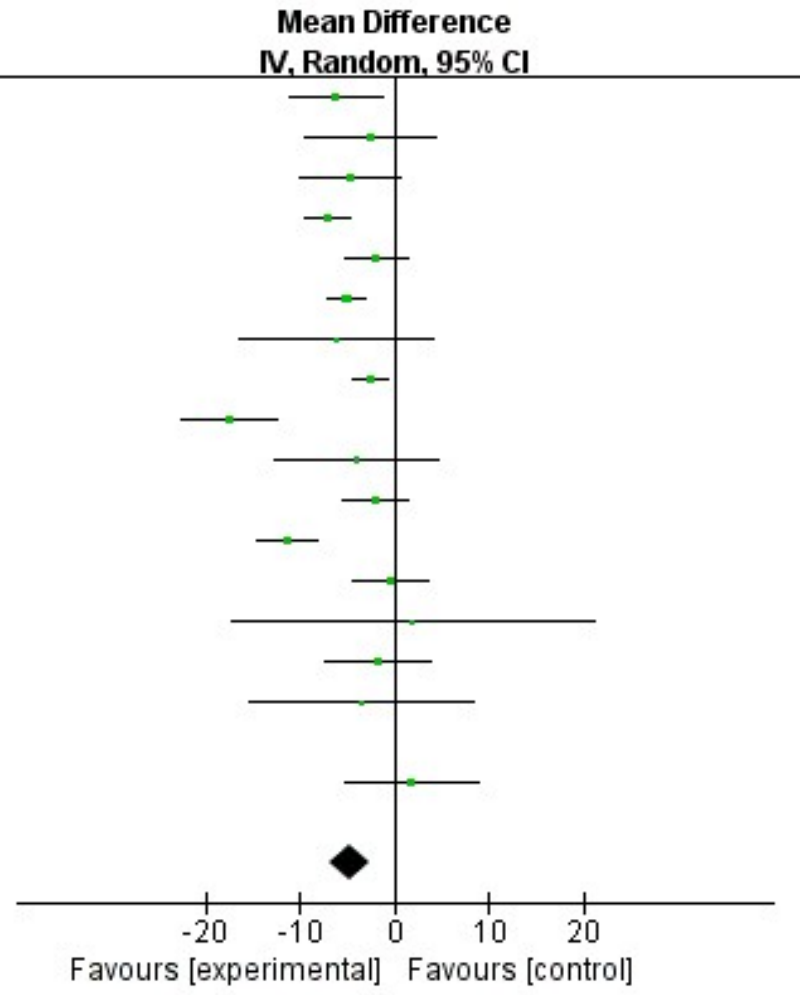

Study or Subgroup	Experimental Mean	Experimental SD	Experimental Total	Control Mean	Control SD	Control Total	Weight	Mean Difference IV, Random, 95% CI
Gao et al. 2014	-8.05	10.91	37	-1.9	11.25	39	6.2%	-6.15 [-11.13, -1.17]
Gloria VD et al. 2018	3.04	9.23	12	5.58	8.38	13	4.7%	-2.54 [-9.47, 4.39]
Kwok et al. 2019	-13.8	14.29	58	-9.11	15.15	59	5.9%	-4.69 [-10.03, 0.65]
Kwok et al. 2025	-8.01	6.33	52	-0.93	6.62	54	8.6%	-7.08 [-9.55, -4.61]
Lee et al. 2018	-1	6.8	25	0.9	3.77	16	7.9%	-1.90 [-5.14, 1.34]
Li et al. 2012	-6.42	5.02	65	-1.4	6.96	65	8.9%	-5.02 [-7.11, -2.93]
Li et al. 2022a	-8.68	15.9	19	-2.6	16.2	19	2.9%	-6.08 [-16.29, 4.13]
Li et al. 2022b	-2.2	1.7	15	0.25	3.34	16	9.1%	-2.45 [-4.30, -0.60]
Li et al. 2022c	-6.1	11.32	32	11.4	6.48	17	6.2%	-17.50 [-22.49, -12.51]
Li et al. 2024	-4.19	16.61	27	-0.22	16.13	27	3.6%	-3.97 [-12.70, 4.76]
Lin et al. 2023	-5	5.77	15	-3	3.3	15	7.8%	-2.00 [-5.36, 1.36]
Ni et al. 2016	-10.9	5.13	13	0.4	2.52	10	7.9%	-11.30 [-14.50, -8.10]
Thunga AV et al. 2022	-3	4.27	12	-2.66	5.76	12	7.1%	-0.34 [-4.40, 3.72]
Timo MB et al. 2021	3	30.01	14	1	22.82	16	1.0%	2.00 [-17.29, 21.29]
Wang et al. 2022	-7.43	7.45	23	-5.73	11.23	22	5.7%	-1.70 [-7.29, 3.89]
Witoon M et al. 2022	-0.8	13.5	17	2.6	20.28	16	2.3%	-3.40 [-15.23, 8.43]
Xiao et al. 2016	-3.1	20.49	45	1.5	16.19	44	4.2%	-4.60 [-12.26, 3.06]
Yin et al. 2025	-0.5	13.8	25	-2.35	12.28	26	0.0%	1.85 [-5.33, 9.03]
**Total (95% CI)**			**481**			**460**	**100.0%**	**-5.04 [-7.10, -2.97]**

Heterogeneity: Tau² = 11.33; Chi² = 63.21, df = 16 (P < 0.00001); I² = 75%
Test for overall effect: Z = 4.78 (P < 0.00001)

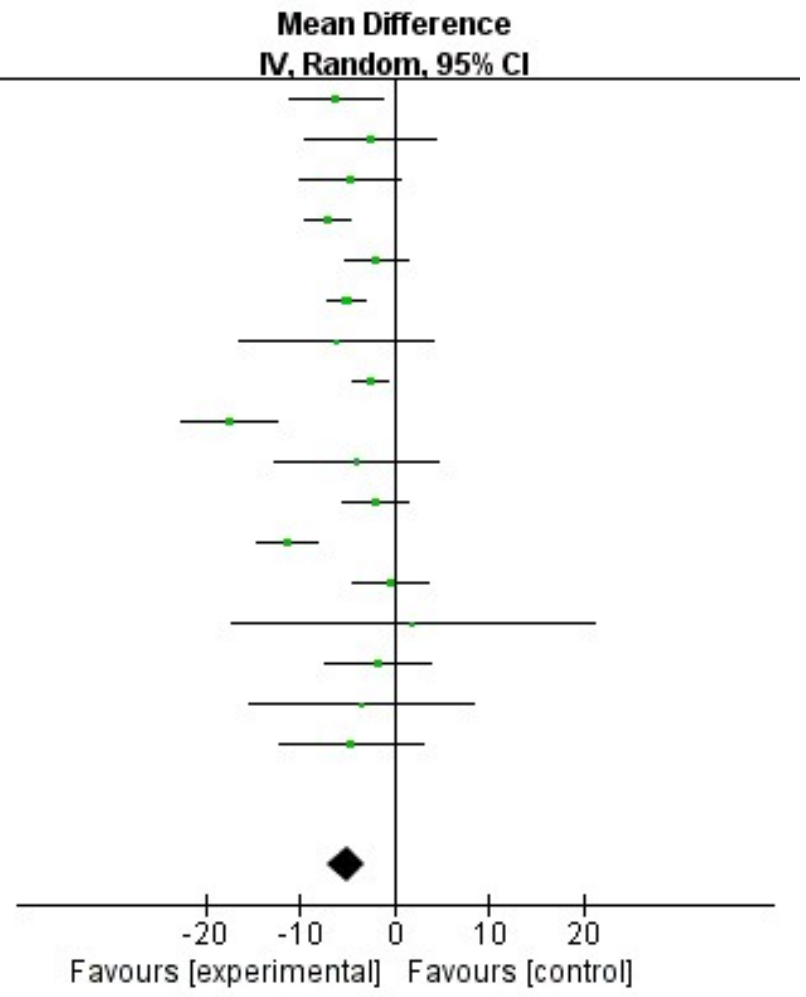

eFigure 2. shows the sensitivity analysis of TUG

	Experimental			Control				Mean Difference
Study or Subgroup	Mean	SD	Total	Mean	SD	Total	Weight	IV, Random, 95% CI
Choi et al. 2016	-0.36	0.9	11	1.26	3.7	9	0.0%	-1.62 [-4.10, 0.86]
Gao et al. 2014	-1.37	2.93	37	0.03	2.85	39	11.7%	-1.40 [-2.70, -0.10]
Gloria VD et al. 2018	-0.58	2.62	12	0	1.78	13	9.4%	-0.58 [-2.35, 1.19]
Kwok et al. 2019	-2.82	15.39	58	-1.64	5.61	59	3.2%	-1.18 [-5.39, 3.03]
Li et al. 2012	-1.05	2.8	65	-0.02	3.32	65	13.0%	-1.03 [-2.09, 0.03]
Li et al. 2022a	-1.38	3.03	19	1.95	4.22	19	7.2%	-3.33 [-5.67, -0.99]
Li et al. 2022b	-2.49	2.26	15	-0.17	6.34	16	4.6%	-2.32 [-5.63, 0.99]
Li et al. 2022c	-1.78	2.98	32	-0.26	1.71	17	11.6%	-1.52 [-2.83, -0.21]
Liu et al. 2016	-4.27	2.41	23	-0.1	2.77	18	10.1%	-4.17 [-5.78, -2.56]
Ni et al. 2016	-2.3	2.9	13	0.3	0.84	10	9.9%	-2.60 [-4.26, -0.94]
Wan et al. 2021	-2.21	4.09	20	0.94	5.79	20	5.0%	-3.15 [-6.26, -0.04]
Wang et al. 2022	-0.42	2.01	23	-1.4	4.85	22	7.7%	0.98 [-1.21, 3.17]
Witoon M et al. 2022	1	5.02	17	-0.6	4.04	16	5.0%	1.60 [-1.50, 4.70]
Xiao et al. 2016	-1.2	11.65	45	1	17.83	44	1.6%	-2.20 [-8.47, 4.07]
**Total (95% CI)**			**379**			**358**	**100.0%**	**-1.63 [-2.46, -0.80]**

Heterogeneity: Tau² = 1.10; Chi² = 26.38, df = 12 (P = 0.009); I² = 55%
Test for overall effect: Z = 3.83 (P = 0.0001)

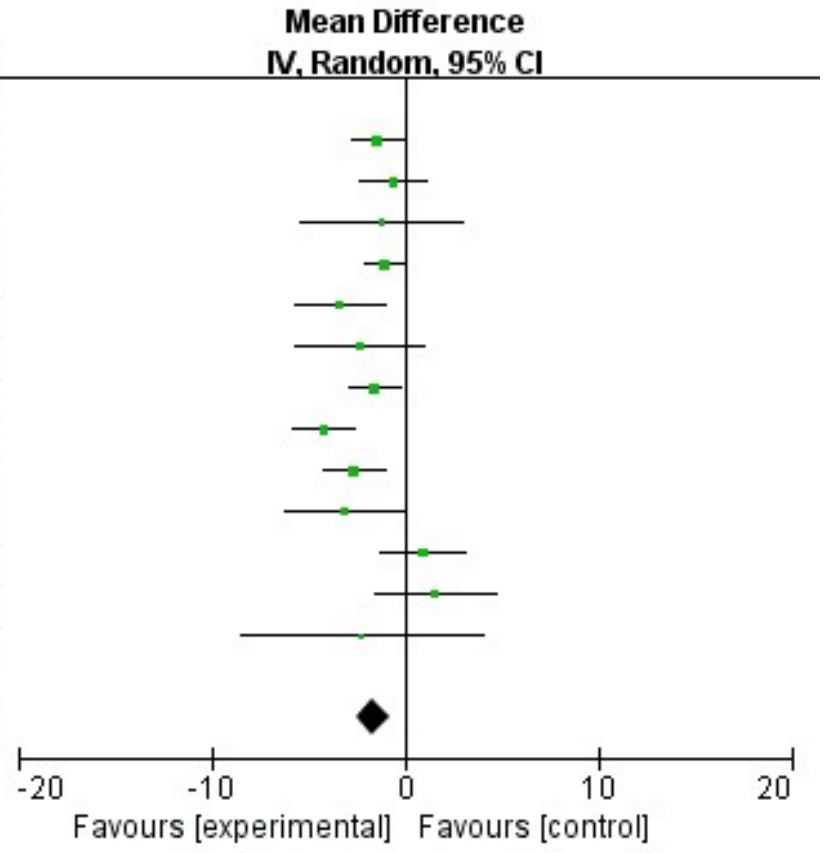

	Experimental			Control				Mean Difference
Study or Subgroup	Mean	SD	Total	Mean	SD	Total	Weight	IV, Random, 95% CI
Choi et al. 2016	-0.36	0.9	11	1.26	3.7	9	7.2%	-1.62 [-4.10, 0.86]
Gao et al. 2014	-1.37	2.93	37	0.03	2.85	39	0.0%	-1.40 [-2.70, -0.10]
Gloria VD et al. 2018	-0.58	2.62	12	0	1.78	13	9.9%	-0.58 [-2.35, 1.19]
Kwok et al. 2019	-2.82	15.39	58	-1.64	5.61	59	3.5%	-1.18 [-5.39, 3.03]
Li et al. 2012	-1.05	2.8	65	-0.02	3.32	65	13.3%	-1.03 [-2.09, 0.03]
Li et al. 2022a	-1.38	3.03	19	1.95	4.22	19	7.6%	-3.33 [-5.67, -0.99]
Li et al. 2022b	-2.49	2.26	15	-0.17	6.34	16	5.0%	-2.32 [-5.63, 0.99]
Li et al. 2022c	-1.78	2.98	32	-0.26	1.71	17	12.0%	-1.52 [-2.83, -0.21]
Liu et al. 2016	-4.27	2.41	23	-0.1	2.77	18	10.6%	-4.17 [-5.78, -2.56]
Ni et al. 2016	-2.3	2.9	13	0.3	0.84	10	10.4%	-2.60 [-4.26, -0.94]
Wan et al. 2021	-2.21	4.09	20	0.94	5.79	20	5.4%	-3.15 [-6.26, -0.04]
Wang et al. 2022	-0.42	2.01	23	-1.4	4.85	22	8.2%	0.98 [-1.21, 3.17]
Witoon M et al. 2022	1	5.02	17	-0.6	4.04	16	5.4%	1.60 [-1.50, 4.70]
Xiao et al. 2016	-1.2	11.65	45	1	17.83	44	1.8%	-2.20 [-8.47, 4.07]
**Total (95% CI)**			**353**			**328**	**100.0%**	**-1.65 [-2.54, -0.77]**

Heterogeneity: Tau² = 1.24; Chi² = 26.27, df = 12 (P = 0.010); I² = 54%
Test for overall effect: Z = 3.68 (P = 0.0002)

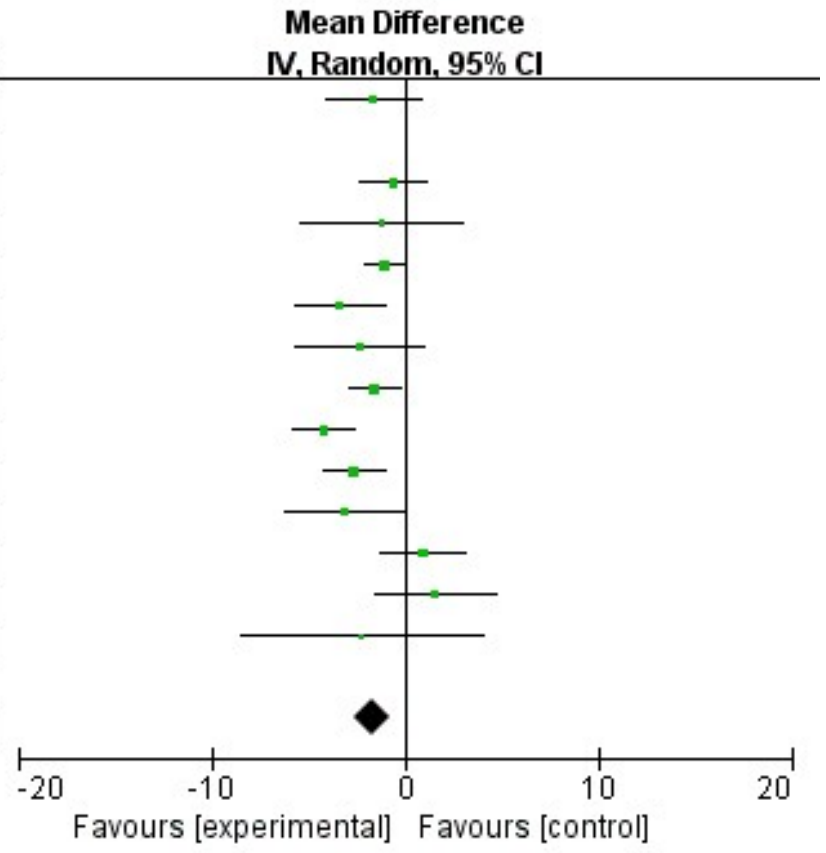

	Experimental			Control				Mean Difference
Study or Subgroup	Mean	SD	Total	Mean	SD	Total	Weight	IV, Random, 95% CI
Choi et al. 2016	-0.36	0.9	11	1.26	3.7	9	6.8%	-1.62 [-4.10, 0.86]
Gao et al. 2014	-1.37	2.93	37	0.03	2.85	39	12.1%	-1.40 [-2.70, -0.10]
Gloria VD et al. 2018	-0.58	2.62	12	0	1.78	13	0.0%	-0.58 [-2.35, 1.19]
Kwok et al. 2019	-2.82	15.39	58	-1.64	5.61	59	3.2%	-1.18 [-5.39, 3.03]
Li et al. 2012	-1.05	2.8	65	-0.02	3.32	65	13.5%	-1.03 [-2.09, 0.03]
Li et al. 2022a	-1.38	3.03	19	1.95	4.22	19	7.3%	-3.33 [-5.67, -0.99]
Li et al. 2022b	-2.49	2.26	15	-0.17	6.34	16	4.6%	-2.32 [-5.63, 0.99]
Li et al. 2022c	-1.78	2.98	32	-0.26	1.71	17	12.1%	-1.52 [-2.83, -0.21]
Liu et al. 2016	-4.27	2.41	23	-0.1	2.77	18	10.5%	-4.17 [-5.78, -2.56]
Ni et al. 2016	-2.3	2.9	13	0.3	0.84	10	10.2%	-2.60 [-4.26, -0.94]
Wan et al. 2021	-2.21	4.09	20	0.94	5.79	20	5.1%	-3.15 [-6.26, -0.04]
Wang et al. 2022	-0.42	2.01	23	-1.4	4.85	22	7.9%	0.98 [-1.21, 3.17]
Witoon M et al. 2022	1	5.02	17	-0.6	4.04	16	5.1%	1.60 [-1.50, 4.70]
Xiao et al. 2016	-1.2	11.65	45	1	17.83	44	1.6%	-2.20 [-8.47, 4.07]
**Total (95% CI)**			**378**			**354**	**100.0%**	**-1.73 [-2.56, -0.90]**

Heterogeneity: Tau² = 1.04; Chi² = 24.99, df = 12 (P = 0.01); I² = 52%
Test for overall effect: Z = 4.08 (P < 0.0001)

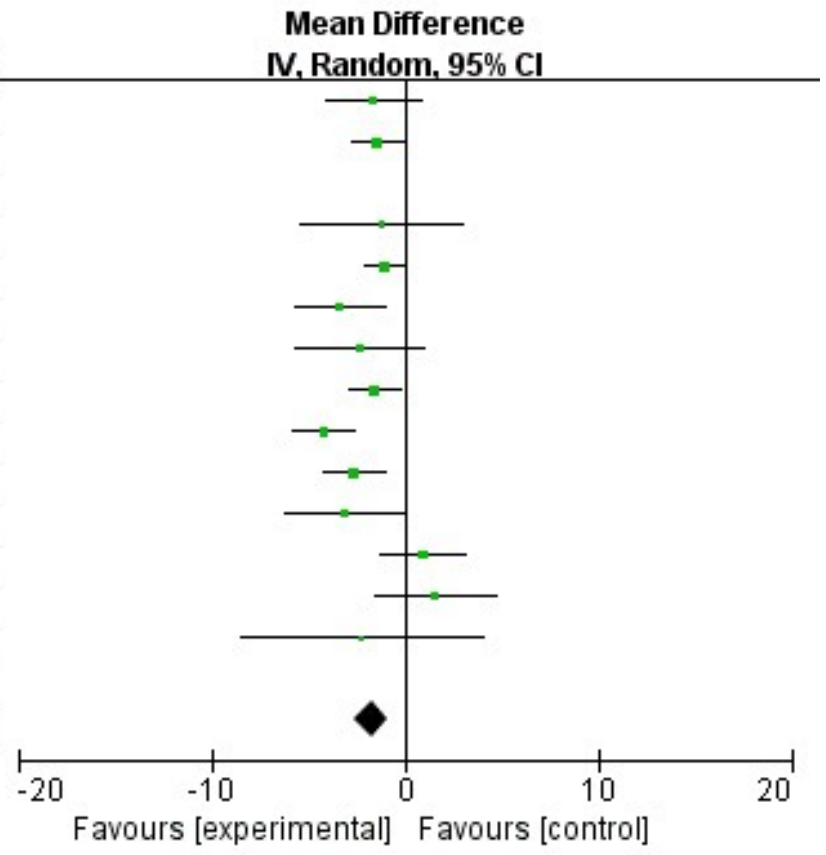

	Experimental			Control				Mean Difference
**Study or Subgroup**	**Mean**	**SD**	**Total**	**Mean**	**SD**	**Total**	**Weight**	**IV, Random, 95% CI**
Choi et al. 2016	-0.36	0.9	11	1.26	3.7	9	6.4%	-1.62 [-4.10, 0.86]
Gao et al. 2014	-1.37	2.93	37	0.03	2.85	39	11.4%	-1.40 [-2.70, -0.10]
Gloria VD et al. 2018	-0.58	2.62	12	0	1.78	13	9.1%	-0.58 [-2.35, 1.19]
Kwok et al. 2019	-2.82	15.39	58	-1.64	5.61	59	0.0%	-1.18 [-5.39, 3.03]
Li et al. 2012	-1.05	2.8	65	-0.02	3.32	65	12.6%	-1.03 [-2.09, 0.03]
Li et al. 2022a	-1.38	3.03	19	1.95	4.22	19	6.9%	-3.33 [-5.67, -0.99]
Li et al. 2022b	-2.49	2.26	15	-0.17	6.34	16	4.4%	-2.32 [-5.63, 0.99]
Li et al. 2022c	-1.78	2.98	32	-0.26	1.71	17	11.3%	-1.52 [-2.83, -0.21]
Liu et al. 2016	-4.27	2.41	23	-0.1	2.77	18	9.8%	-4.17 [-5.78, -2.56]
Ni et al. 2016	-2.3	2.9	13	0.3	0.84	10	9.6%	-2.60 [-4.26, -0.94]
Wan et al. 2021	-2.21	4.09	20	0.94	5.79	20	4.8%	-3.15 [-6.26, -0.04]
Wang et al. 2022	-0.42	2.01	23	-1.4	4.85	22	7.4%	0.98 [-1.21, 3.17]
Witoon M et al. 2022	1	5.02	17	-0.6	4.04	16	4.8%	1.60 [-1.50, 4.70]
Xiao et al. 2016	-1.2	11.65	45	1	17.83	44	1.5%	-2.20 [-8.47, 4.07]
**Total (95% CI)**			**332**			**308**	**100.0%**	**-1.64 [-2.45, -0.83]**

Heterogeneity: Tau² = 1.06; Chi² = 26.34, df = 12 (P = 0.010); I² = 54%
Test for overall effect: Z = 3.97 (P < 0.0001)

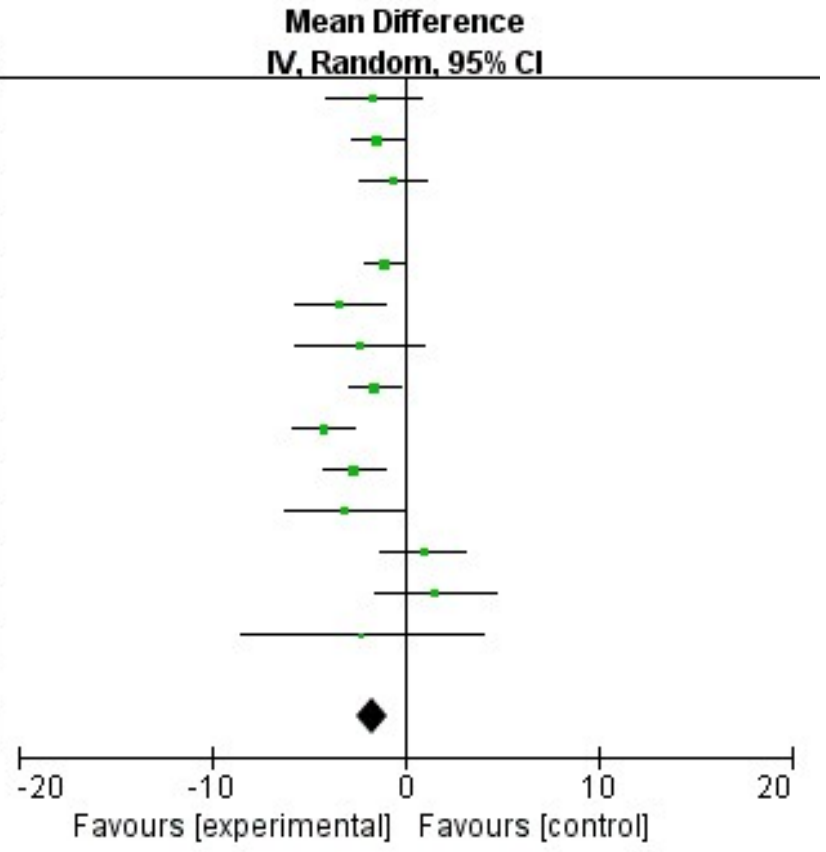

	Experimental			Control				Mean Difference
**Study or Subgroup**	**Mean**	**SD**	**Total**	**Mean**	**SD**	**Total**	**Weight**	**IV, Random, 95% CI**
Choi et al. 2016	-0.36	0.9	11	1.26	3.7	9	7.2%	-1.62 [-4.10, 0.86]
Gao et al. 2014	-1.37	2.93	37	0.03	2.85	39	12.3%	-1.40 [-2.70, -0.10]
Gloria VD et al. 2018	-0.58	2.62	12	0	1.78	13	10.0%	-0.58 [-2.35, 1.19]
Kwok et al. 2019	-2.82	15.39	58	-1.64	5.61	59	3.5%	-1.18 [-5.39, 3.03]
Li et al. 2012	-1.05	2.8	65	-0.02	3.32	65	0.0%	-1.03 [-2.09, 0.03]
Li et al. 2022a	-1.38	3.03	19	1.95	4.22	19	7.7%	-3.33 [-5.67, -0.99]
Li et al. 2022b	-2.49	2.26	15	-0.17	6.34	16	5.0%	-2.32 [-5.63, 0.99]
Li et al. 2022c	-1.78	2.98	32	-0.26	1.71	17	12.2%	-1.52 [-2.83, -0.21]
Liu et al. 2016	-4.27	2.41	23	-0.1	2.77	18	10.7%	-4.17 [-5.78, -2.56]
Ni et al. 2016	-2.3	2.9	13	0.3	0.84	10	10.5%	-2.60 [-4.26, -0.94]
Wan et al. 2021	-2.21	4.09	20	0.94	5.79	20	5.4%	-3.15 [-6.26, -0.04]
Wang et al. 2022	-0.42	2.01	23	-1.4	4.85	22	8.2%	0.98 [-1.21, 3.17]
Witoon M et al. 2022	1	5.02	17	-0.6	4.04	16	5.5%	1.60 [-1.50, 4.70]
Xiao et al. 2016	-1.2	11.65	45	1	17.83	44	1.8%	-2.20 [-8.47, 4.07]
**Total (95% CI)**			**325**			**302**	**100.0%**	**-1.71 [-2.59, -0.83]**

Heterogeneity: Tau² = 1.21; Chi² = 24.93, df = 12 (P = 0.02); I² = 52%
Test for overall effect: Z = 3.80 (P = 0.0001)

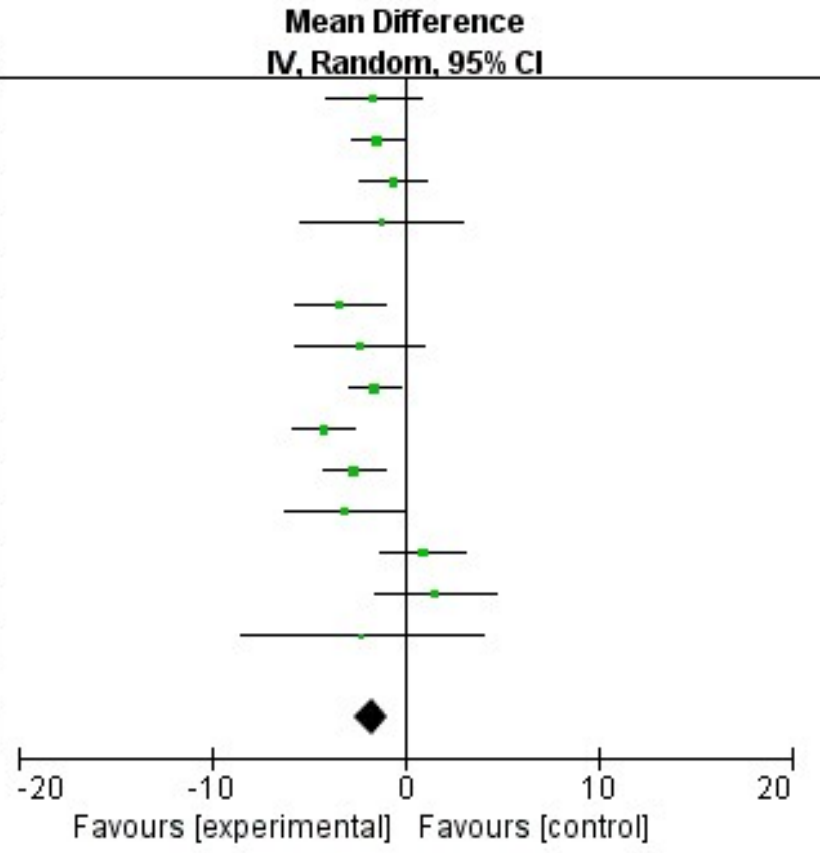

	Experimental			Control				Mean Difference
**Study or Subgroup**	**Mean**	**SD**	**Total**	**Mean**	**SD**	**Total**	**Weight**	**IV, Random, 95% CI**
Choi et al. 2016	-0.36	0.9	11	1.26	3.7	9	6.6%	-1.62 [-4.10, 0.86]
Gao et al. 2014	-1.37	2.93	37	0.03	2.85	39	12.1%	-1.40 [-2.70, -0.10]
Gloria VD et al. 2018	-0.58	2.62	12	0	1.78	13	9.5%	-0.58 [-2.35, 1.19]
Kwok et al. 2019	-2.82	15.39	58	-1.64	5.61	59	3.0%	-1.18 [-5.39, 3.03]
Li et al. 2012	-1.05	2.8	65	-0.02	3.32	65	13.5%	-1.03 [-2.09, 0.03]
Li et al. 2022a	-1.38	3.03	19	1.95	4.22	19	0.0%	-3.33 [-5.67, -0.99]
Li et al. 2022b	-2.49	2.26	15	-0.17	6.34	16	4.4%	-2.32 [-5.63, 0.99]
Li et al. 2022c	-1.78	2.98	32	-0.26	1.71	17	12.0%	-1.52 [-2.83, -0.21]
Liu et al. 2016	-4.27	2.41	23	-0.1	2.77	18	10.3%	-4.17 [-5.78, -2.56]
Ni et al. 2016	-2.3	2.9	13	0.3	0.84	10	10.0%	-2.60 [-4.26, -0.94]
Wan et al. 2021	-2.21	4.09	20	0.94	5.79	20	4.8%	-3.15 [-6.26, -0.04]
Wang et al. 2022	-0.42	2.01	23	-1.4	4.85	22	7.6%	0.98 [-1.21, 3.17]
Witoon M et al. 2022	1	5.02	17	-0.6	4.04	16	4.8%	1.60 [-1.50, 4.70]
Xiao et al. 2016	-1.2	11.65	45	1	17.83	44	1.5%	-2.20 [-8.47, 4.07]
**Total (95% CI)**			**371**			**348**	**100.0%**	**-1.51 [-2.31, -0.71]**

Heterogeneity: Tau² = 0.94; Chi² = 24.18, df = 12 (P = 0.02); I² = 50%
Test for overall effect: Z = 3.70 (P = 0.0002)

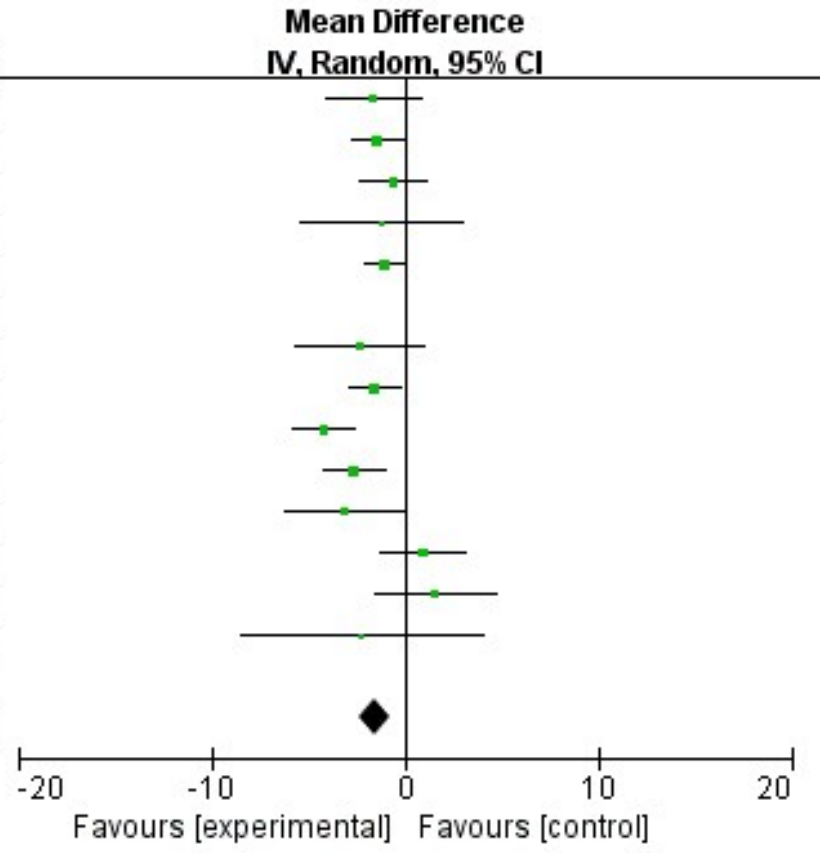

Study or Subgroup	Experimental Mean	Experimental SD	Experimental Total	Control Mean	Control SD	Control Total	Weight	Mean Difference IV, Random, 95% CI
Choi et al. 2016	-0.36	0.9	11	1.26	3.7	9	6.5%	-1.62 [-4.10, 0.86]
Gao et al. 2014	-1.37	2.93	37	0.03	2.85	39	11.5%	-1.40 [-2.70, -0.10]
Gloria VD et al. 2018	-0.58	2.62	12	0	1.78	13	9.2%	-0.58 [-2.35, 1.19]
Kwok et al. 2019	-2.82	15.39	58	-1.64	5.61	59	3.1%	-1.18 [-5.39, 3.03]
Li et al. 2012	-1.05	2.8	65	-0.02	3.32	65	12.8%	-1.03 [-2.09, 0.03]
Li et al. 2022a	-1.38	3.03	19	1.95	4.22	19	7.0%	-3.33 [-5.67, -0.99]
Li et al. 2022b	-2.49	2.26	15	-0.17	6.34	16	0.0%	-2.32 [-5.63, 0.99]
Li et al. 2022c	-1.78	2.98	32	-0.26	1.71	17	11.5%	-1.52 [-2.83, -0.21]
Liu et al. 2016	-4.27	2.41	23	-0.1	2.77	18	9.9%	-4.17 [-5.78, -2.56]
Ni et al. 2016	-2.3	2.9	13	0.3	0.84	10	9.7%	-2.60 [-4.26, -0.94]
Wan et al. 2021	-2.21	4.09	20	0.94	5.79	20	4.8%	-3.15 [-6.26, -0.04]
Wang et al. 2022	-0.42	2.01	23	-1.4	4.85	22	7.5%	0.98 [-1.21, 3.17]
Witoon M et al. 2022	1	5.02	17	-0.6	4.04	16	4.9%	1.60 [-1.50, 4.70]
Xiao et al. 2016	-1.2	11.65	45	1	17.83	44	1.5%	-2.20 [-8.47, 4.07]
**Total (95% CI)**			**375**			**351**	**100.0%**	**-1.60 [-2.41, -0.78]**

Heterogeneity: $Tau^2 = 1.06$; $Chi^2 = 26.20$, df = 12 (P = 0.01); $I^2 = 54\%$
Test for overall effect: Z = 3.84 (P = 0.0001)

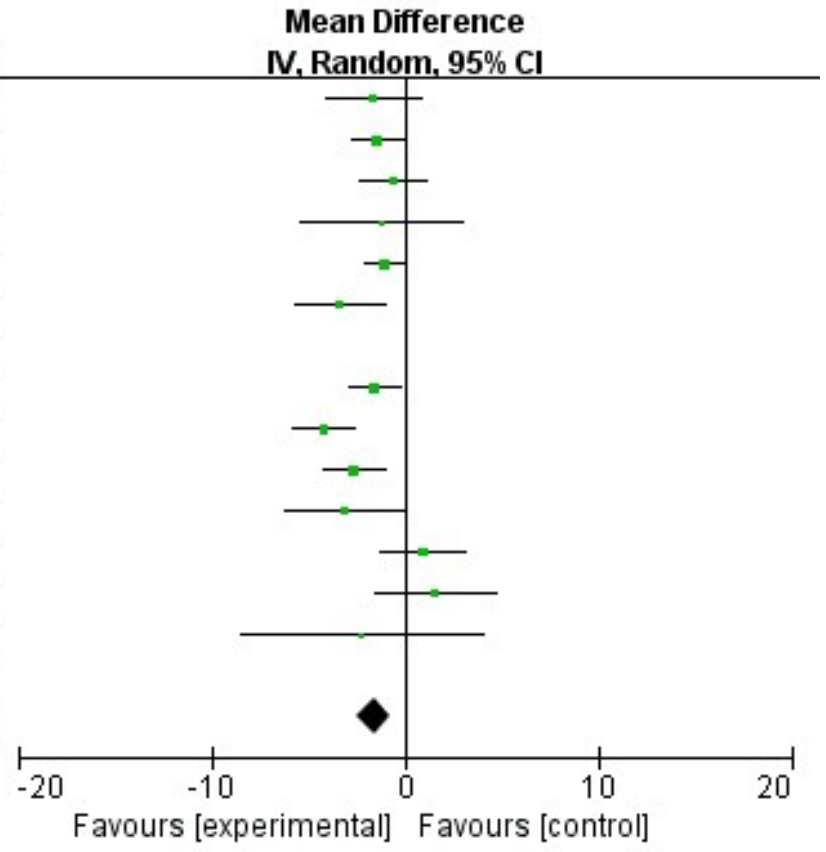

Study or Subgroup	Experimental Mean	Experimental SD	Experimental Total	Control Mean	Control SD	Control Total	Weight	Mean Difference IV, Random, 95% CI
Choi et al. 2016	-0.36	0.9	11	1.26	3.7	9	7.2%	-1.62 [-4.10, 0.86]
Gao et al. 2014	-1.37	2.93	37	0.03	2.85	39	12.1%	-1.40 [-2.70, -0.10]
Gloria VD et al. 2018	-0.58	2.62	12	0	1.78	13	9.9%	-0.58 [-2.35, 1.19]
Kwok et al. 2019	-2.82	15.39	58	-1.64	5.61	59	3.5%	-1.18 [-5.39, 3.03]
Li et al. 2012	-1.05	2.8	65	-0.02	3.32	65	13.2%	-1.03 [-2.09, 0.03]
Li et al. 2022a	-1.38	3.03	19	1.95	4.22	19	7.6%	-3.33 [-5.67, -0.99]
Li et al. 2022b	-2.49	2.26	15	-0.17	6.34	16	5.0%	-2.32 [-5.63, 0.99]
Li et al. 2022c	-1.78	2.98	32	-0.26	1.71	17	0.0%	-1.52 [-2.83, -0.21]
Liu et al. 2016	-4.27	2.41	23	-0.1	2.77	18	10.6%	-4.17 [-5.78, -2.56]
Ni et al. 2016	-2.3	2.9	13	0.3	0.84	10	10.4%	-2.60 [-4.26, -0.94]
Wan et al. 2021	-2.21	4.09	20	0.94	5.79	20	5.4%	-3.15 [-6.26, -0.04]
Wang et al. 2022	-0.42	2.01	23	-1.4	4.85	22	8.2%	0.98 [-1.21, 3.17]
Witoon M et al. 2022	1	5.02	17	-0.6	4.04	16	5.4%	1.60 [-1.50, 4.70]
Xiao et al. 2016	-1.2	11.65	45	1	17.83	44	1.8%	-2.20 [-8.47, 4.07]
**Total (95% CI)**			**358**			**350**	**100.0%**	**-1.64 [-2.52, -0.76]**

Heterogeneity: $Tau^2 = 1.24$; $Chi^2 = 26.36$, df = 12 (P = 0.010); $I^2 = 54\%$
Test for overall effect: Z = 3.64 (P = 0.0003)

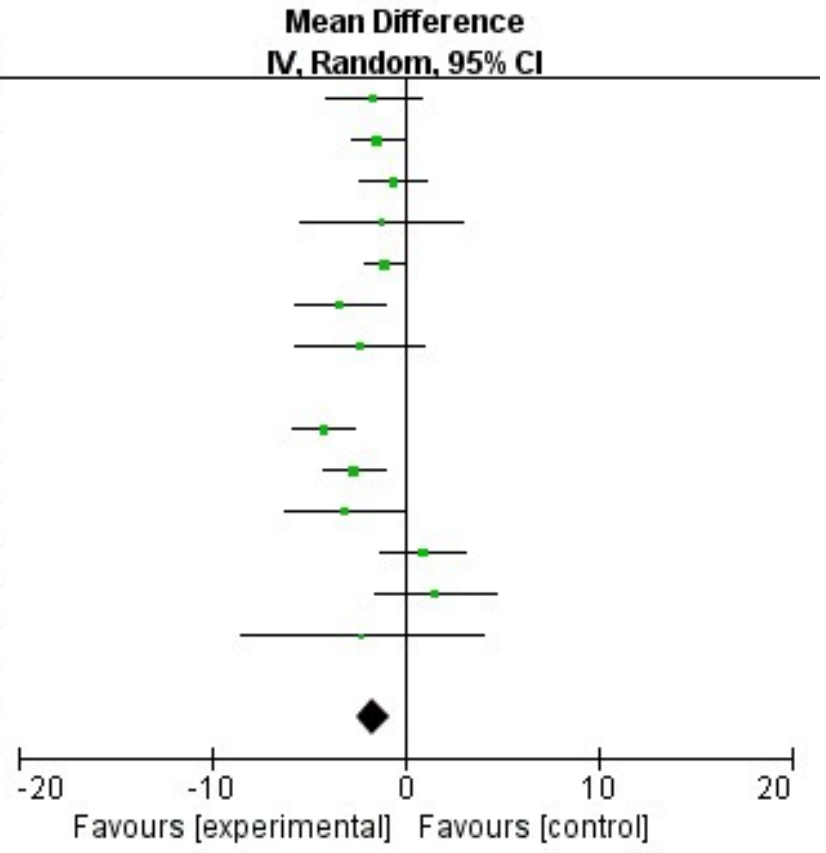

Study or Subgroup	Experimental Mean	Experimental SD	Experimental Total	Control Mean	Control SD	Control Total	Weight	Mean Difference IV, Fixed, 95% CI
Choi et al. 2016	-0.36	0.9	11	1.26	3.7	9	4.4%	-1.62 [-4.10, 0.86]
Gao et al. 2014	-1.37	2.93	37	0.03	2.85	39	16.0%	-1.40 [-2.70, -0.10]
Gloria VD et al. 2018	-0.58	2.62	12	0	1.78	13	8.7%	-0.58 [-2.35, 1.19]
Kwok et al. 2019	-2.82	15.39	58	-1.64	5.61	59	1.5%	-1.18 [-5.39, 3.03]
Li et al. 2012	-1.05	2.8	65	-0.02	3.32	65	24.3%	-1.03 [-2.09, 0.03]
Li et al. 2022a	-1.38	3.03	19	1.95	4.22	19	5.0%	-3.33 [-5.67, -0.99]
Li et al. 2022b	-2.49	2.26	15	-0.17	6.34	16	2.5%	-2.32 [-5.63, 0.99]
Li et al. 2022c	-1.78	2.98	32	-0.26	1.71	17	15.7%	-1.52 [-2.83, -0.21]
Liu et al. 2016	-4.27	2.41	23	-0.1	2.77	18	0.0%	-4.17 [-5.78, -2.56]
Ni et al. 2016	-2.3	2.9	13	0.3	0.84	10	9.8%	-2.60 [-4.26, -0.94]
Wan et al. 2021	-2.21	4.09	20	0.94	5.79	20	2.8%	-3.15 [-6.26, -0.04]
Wang et al. 2022	-0.42	2.01	23	-1.4	4.85	22	5.7%	0.98 [-1.21, 3.17]
Witoon M et al. 2022	1	5.02	17	-0.6	4.04	16	2.8%	1.60 [-1.50, 4.70]
Xiao et al. 2016	-1.2	11.65	45	1	17.83	44	0.7%	-2.20 [-8.47, 4.07]
**Total (95% CI)**			**367**			**349**	**100.0%**	**-1.34 [-1.86, -0.82]**

Heterogeneity: $Chi^2 = 15.66$, df = 12 (P = 0.21); $I^2 = 23\%$
Test for overall effect: Z = 5.03 (P < 0.00001)

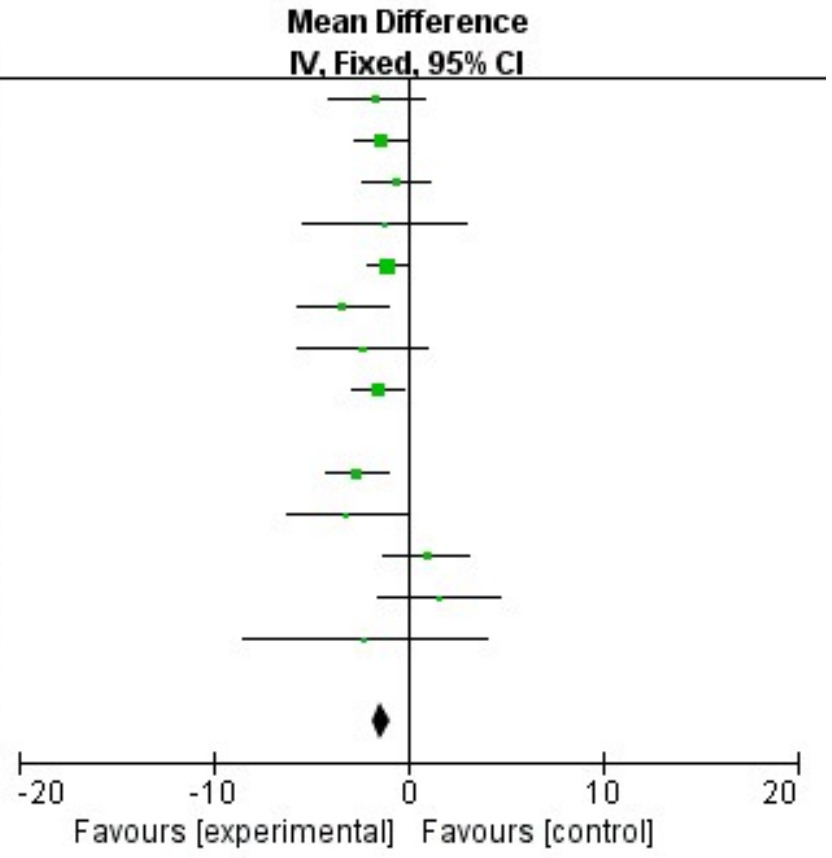

Study or Subgroup	Experimental Mean	Experimental SD	Experimental Total	Control Mean	Control SD	Control Total	Weight	Mean Difference IV, Random, 95% CI
Choi et al. 2016	-0.36	0.9	11	1.26	3.7	9	6.9%	-1.62 [-4.10, 0.86]
Gao et al. 2014	-1.37	2.93	37	0.03	2.85	39	12.2%	-1.40 [-2.70, -0.10]
Gloria VD et al. 2018	-0.58	2.62	12	0	1.78	13	9.7%	-0.58 [-2.35, 1.19]
Kwok et al. 2019	-2.82	15.39	58	-1.64	5.61	59	3.2%	-1.18 [-5.39, 3.03]
Li et al. 2012	-1.05	2.8	65	-0.02	3.32	65	13.6%	-1.03 [-2.09, 0.03]
Li et al. 2022a	-1.38	3.03	19	1.95	4.22	19	7.4%	-3.33 [-5.67, -0.99]
Li et al. 2022b	-2.49	2.26	15	-0.17	6.34	16	4.7%	-2.32 [-5.63, 0.99]
Li et al. 2022c	-1.78	2.98	32	-0.26	1.71	17	12.1%	-1.52 [-2.83, -0.21]
Liu et al. 2016	-4.27	2.41	23	-0.1	2.77	18	10.5%	-4.17 [-5.78, -2.56]
Ni et al. 2016	-2.3	2.9	13	0.3	0.84	10	0.0%	-2.60 [-4.26, -0.94]
Wan et al. 2021	-2.21	4.09	20	0.94	5.79	20	5.1%	-3.15 [-6.26, -0.04]
Wang et al. 2022	-0.42	2.01	23	-1.4	4.85	22	7.9%	0.98 [-1.21, 3.17]
Witoon M et al. 2022	1	5.02	17	-0.6	4.04	16	5.1%	1.60 [-1.50, 4.70]
Xiao et al. 2016	-1.2	11.65	45	1	17.83	44	1.6%	-2.20 [-8.47, 4.07]
**Total (95% CI)**			**377**			**357**	**100.0%**	**-1.53 [-2.36, -0.69]**

Heterogeneity: Tau² = 1.05; Chi² = 24.86, df = 12 (P = 0.02); I² = 52%
Test for overall effect: Z = 3.59 (P = 0.0003)

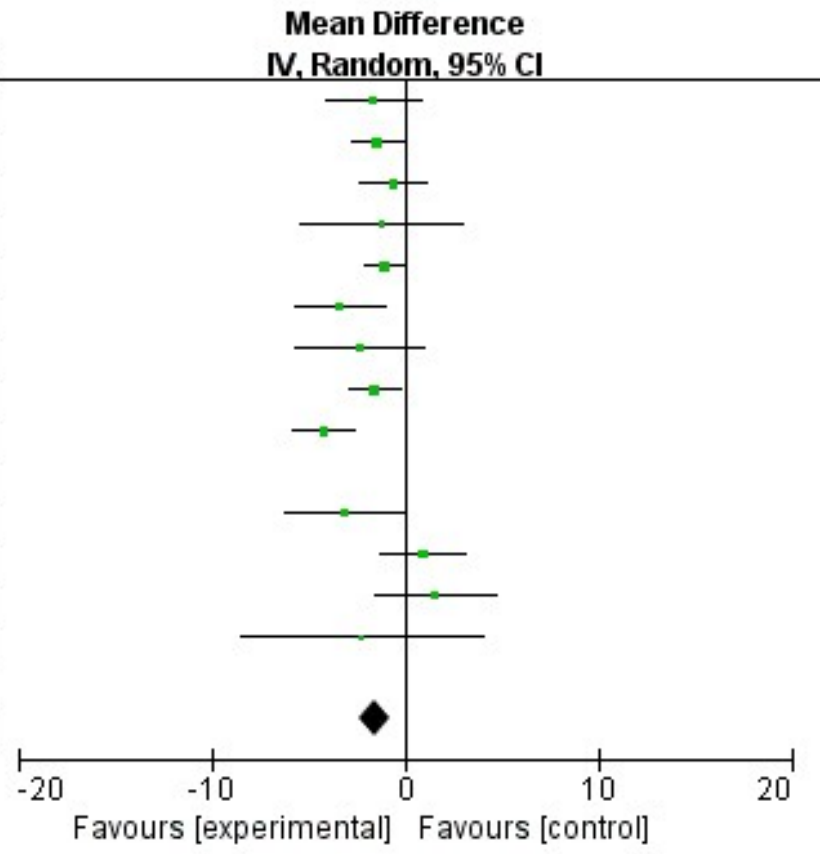

Study or Subgroup	Experimental Mean	Experimental SD	Experimental Total	Control Mean	Control SD	Control Total	Weight	Mean Difference IV, Random, 95% CI
Choi et al. 2016	-0.36	0.9	11	1.26	3.7	9	6.5%	-1.62 [-4.10, 0.86]
Gao et al. 2014	-1.37	2.93	37	0.03	2.85	39	11.7%	-1.40 [-2.70, -0.10]
Gloria VD et al. 2018	-0.58	2.62	12	0	1.78	13	9.3%	-0.58 [-2.35, 1.19]
Kwok et al. 2019	-2.82	15.39	58	-1.64	5.61	59	3.0%	-1.18 [-5.39, 3.03]
Li et al. 2012	-1.05	2.8	65	-0.02	3.32	65	13.0%	-1.03 [-2.09, 0.03]
Li et al. 2022a	-1.38	3.03	19	1.95	4.22	19	7.0%	-3.33 [-5.67, -0.99]
Li et al. 2022b	-2.49	2.26	15	-0.17	6.34	16	4.4%	-2.32 [-5.63, 0.99]
Li et al. 2022c	-1.78	2.98	32	-0.26	1.71	17	11.6%	-1.52 [-2.83, -0.21]
Liu et al. 2016	-4.27	2.41	23	-0.1	2.77	18	10.0%	-4.17 [-5.78, -2.56]
Ni et al. 2016	-2.3	2.9	13	0.3	0.84	10	9.8%	-2.60 [-4.26, -0.94]
Wan et al. 2021	-2.21	4.09	20	0.94	5.79	20	0.0%	-3.15 [-6.26, -0.04]
Wang et al. 2022	-0.42	2.01	23	-1.4	4.85	22	7.5%	0.98 [-1.21, 3.17]
Witoon M et al. 2022	1	5.02	17	-0.6	4.04	16	4.8%	1.60 [-1.50, 4.70]
Xiao et al. 2016	-1.2	11.65	45	1	17.83	44	1.5%	-2.20 [-8.47, 4.07]
**Total (95% CI)**			**370**			**347**	**100.0%**	**-1.56 [-2.36, -0.75]**

Heterogeneity: Tau² = 1.01; Chi² = 25.40, df = 12 (P = 0.01); I² = 53%
Test for overall effect: Z = 3.79 (P = 0.0002)

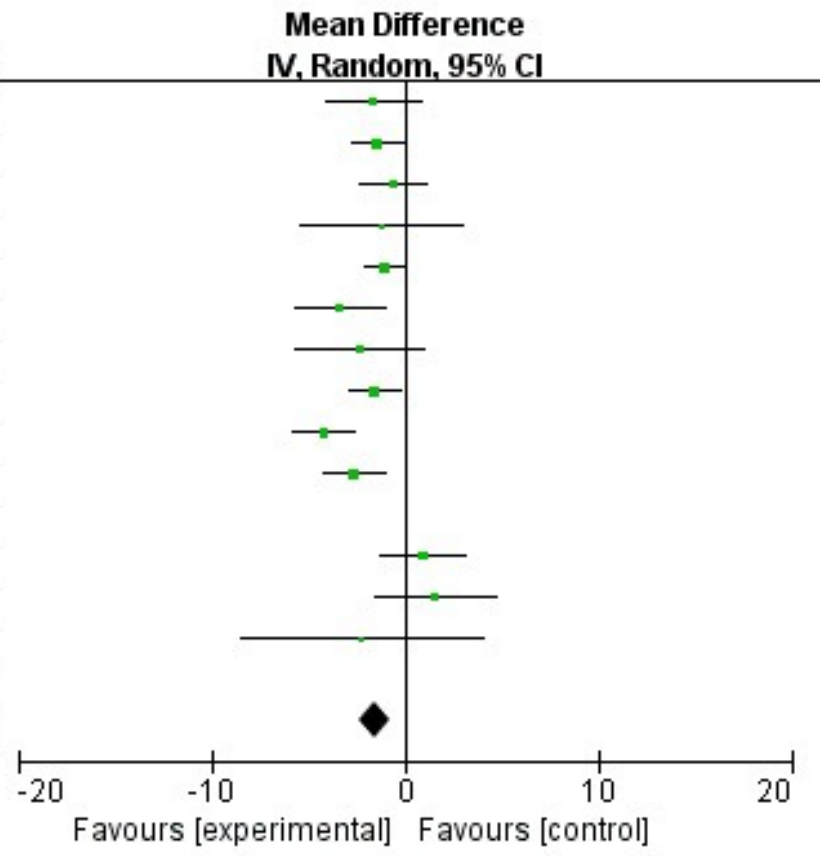

Study or Subgroup	Experimental Mean	Experimental SD	Experimental Total	Control Mean	Control SD	Control Total	Weight	Mean Difference IV, Fixed, 95% CI
Choi et al. 2016	-0.36	0.9	11	1.26	3.7	9	4.2%	-1.62 [-4.10, 0.86]
Gao et al. 2014	-1.37	2.93	37	0.03	2.85	39	15.3%	-1.40 [-2.70, -0.10]
Gloria VD et al. 2018	-0.58	2.62	12	0	1.78	13	8.3%	-0.58 [-2.35, 1.19]
Kwok et al. 2019	-2.82	15.39	58	-1.64	5.61	59	1.5%	-1.18 [-5.39, 3.03]
Li et al. 2012	-1.05	2.8	65	-0.02	3.32	65	23.2%	-1.03 [-2.09, 0.03]
Li et al. 2022a	-1.38	3.03	19	1.95	4.22	19	4.7%	-3.33 [-5.67, -0.99]
Li et al. 2022b	-2.49	2.26	15	-0.17	6.34	16	2.4%	-2.32 [-5.63, 0.99]
Li et al. 2022c	-1.78	2.98	32	-0.26	1.71	17	15.0%	-1.52 [-2.83, -0.21]
Liu et al. 2016	-4.27	2.41	23	-0.1	2.77	18	9.9%	-4.17 [-5.78, -2.56]
Ni et al. 2016	-2.3	2.9	13	0.3	0.84	10	9.4%	-2.60 [-4.26, -0.94]
Wan et al. 2021	-2.21	4.09	20	0.94	5.79	20	2.7%	-3.15 [-6.26, -0.04]
Wang et al. 2022	-0.42	2.01	23	-1.4	4.85	22	0.0%	0.98 [-1.21, 3.17]
Witoon M et al. 2022	1	5.02	17	-0.6	4.04	16	2.7%	1.60 [-1.50, 4.70]
Xiao et al. 2016	-1.2	11.65	45	1	17.83	44	0.7%	-2.20 [-8.47, 4.07]
**Total (95% CI)**			**367**			**345**	**100.0%**	**-1.74 [-2.25, -1.23]**

Heterogeneity: Chi² = 20.73, df = 12 (P = 0.05); I² = 42%
Test for overall effect: Z = 6.71 (P < 0.00001)

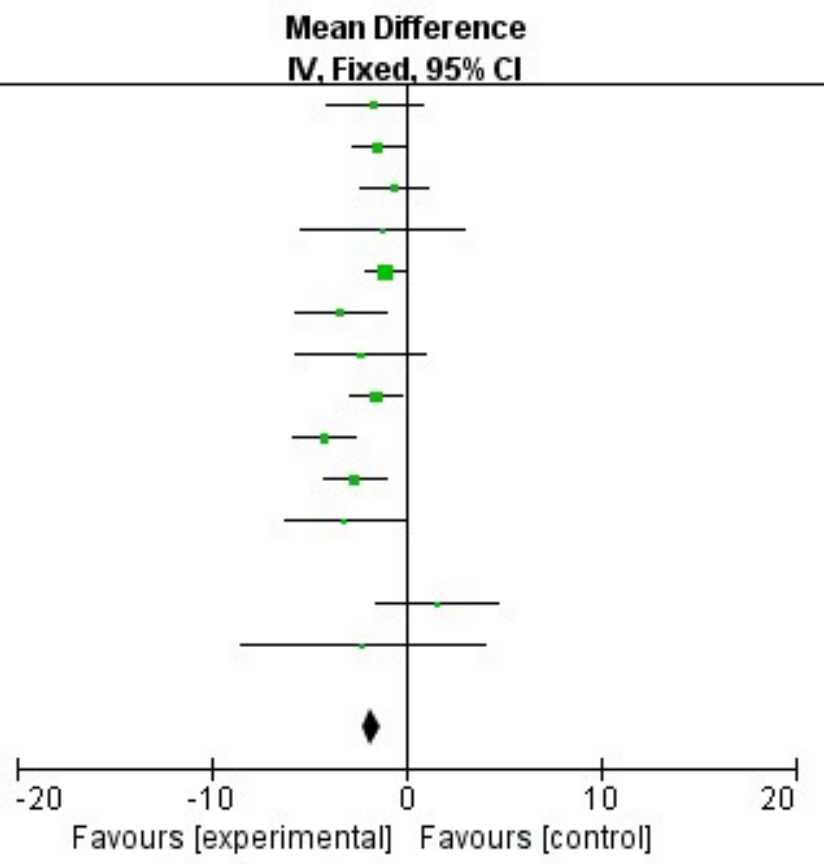

Study or Subgroup	Experimental Mean	Experimental SD	Experimental Total	Control Mean	Control SD	Control Total	Weight	Mean Difference IV, Fixed, 95% CI
Choi et al. 2016	-0.36	0.9	11	1.26	3.7	9	4.1%	-1.62 [-4.10, 0.86]
Gao et al. 2014	-1.37	2.93	37	0.03	2.85	39	14.9%	-1.40 [-2.70, -0.10]
Gloria VD et al. 2018	-0.58	2.62	12	0	1.78	13	8.0%	-0.58 [-2.35, 1.19]
Kwok et al. 2019	-2.82	15.39	58	-1.64	5.61	59	1.4%	-1.18 [-5.39, 3.03]
Li et al. 2012	-1.05	2.8	65	-0.02	3.32	65	22.6%	-1.03 [-2.09, 0.03]
Li et al. 2022a	-1.38	3.03	19	1.95	4.22	19	4.6%	-3.33 [-5.67, -0.99]
Li et al. 2022b	-2.49	2.26	15	-0.17	6.34	16	2.3%	-2.32 [-5.63, 0.99]
Li et al. 2022c	-1.78	2.98	32	-0.26	1.71	17	14.6%	-1.52 [-2.83, -0.21]
Liu et al. 2016	-4.27	2.41	23	-0.1	2.77	18	9.7%	-4.17 [-5.78, -2.56]
Ni et al. 2016	-2.3	2.9	13	0.3	0.84	10	9.2%	-2.60 [-4.26, -0.94]
Wan et al. 2021	-2.21	4.09	20	0.94	5.79	20	2.6%	-3.15 [-6.26, -0.04]
Wang et al. 2022	-0.42	2.01	23	-1.4	4.85	22	5.3%	0.98 [-1.21, 3.17]
Witoon M et al. 2022	1	5.02	17	-0.6	4.04	16	0.0%	1.60 [-1.50, 4.70]
Xiao et al. 2016	-1.2	11.65	45	1	17.83	44	0.6%	-2.20 [-8.47, 4.07]
**Total (95% CI)**			**373**			**351**	**100.0%**	**-1.69 [-2.19, -1.18]**

Heterogeneity: Chi² = 22.17, df = 12 (P = 0.04); I² = 46%
Test for overall effect: Z = 6.58 (P < 0.00001)

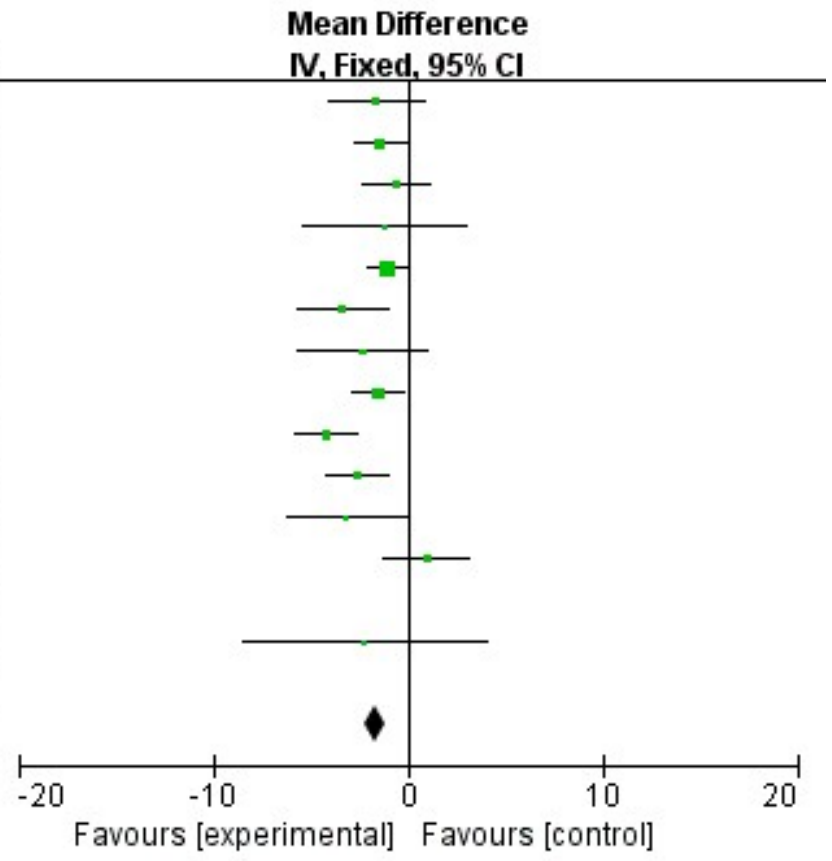

Study or Subgroup	Experimental Mean	Experimental SD	Experimental Total	Control Mean	Control SD	Control Total	Weight	Mean Difference IV, Random, 95% CI
Choi et al. 2016	-0.36	0.9	11	1.26	3.7	9	6.3%	-1.62 [-4.10, 0.86]
Gao et al. 2014	-1.37	2.93	37	0.03	2.85	39	11.2%	-1.40 [-2.70, -0.10]
Gloria VD et al. 2018	-0.58	2.62	12	0	1.78	13	9.0%	-0.58 [-2.35, 1.19]
Kwok et al. 2019	-2.82	15.39	58	-1.64	5.61	59	3.0%	-1.18 [-5.39, 3.03]
Li et al. 2012	-1.05	2.8	65	-0.02	3.32	65	12.5%	-1.03 [-2.09, 0.03]
Li et al. 2022a	-1.38	3.03	19	1.95	4.22	19	6.8%	-3.33 [-5.67, -0.99]
Li et al. 2022b	-2.49	2.26	15	-0.17	6.34	16	4.3%	-2.32 [-5.63, 0.99]
Li et al. 2022c	-1.78	2.98	32	-0.26	1.71	17	11.2%	-1.52 [-2.83, -0.21]
Liu et al. 2016	-4.27	2.41	23	-0.1	2.77	18	9.7%	-4.17 [-5.78, -2.56]
Ni et al. 2016	-2.3	2.9	13	0.3	0.84	10	9.5%	-2.60 [-4.26, -0.94]
Wan et al. 2021	-2.21	4.09	20	0.94	5.79	20	4.7%	-3.15 [-6.26, -0.04]
Wang et al. 2022	-0.42	2.01	23	-1.4	4.85	22	7.3%	0.98 [-1.21, 3.17]
Witoon M et al. 2022	1	5.02	17	-0.6	4.04	16	4.7%	1.60 [-1.50, 4.70]
Xiao et al. 2016	-1.2	11.65	45	1	17.83	44	0.0%	-2.20 [-8.47, 4.07]
**Total (95% CI)**			**345**			**323**	**100.0%**	**-1.62 [-2.42, -0.82]**

Heterogeneity: Tau² = 1.05; Chi² = 26.35, df = 12 (P = 0.010); I² = 54%
Test for overall effect: Z = 3.96 (P < 0.0001)

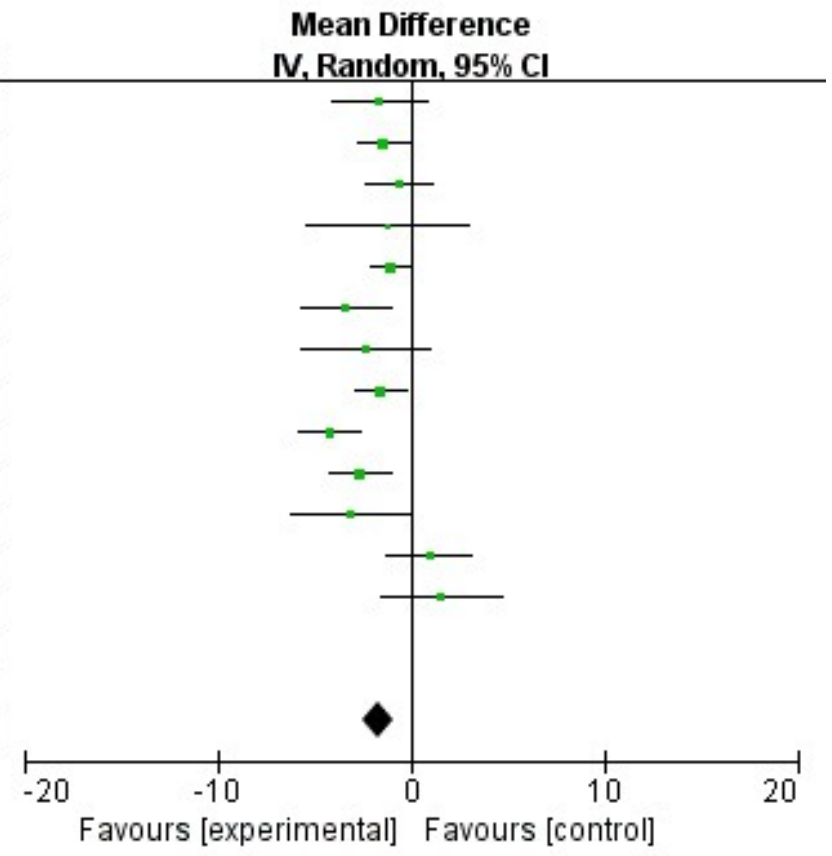

eFigure 3. shows the sensitivity analysis of BBS

Study or Subgroup	Experimental Mean	Experimental SD	Experimental Total	Control Mean	Control SD	Control Total	Weight	Mean Difference IV, Random, 95% CI
Gao et al. 2014	3.83	8.74	37	0.06	9.9	39	0.0%	3.77 [-0.42, 7.96]
Lee et al. 2018	0.9	2.45	25	-0.4	3.35	16	21.6%	1.30 [-0.60, 3.20]
Li et al. 2022c	3.9	3.16	32	-0.4	3.26	17	21.7%	4.30 [2.40, 6.20]
Lin et al. 2023	2.9	2.18	15	1.5	1.84	15	26.1%	1.40 [-0.04, 2.84]
Ni et al. 2016	4.2	2.9	13	0.4	0.7	10	24.2%	3.80 [2.16, 5.44]
Xiao et al. 2016	2.5	9.7	45	-1.2	13.85	44	6.4%	3.70 [-1.28, 8.68]
**Total (95% CI)**			**130**			**102**	**100.0%**	**2.73 [1.34, 4.13]**

Heterogeneity: Tau² = 1.39; Chi² = 9.79, df = 4 (P = 0.04); I² = 59%
Test for overall effect: Z = 3.85 (P = 0.0001)

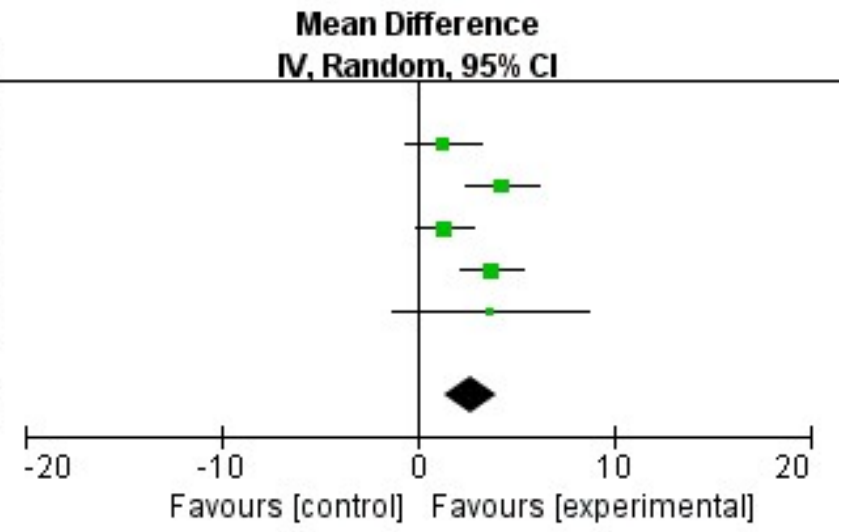

Study or Subgroup	Experimental Mean	Experimental SD	Experimental Total	Control Mean	Control SD	Control Total	Weight	Mean Difference IV, Fixed, 95% CI
Gao et al. 2014	3.83	8.74	37	0.06	9.9	39	4.6%	3.77 [-0.42, 7.96]
Lee et al. 2018	0.9	2.45	25	-0.4	3.35	16	0.0%	1.30 [-0.60, 3.20]
Li et al. 2022c	3.9	3.16	32	-0.4	3.26	17	22.6%	4.30 [2.40, 6.20]
Lin et al. 2023	2.9	2.18	15	1.5	1.84	15	39.0%	1.40 [-0.04, 2.84]
Ni et al. 2016	4.2	2.9	13	0.4	0.7	10	30.4%	3.80 [2.16, 5.44]
Xiao et al. 2016	2.5	9.7	45	-1.2	13.85	44	3.3%	3.70 [-1.28, 8.68]
**Total (95% CI)**			**142**			**125**	**100.0%**	**2.97 [2.07, 3.87]**

Heterogeneity: Chi² = 7.64, df = 4 (P = 0.11); I² = 48%
Test for overall effect: Z = 6.46 (P < 0.00001)

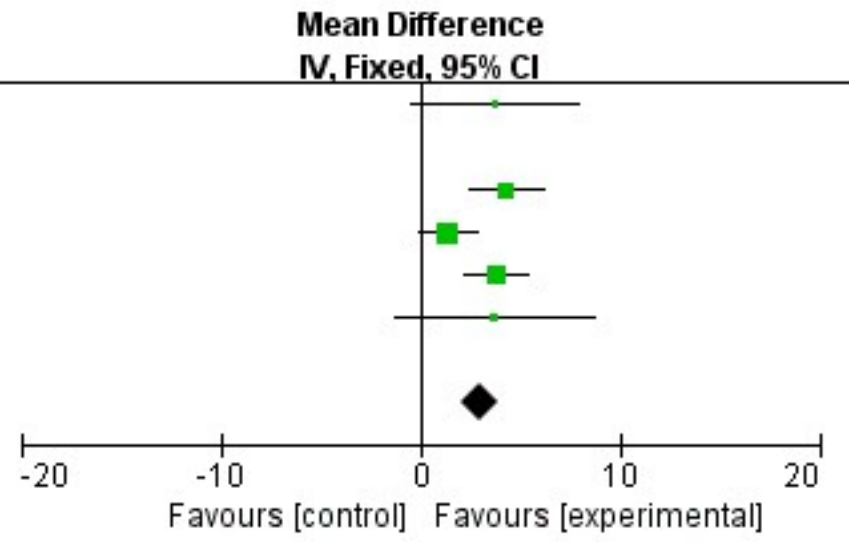

Study or Subgroup	Experimental Mean	Experimental SD	Experimental Total	Control Mean	Control SD	Control Total	Weight	Mean Difference IV, Fixed, 95% CI
Gao et al. 2014	3.83	8.74	37	0.06	9.9	39	4.6%	3.77 [-0.42, 7.96]
Lee et al. 2018	0.9	2.45	25	-0.4	3.35	16	22.5%	1.30 [-0.60, 3.20]
Li et al. 2022c	3.9	3.16	32	-0.4	3.26	17	0.0%	4.30 [2.40, 6.20]
Lin et al. 2023	2.9	2.18	15	1.5	1.84	15	39.1%	1.40 [-0.04, 2.84]
Ni et al. 2016	4.2	2.9	13	0.4	0.7	10	30.5%	3.80 [2.16, 5.44]
Xiao et al. 2016	2.5	9.7	45	-1.2	13.85	44	3.3%	3.70 [-1.28, 8.68]
**Total (95% CI)**			**135**			**124**	**100.0%**	**2.29 [1.39, 3.20]**

Heterogeneity: Chi² = 6.56, df = 4 (P = 0.16); I² = 39%
Test for overall effect: Z = 4.98 (P < 0.00001)

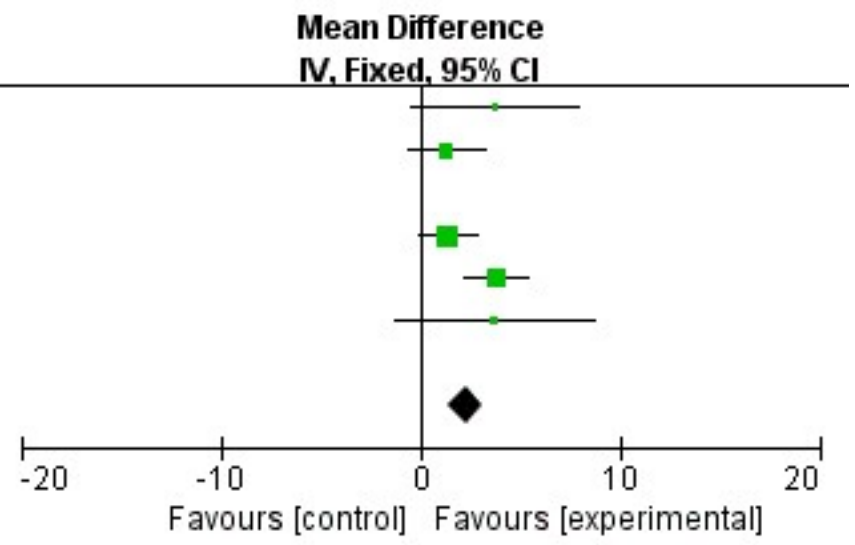

Study or Subgroup	Experimental Mean	Experimental SD	Experimental Total	Control Mean	Control SD	Control Total	Weight	Mean Difference IV, Fixed, 95% CI
Gao et al. 2014	3.83	8.74	37	0.06	9.9	39	5.5%	3.77 [-0.42, 7.96]
Lee et al. 2018	0.9	2.45	25	-0.4	3.35	16	27.0%	1.30 [-0.60, 3.20]
Li et al. 2022c	3.9	3.16	32	-0.4	3.26	17	27.1%	4.30 [2.40, 6.20]
Lin et al. 2023	2.9	2.18	15	1.5	1.84	15	0.0%	1.40 [-0.04, 2.84]
Ni et al. 2016	4.2	2.9	13	0.4	0.7	10	36.5%	3.80 [2.16, 5.44]
Xiao et al. 2016	2.5	9.7	45	-1.2	13.85	44	3.9%	3.70 [-1.28, 8.68]
**Total (95% CI)**			**152**			**126**	**100.0%**	**3.26 [2.27, 4.24]**

Heterogeneity: Chi² = 5.74, df = 4 (P = 0.22); I² = 30%
Test for overall effect: Z = 6.46 (P < 0.00001)

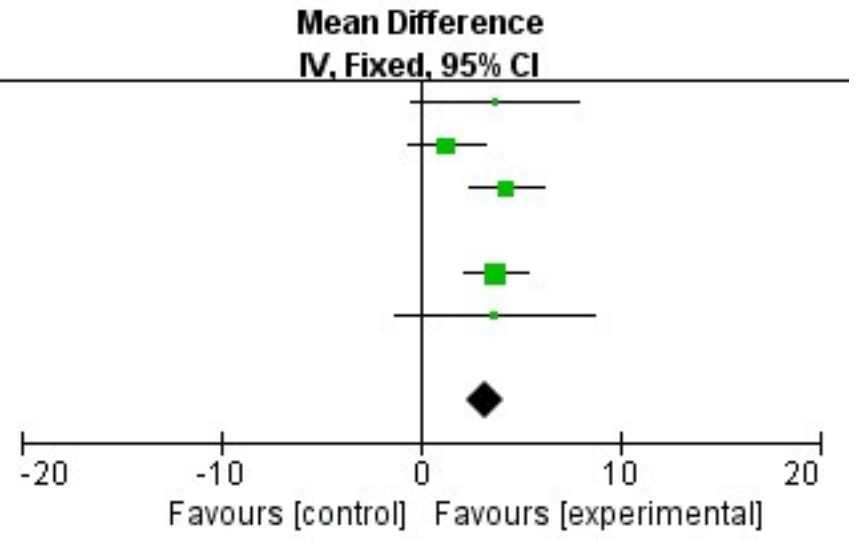

Study or Subgroup	Experimental Mean	Experimental SD	Experimental Total	Control Mean	Control SD	Control Total	Weight	Mean Difference IV, Fixed, 95% CI
Gao et al. 2014	3.83	8.74	37	0.06	9.9	39	5.0%	3.77 [-0.42, 7.96]
Lee et al. 2018	0.9	2.45	25	-0.4	3.35	16	24.4%	1.30 [-0.60, 3.20]
Li et al. 2022c	3.9	3.16	32	-0.4	3.26	17	24.6%	4.30 [2.40, 6.20]
Lin et al. 2023	2.9	2.18	15	1.5	1.84	15	42.4%	1.40 [-0.04, 2.84]
Ni et al. 2016	4.2	2.9	13	0.4	0.7	10	0.0%	3.80 [2.16, 5.44]
Xiao et al. 2016	2.5	9.7	45	-1.2	13.85	44	3.6%	3.70 [-1.28, 8.68]
**Total (95% CI)**			**154**			**131**	**100.0%**	**2.29 [1.35, 3.23]**

Heterogeneity: Chi² = 7.60, df = 4 (P = 0.11); I² = 47%
Test for overall effect: Z = 4.77 (P < 0.00001)

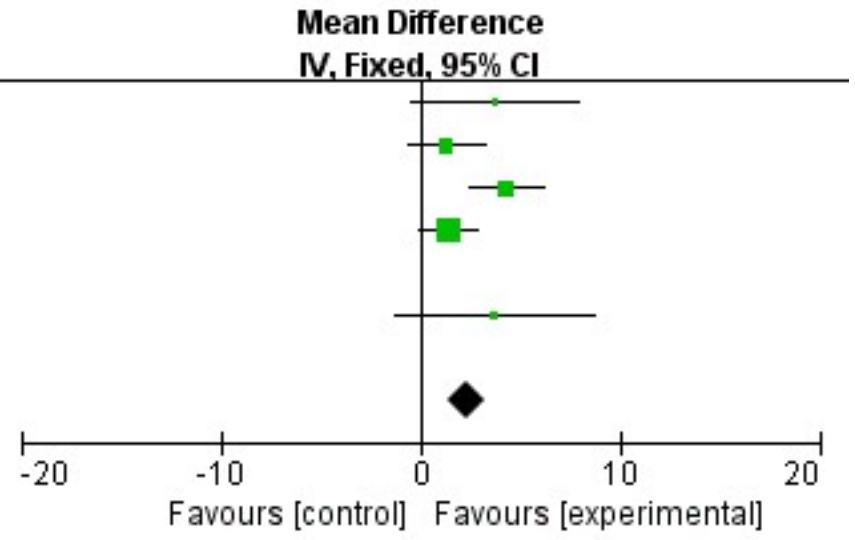

Study or Subgroup	Experimental Mean	Experimental SD	Experimental Total	Control Mean	Control SD	Control Total	Weight	Mean Difference IV, Random, 95% CI
Gao et al. 2014	3.83	8.74	37	0.06	9.9	39	8.3%	3.77 [-0.42, 7.96]
Lee et al. 2018	0.9	2.45	25	-0.4	3.35	16	21.2%	1.30 [-0.60, 3.20]
Li et al. 2022c	3.9	3.16	32	-0.4	3.26	17	21.2%	4.30 [2.40, 6.20]
Lin et al. 2023	2.9	2.18	15	1.5	1.84	15	25.6%	1.40 [-0.04, 2.84]
Ni et al. 2016	4.2	2.9	13	0.4	0.7	10	23.7%	3.80 [2.16, 5.44]
Xiao et al. 2016	2.5	9.7	45	-1.2	13.85	44	0.0%	3.70 [-1.28, 8.68]
**Total (95% CI)**			**122**			**97**	**100.0%**	**2.76 [1.38, 4.14]**

Heterogeneity: Tau² = 1.39; Chi² = 9.89, df = 4 (P = 0.04); I² = 60%
Test for overall effect: Z = 3.93 (P < 0.0001)

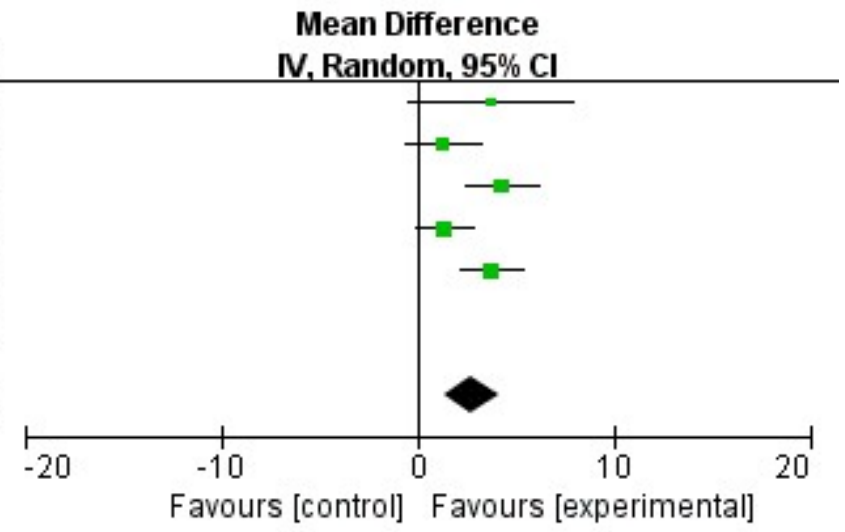

eFigure 4. shows the sensitivity analysis of MoCA

Study or Subgroup	Experimental Mean	Experimental SD	Experimental Total	Control Mean	Control SD	Control Total	Weight	Mean Difference IV, Fixed, 95% CI
Huang et al. 2019	1.77	5.53	36	-1.16	4.64	38	0.0%	2.93 [0.60, 5.26]
Lin et al. 2023	2.1	2.6	15	-0.2	2.1	15	26.1%	2.30 [0.61, 3.99]
Son et al. 2018	2.99	2.91	33	-0.04	5.13	30	17.1%	3.03 [0.94, 5.12]
Wang et al. 2022	0.73	1.89	23	-0.45	2.03	22	56.7%	1.18 [0.03, 2.33]
**Total (95% CI)**			**71**			**67**	**100.0%**	**1.79 [0.93, 2.65]**

Heterogeneity: Chi² = 2.79, df = 2 (P = 0.25); I² = 28%
Test for overall effect: Z = 4.06 (P < 0.0001)

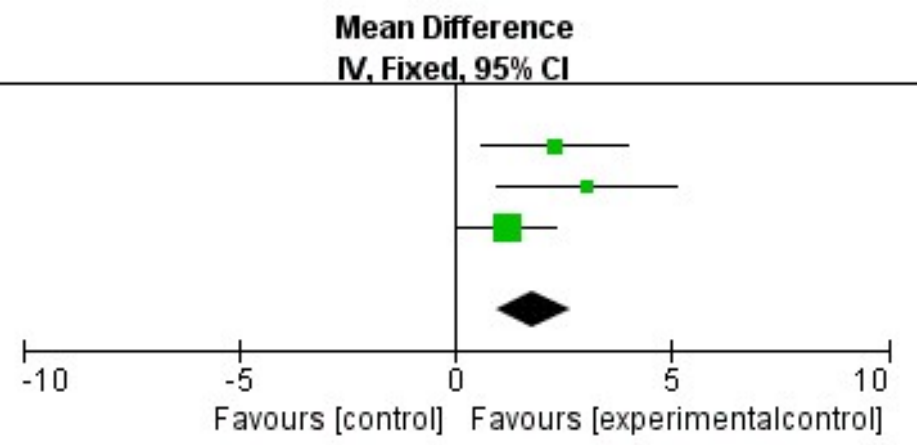

Study or Subgroup	Experimental Mean	Experimental SD	Experimental Total	Control Mean	Control SD	Control Total	Weight	Mean Difference IV, Fixed, 95% CI
Huang et al. 2019	1.77	5.53	36	-1.16	4.64	38	15.7%	2.93 [0.60, 5.26]
Lin et al. 2023	2.1	2.6	15	-0.2	2.1	15	0.0%	2.30 [0.61, 3.99]
Son et al. 2018	2.99	2.91	33	-0.04	5.13	30	19.6%	3.03 [0.94, 5.12]
Wang et al. 2022	0.73	1.89	23	-0.45	2.03	22	64.8%	1.18 [0.03, 2.33]
**Total (95% CI)**			**92**			**90**	**100.0%**	**1.82 [0.89, 2.74]**

Heterogeneity: Chi² = 3.36, df = 2 (P = 0.19); I² = 40%
Test for overall effect: Z = 3.86 (P = 0.0001)

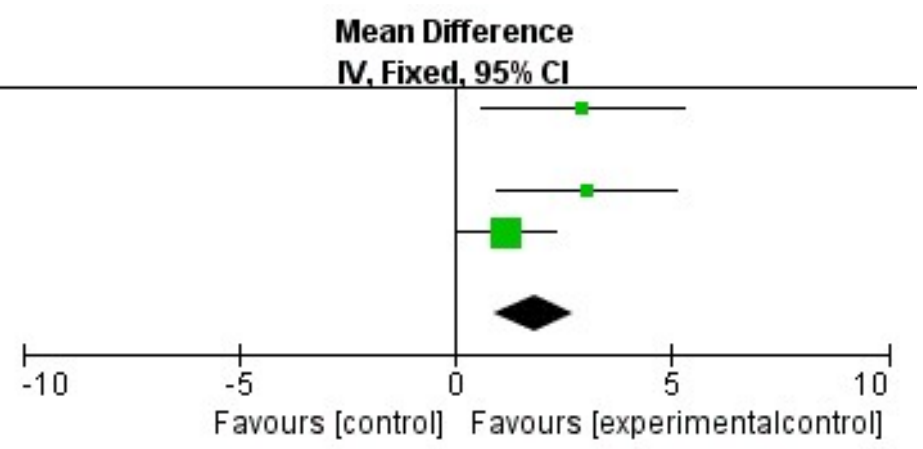

Study or Subgroup	Experimental Mean	Experimental SD	Experimental Total	Control Mean	Control SD	Control Total	Weight	Mean Difference IV, Fixed, 95% CI
Huang et al. 2019	1.77	5.53	36	-1.16	4.64	38	14.2%	2.93 [0.60, 5.26]
Lin et al. 2023	2.1	2.6	15	-0.2	2.1	15	27.0%	2.30 [0.61, 3.99]
Son et al. 2018	2.99	2.91	33	-0.04	5.13	30	0.0%	3.03 [0.94, 5.12]
Wang et al. 2022	0.73	1.89	23	-0.45	2.03	22	58.8%	1.18 [0.03, 2.33]
**Total (95% CI)**			**74**			**75**	**100.0%**	**1.73 [0.85, 2.61]**

Heterogeneity: Chi² = 2.34, df = 2 (P = 0.31); I² = 14%
Test for overall effect: Z = 3.86 (P = 0.0001)

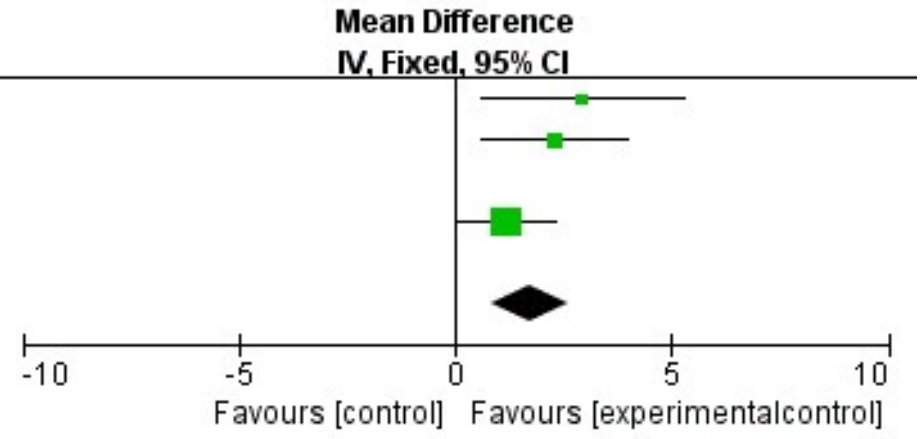

Study or Subgroup	Experimental Mean	Experimental SD	Experimental Total	Control Mean	Control SD	Control Total	Weight	Mean Difference IV, Fixed, 95% CI
Huang et al. 2019	1.77	5.53	36	-1.16	4.64	38	24.1%	2.93 [0.60, 5.26]
Lin et al. 2023	2.1	2.6	15	-0.2	2.1	15	45.8%	2.30 [0.61, 3.99]
Son et al. 2018	2.99	2.91	33	-0.04	5.13	30	30.1%	3.03 [0.94, 5.12]
Wang et al. 2022	0.73	1.89	23	-0.45	2.03	22	0.0%	1.18 [0.03, 2.33]
**Total (95% CI)**			**84**			**83**	**100.0%**	**2.67 [1.53, 3.82]**

Heterogeneity: Chi² = 0.35, df = 2 (P = 0.84); I² = 0%
Test for overall effect: Z = 4.57 (P < 0.00001)

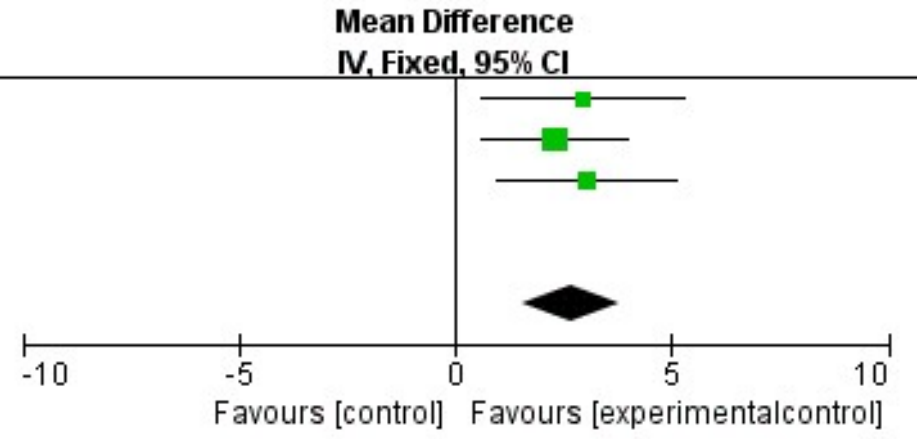

eFigure 5. shows the sensitivity analysis of MMSE

Study or Subgroup	Experimental Mean	Experimental SD	Experimental Total	Control Mean	Control SD	Control Total	Weight	Mean Difference IV, Fixed, 95% CI
Domingo J et al. 2016	-1.6	3.42	41	-7.48	4.43	29	0.0%	5.88 [3.96, 7.80]
Huang et al. 2019	0.45	6.09	36	-1.32	5.47	38	26.2%	1.77 [-0.87, 4.41]
Lin et al. 2019	1.45	7.49	11	0.9	3.64	10	7.4%	0.55 [-4.42, 5.52]
Lin et al. 2023	1.4	2.95	15	-0.4	1.43	15	66.4%	1.80 [0.14, 3.46]
**Total (95% CI)**			**62**			**63**	**100.0%**	**1.70 [0.35, 3.05]**

Heterogeneity: Chi² = 0.22, df = 2 (P = 0.89); I² = 0%
Test for overall effect: Z = 2.46 (P = 0.01)

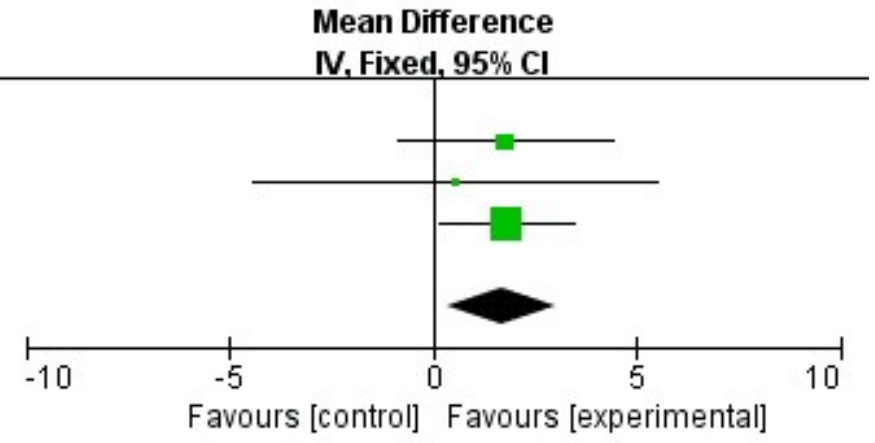

Study or Subgroup	Experimental Mean	Experimental SD	Experimental Total	Control Mean	Control SD	Control Total	Weight	Mean Difference IV, Random, 95% CI
Domingo J et al. 2016	-1.6	3.42	41	-7.48	4.43	29	38.3%	5.88 [3.96, 7.80]
Huang et al. 2019	0.45	6.09	36	-1.32	5.47	38	0.0%	1.77 [-0.87, 4.41]
Lin et al. 2019	1.45	7.49	11	0.9	3.64	10	22.1%	0.55 [-4.42, 5.52]
Lin et al. 2023	1.4	2.95	15	-0.4	1.43	15	39.6%	1.80 [0.14, 3.46]
**Total (95% CI)**			**67**			**54**	**100.0%**	**3.09 [-0.23, 6.40]**

Heterogeneity: Tau² = 6.53; Chi² = 11.23, df = 2 (P = 0.004); I² = 82%
Test for overall effect: Z = 1.82 (P = 0.07)

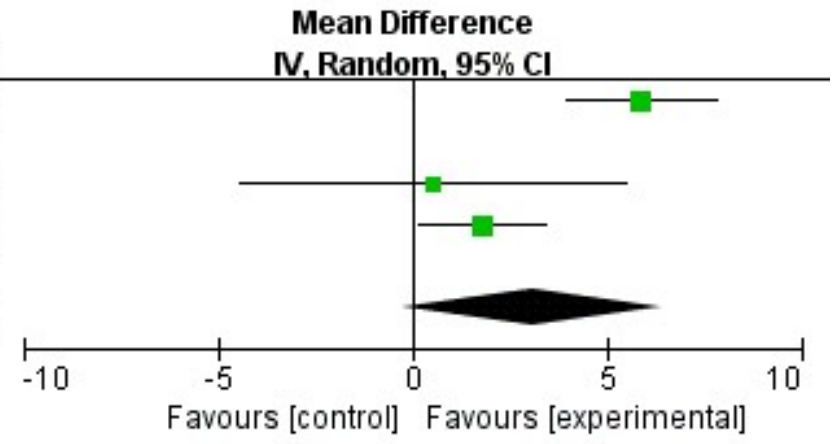

Study or Subgroup	Experimental Mean	Experimental SD	Experimental Total	Control Mean	Control SD	Control Total	Weight	Mean Difference IV, Random, 95% CI
Domingo J et al. 2016	-1.6	3.42	41	-7.48	4.43	29	40.3%	5.88 [3.96, 7.80]
Huang et al. 2019	0.45	6.09	36	-1.32	5.47	38	36.2%	1.77 [-0.87, 4.41]
Lin et al. 2019	1.45	7.49	11	0.9	3.64	10	23.5%	0.55 [-4.42, 5.52]
Lin et al. 2023	1.4	2.95	15	-0.4	1.43	15	0.0%	1.80 [0.14, 3.46]
**Total (95% CI)**			**88**			**77**	**100.0%**	**3.14 [-0.29, 6.57]**

Heterogeneity: Tau² = 6.65; Chi² = 8.24, df = 2 (P = 0.02); I² = 76%
Test for overall effect: Z = 1.79 (P = 0.07)

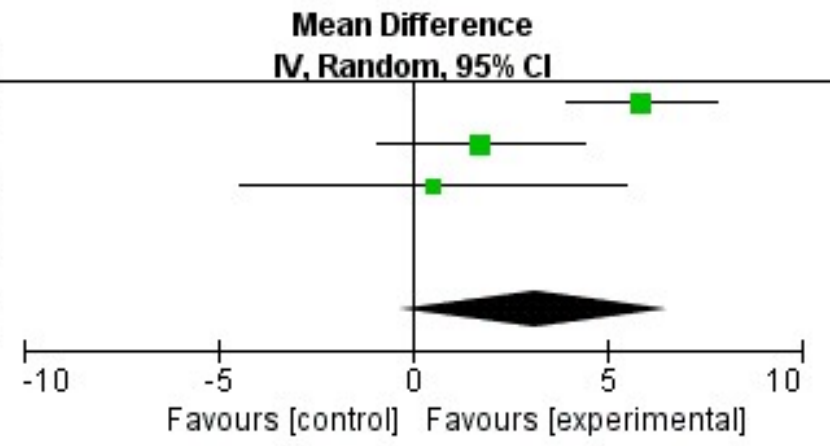

Study or Subgroup	Experimental Mean	Experimental SD	Experimental Total	Control Mean	Control SD	Control Total	Weight	Mean Difference IV, Random, 95% CI
Domingo J et al. 2016	-1.6	3.42	41	-7.48	4.43	29	34.3%	5.88 [3.96, 7.80]
Huang et al. 2019	0.45	6.09	36	-1.32	5.47	38	30.0%	1.77 [-0.87, 4.41]
Lin et al. 2019	1.45	7.49	11	0.9	3.64	10	0.0%	0.55 [-4.42, 5.52]
Lin et al. 2023	1.4	2.95	15	-0.4	1.43	15	35.8%	1.80 [0.14, 3.46]
**Total (95% CI)**			**92**			**82**	**100.0%**	**3.19 [0.39, 5.99]**

Heterogeneity: Tau² = 4.99; Chi² = 11.33, df = 2 (P = 0.003); I² = 82%
Test for overall effect: Z = 2.23 (P = 0.03)

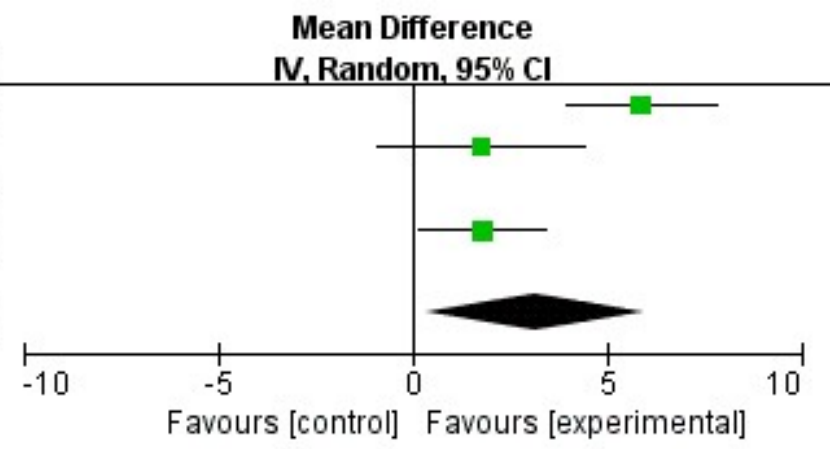

eFigure 6. shows the sensitivity analysis of BDI

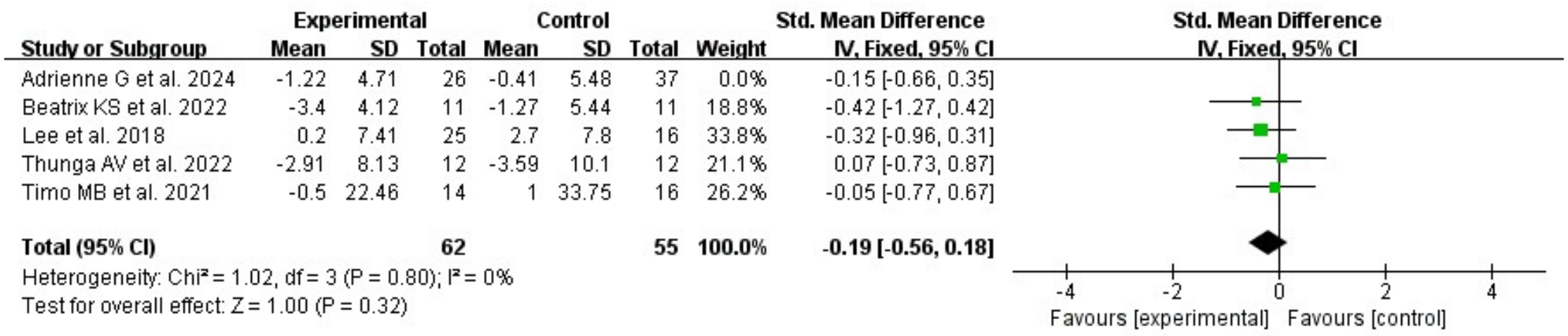

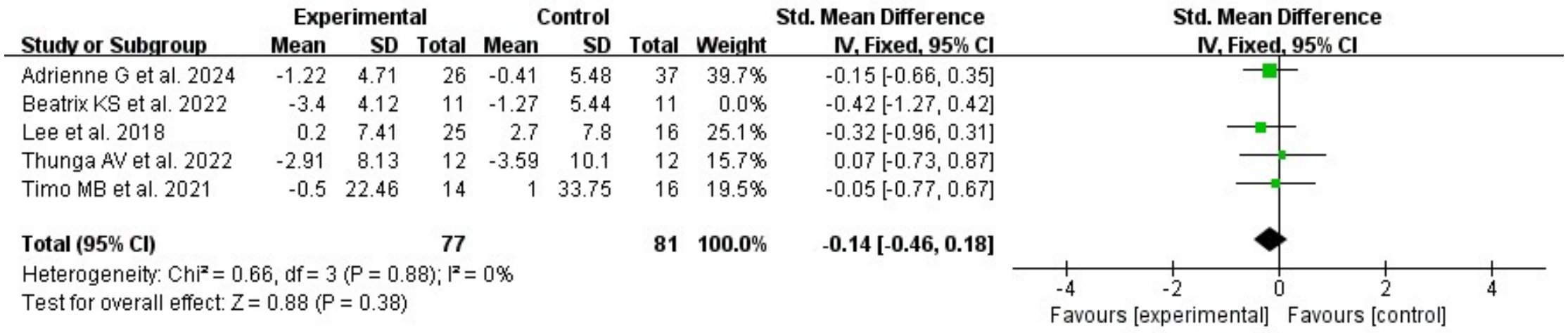

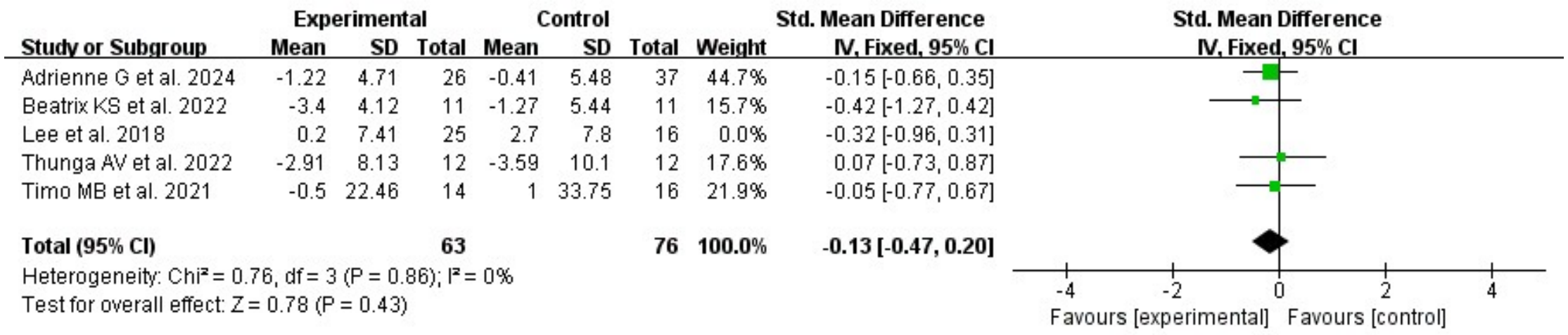

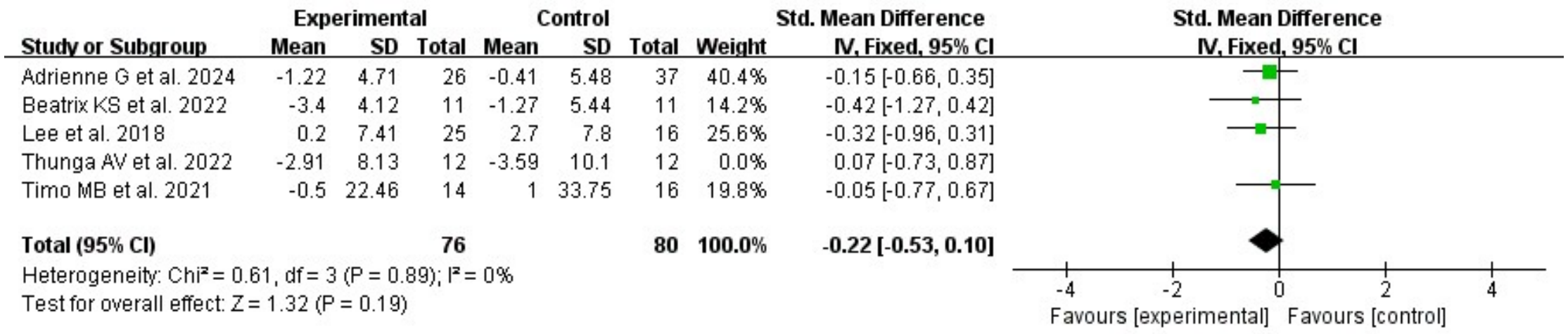

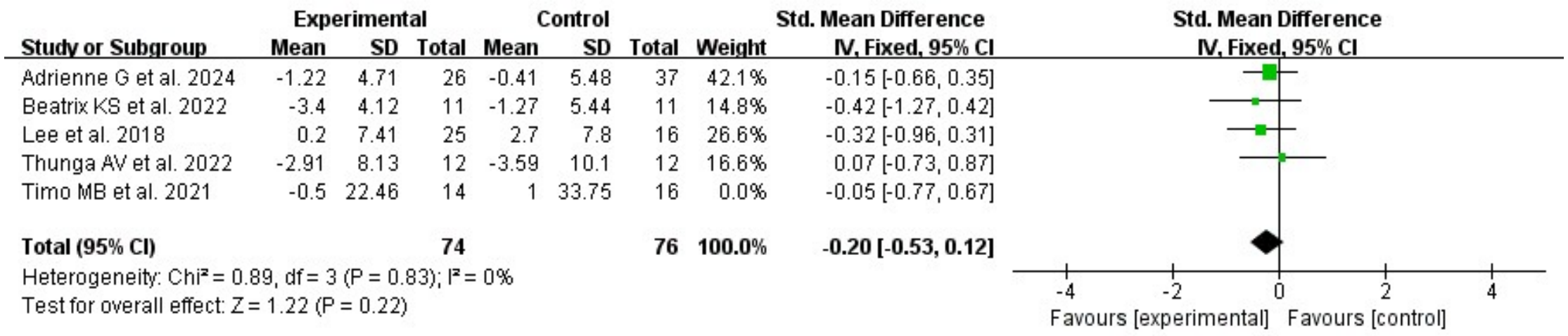

eFigure 7. shows the sensitivity analysis of PDSS

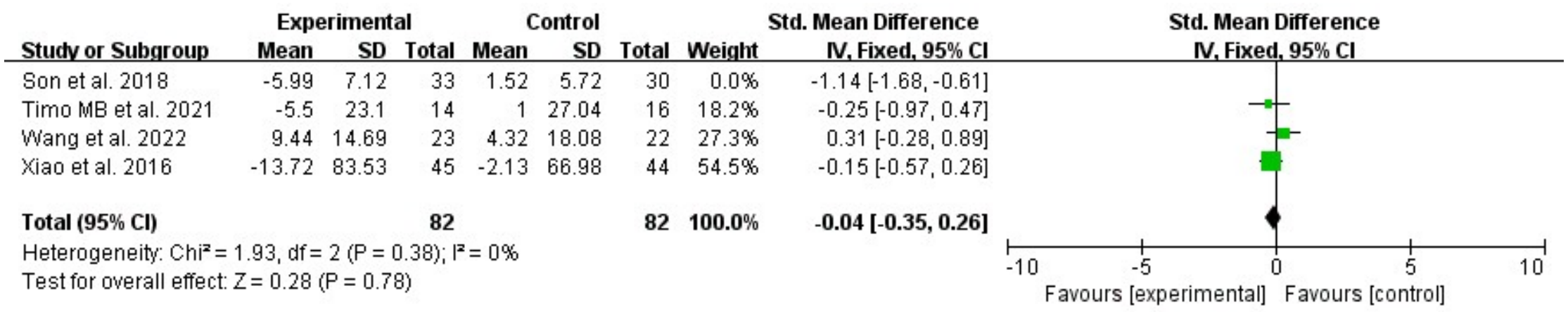

Study or Subgroup	Experimental Mean	Experimental SD	Experimental Total	Control Mean	Control SD	Control Total	Weight	Std. Mean Difference IV, Random, 95% CI
Son et al. 2018	-5.99	7.12	33	1.52	5.72	30	33.0%	-1.14 [-1.68, -0.61]
Timo MB et al. 2021	-5.5	23.1	14	1	27.04	16	0.0%	-0.25 [-0.97, 0.47]
Wang et al. 2022	9.44	14.69	23	4.32	18.08	22	31.9%	0.31 [-0.28, 0.89]
Xiao et al. 2016	-13.72	83.53	45	-2.13	66.98	44	35.1%	-0.15 [-0.57, 0.26]
**Total (95% CI)**			**101**			**96**	**100.0%**	**-0.33 [-1.11, 0.45]**

Heterogeneity: Tau² = 0.41; Chi² = 14.04, df = 2 (P = 0.0009); I² = 86%
Test for overall effect: Z = 0.83 (P = 0.40)

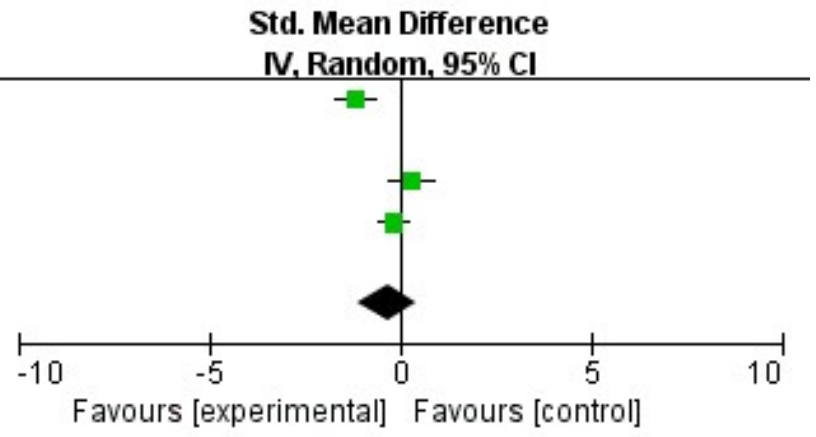

Study or Subgroup	Experimental Mean	Experimental SD	Experimental Total	Control Mean	Control SD	Control Total	Weight	Std. Mean Difference IV, Random, 95% CI
Son et al. 2018	-5.99	7.12	33	1.52	5.72	30	34.0%	-1.14 [-1.68, -0.61]
Timo MB et al. 2021	-5.5	23.1	14	1	27.04	16	28.7%	-0.25 [-0.97, 0.47]
Wang et al. 2022	9.44	14.69	23	4.32	18.08	22	0.0%	0.31 [-0.28, 0.89]
Xiao et al. 2016	-13.72	83.53	45	-2.13	66.98	44	37.3%	-0.15 [-0.57, 0.26]
**Total (95% CI)**			**92**			**90**	**100.0%**	**-0.52 [-1.17, 0.14]**

Heterogeneity: Tau² = 0.26; Chi² = 8.66, df = 2 (P = 0.01); I² = 77%
Test for overall effect: Z = 1.54 (P = 0.12)

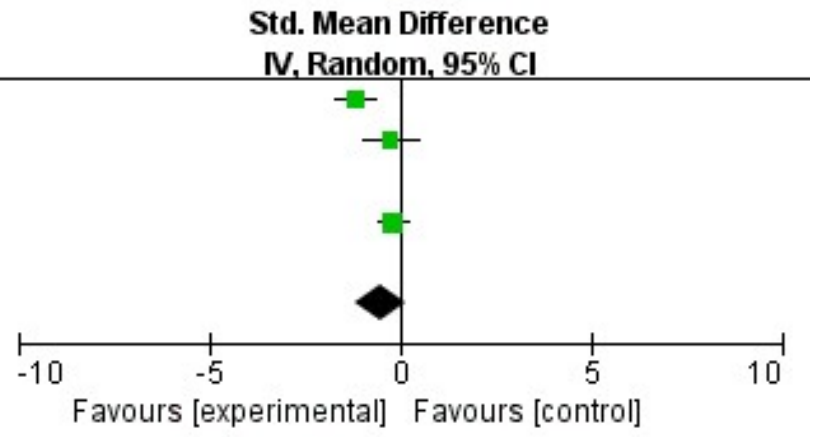

Study or Subgroup	Experimental Mean	Experimental SD	Experimental Total	Control Mean	Control SD	Control Total	Weight	Std. Mean Difference IV, Random, 95% CI
Son et al. 2018	-5.99	7.12	33	1.52	5.72	30	34.7%	-1.14 [-1.68, -0.61]
Timo MB et al. 2021	-5.5	23.1	14	1	27.04	16	31.5%	-0.25 [-0.97, 0.47]
Wang et al. 2022	9.44	14.69	23	4.32	18.08	22	33.8%	0.31 [-0.28, 0.89]
Xiao et al. 2016	-13.72	83.53	45	-2.13	66.98	44	0.0%	-0.15 [-0.57, 0.26]
**Total (95% CI)**			**70**			**68**	**100.0%**	**-0.37 [-1.27, 0.53]**

Heterogeneity: Tau² = 0.53; Chi² = 13.05, df = 2 (P = 0.001); I² = 85%
Test for overall effect: Z = 0.81 (P = 0.42)

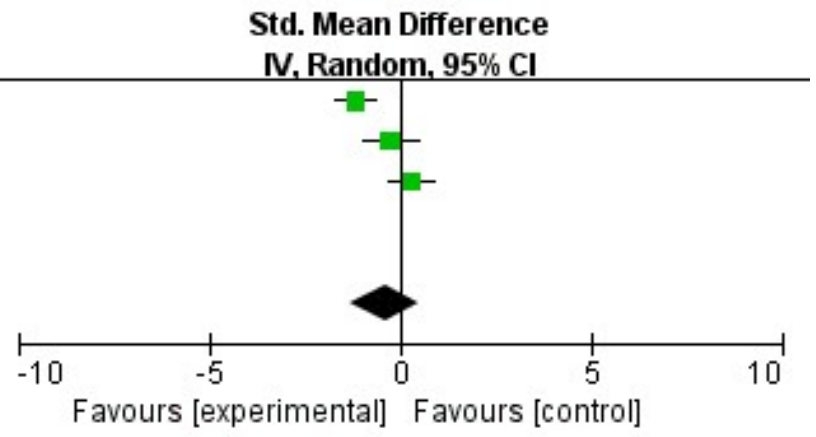

eFigure 8. shows the sensitivity analysis of ADL

Study or Subgroup	Experimental Mean	Experimental SD	Experimental Total	Control Mean	Control SD	Control Total	Weight	Mean Difference IV, Random, 95% CI
Choi et al. 2016	-2.09	2.92	11	0.33	3.66	9	0.0%	-2.42 [-5.37, 0.53]
Joe RN et al. 2013	-5	21.31	15	3.5	26.49	6	1.6%	-8.50 [-32.28, 15.28]
Lee et al. 2018	-0.3	3.9	25	1	2.72	16	42.9%	-1.30 [-3.33, 0.73]
Son et al. 2018	2.17	0.68	33	0.16	0.02	30	52.7%	2.01 [1.78, 2.24]
Thunga AV et al. 2022	-12.98	25.84	12	-6.91	17.22	12	2.9%	-6.07 [-23.64, 11.50]
**Total (95% CI)**			**85**			**64**	**100.0%**	**0.19 [-2.86, 3.25]**

Heterogeneity: Tau² = 4.60; Chi² = 11.65, df = 3 (P = 0.009); I² = 74%
Test for overall effect: Z = 0.12 (P = 0.90)

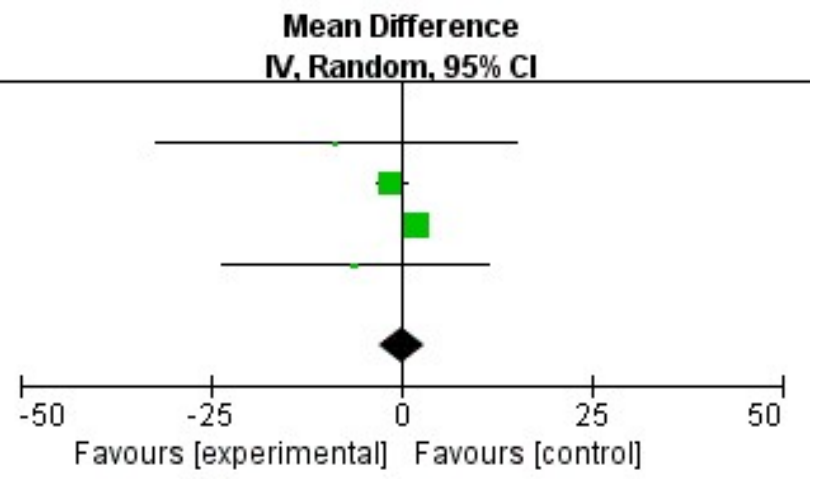

Study or Subgroup	Experimental Mean	Experimental SD	Experimental Total	Control Mean	Control SD	Control Total	Weight	Mean Difference IV, Random, 95% CI
Choi et al. 2016	-2.09	2.92	11	0.33	3.66	9	27.5%	-2.42 [-5.37, 0.53]
Joe RN et al. 2013	-5	21.31	15	3.5	26.49	6	0.0%	-8.50 [-32.28, 15.28]
Lee et al. 2018	-0.3	3.9	25	1	2.72	16	32.1%	-1.30 [-3.33, 0.73]
Son et al. 2018	2.17	0.68	33	0.16	0.02	30	37.8%	2.01 [1.78, 2.24]
Thunga AV et al. 2022	-12.98	25.84	12	-6.91	17.22	12	2.6%	-6.07 [-23.64, 11.50]
**Total (95% CI)**			**81**			**67**	**100.0%**	**-0.48 [-3.43, 2.47]**

Heterogeneity: Tau² = 5.98; Chi² = 19.35, df = 3 (P = 0.0002); I² = 84%
Test for overall effect: Z = 0.32 (P = 0.75)

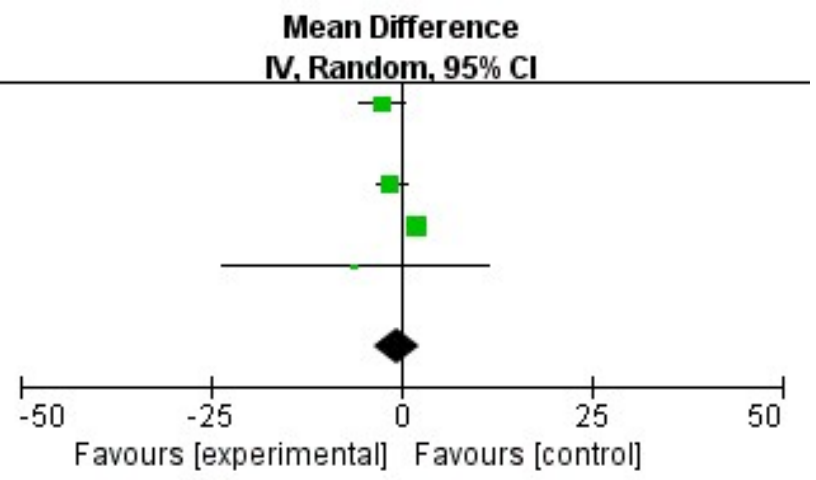

Study or Subgroup	Experimental Mean	Experimental SD	Experimental Total	Control Mean	Control SD	Control Total	Weight	Mean Difference IV, Random, 95% CI
Choi et al. 2016	-2.09	2.92	11	0.33	3.66	9	40.5%	-2.42 [-5.37, 0.53]
Joe RN et al. 2013	-5	21.31	15	3.5	26.49	6	2.6%	-8.50 [-32.28, 15.28]
Lee et al. 2018	-0.3	3.9	25	1	2.72	16	0.0%	-1.30 [-3.33, 0.73]
Son et al. 2018	2.17	0.68	33	0.16	0.02	30	52.2%	2.01 [1.78, 2.24]
Thunga AV et al. 2022	-12.98	25.84	12	-6.91	17.22	12	4.6%	-6.07 [-23.64, 11.50]
**Total (95% CI)**			**71**			**57**	**100.0%**	**-0.44 [-4.40, 3.53]**

Heterogeneity: Tau² = 7.82; Chi² = 10.17, df = 3 (P = 0.02); I² = 71%
Test for overall effect: Z = 0.22 (P = 0.83)

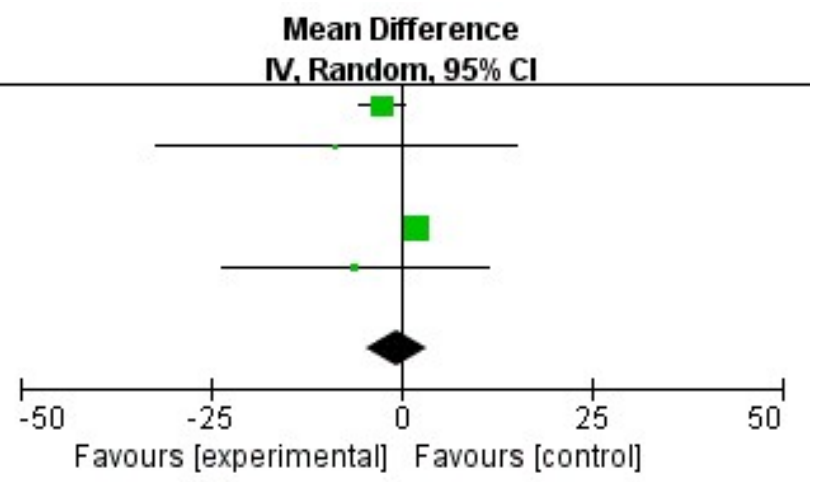

Study or Subgroup	Experimental Mean	Experimental SD	Experimental Total	Control Mean	Control SD	Control Total	Weight	Mean Difference IV, Random, 95% CI
Choi et al. 2016	-2.09	2.92	11	0.33	3.66	9	31.7%	-2.42 [-5.37, 0.53]
Joe RN et al. 2013	-5	21.31	15	3.5	26.49	6	0.5%	-8.50 [-32.28, 15.28]
Lee et al. 2018	-0.3	3.9	25	1	2.72	16	66.9%	-1.30 [-3.33, 0.73]
Son et al. 2018	2.17	0.68	33	0.16	0.02	30	0.0%	2.01 [1.78, 2.24]
Thunga AV et al. 2022	-12.98	25.84	12	-6.91	17.22	12	0.9%	-6.07 [-23.64, 11.50]
**Total (95% CI)**			**63**			**43**	**100.0%**	**-1.73 [-3.39, -0.07]**

Heterogeneity: Tau² = 0.00; Chi² = 0.93, df = 3 (P = 0.82); I² = 0%
Test for overall effect: Z = 2.05 (P = 0.04)

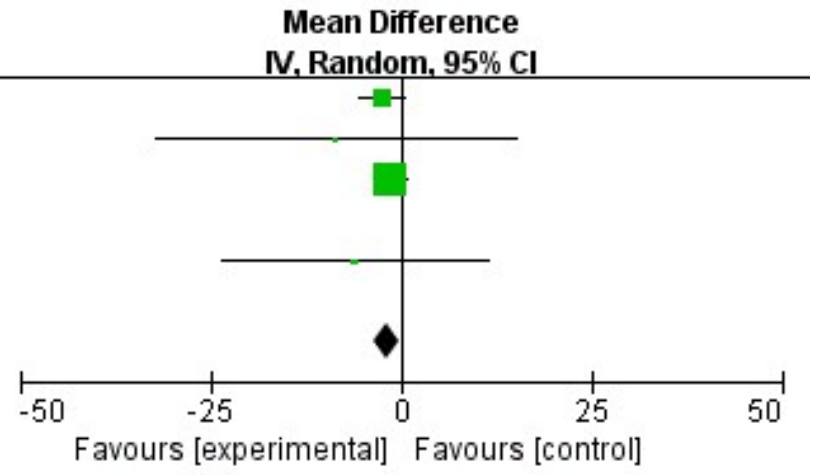

Study or Subgroup	Experimental Mean	Experimental SD	Experimental Total	Control Mean	Control SD	Control Total	Weight	Mean Difference IV, Random, 95% CI
Choi et al. 2016	-2.09	2.92	11	0.33	3.66	9	27.8%	-2.42 [-5.37, 0.53]
Joe RN et al. 2013	-5	21.31	15	3.5	26.49	6	1.5%	-8.50 [-32.28, 15.28]
Lee et al. 2018	-0.3	3.9	25	1	2.72	16	32.5%	-1.30 [-3.33, 0.73]
Son et al. 2018	2.17	0.68	33	0.16	0.02	30	38.2%	2.01 [1.78, 2.24]
Thunga AV et al. 2022	-12.98	25.84	12	-6.91	17.22	12	0.0%	-6.07 [-23.64, 11.50]
**Total (95% CI)**			**84**			**61**	**100.0%**	**-0.45 [-3.42, 2.51]**

Heterogeneity: Tau² = 5.98; Chi² = 19.29, df = 3 (P = 0.0002); I² = 84%
Test for overall effect: Z = 0.30 (P = 0.76)

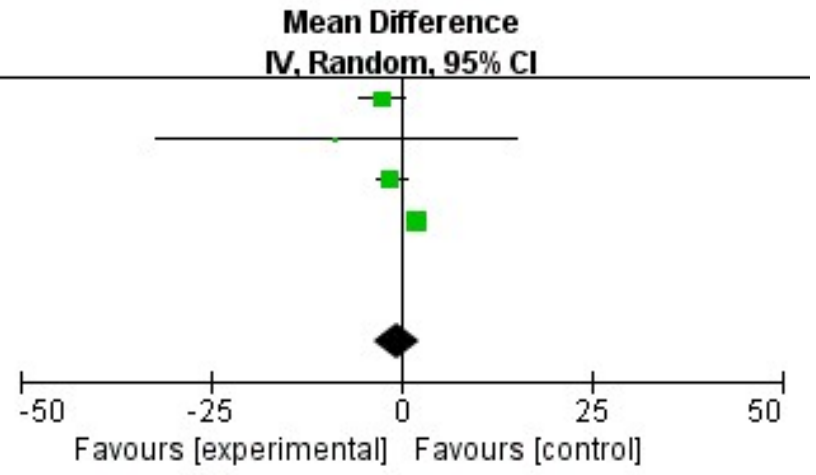

eFigure 9. shows the sensitivity analysis of PDQ-39

Study or Subgroup	Experimental Mean	Experimental SD	Experimental Total	Control Mean	Control SD	Control Total	Weight	Mean Difference IV, Fixed, 95% CI
Gloria VD et al. 2018	4.91	11.1	12	-2.14	11.4	13	0.0%	7.05 [-1.77, 15.87]
Joe RN et al. 2013	1.1	19.92	15	9	29.56	6	2.2%	-7.90 [-33.61, 17.81]
Li et al. 2022a	-7.63	16.6	19	-4.37	19.62	19	11.0%	-3.26 [-14.82, 8.30]
Li et al. 2024	-4	20.01	27	0.11	22.38	27	11.5%	-4.11 [-15.43, 7.21]
Lin et al. 2023	-18.1	10.78	15	-9.5	12.28	15	21.5%	-8.60 [-16.87, -0.33]
Thunga AV et al. 2022	-9.74	15.33	12	-2.47	13.65	12	10.9%	-7.27 [-18.88, 4.34]
Timo MB et al. 2021	-3.94	31.54	14	0.39	53.43	16	1.5%	-4.33 [-35.29, 26.63]
Wang et al. 2022	-5	22.37	23	-1.64	13.75	22	12.6%	-3.36 [-14.16, 7.44]
Yin et al. 2025	-4.82	13.18	25	-2.82	12.93	26	28.6%	-2.00 [-9.17, 5.17]
**Total (95% CI)**			**150**			**143**	**100.0%**	**-4.72 [-8.55, -0.88]**

Heterogeneity: Chi² = 1.78, df = 7 (P = 0.97); I² = 0%
Test for overall effect: Z = 2.41 (P = 0.02)

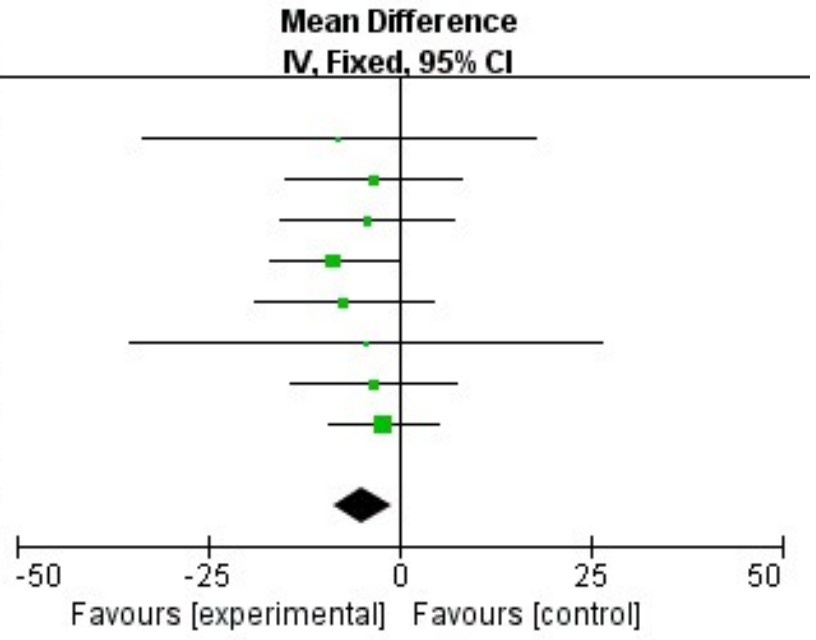

Study or Subgroup	Experimental Mean	Experimental SD	Experimental Total	Control Mean	Control SD	Control Total	Weight	Mean Difference IV, Fixed, 95% CI
Gloria VD et al. 2018	4.91	11.1	12	-2.14	11.4	13	16.2%	7.05 [-1.77, 15.87]
Joe RN et al. 2013	1.1	19.92	15	9	29.56	6	0.0%	-7.90 [-33.61, 17.81]
Li et al. 2022a	-7.63	16.6	19	-4.37	19.62	19	9.4%	-3.26 [-14.82, 8.30]
Li et al. 2024	-4	20.01	27	0.11	22.38	27	9.8%	-4.11 [-15.43, 7.21]
Lin et al. 2023	-18.1	10.78	15	-9.5	12.28	15	18.5%	-8.60 [-16.87, -0.33]
Thunga AV et al. 2022	-9.74	15.33	12	-2.47	13.65	12	9.4%	-7.27 [-18.88, 4.34]
Timo MB et al. 2021	-3.94	31.54	14	0.39	53.43	16	1.3%	-4.33 [-35.29, 26.63]
Wang et al. 2022	-5	22.37	23	-1.64	13.75	22	10.8%	-3.36 [-14.16, 7.44]
Yin et al. 2025	-4.82	13.18	25	-2.82	12.93	26	24.6%	-2.00 [-9.17, 5.17]
**Total (95% CI)**			**147**			**150**	**100.0%**	**-2.75 [-6.30, 0.80]**

Heterogeneity: Chi² = 7.37, df = 7 (P = 0.39); I² = 5%
Test for overall effect: Z = 1.52 (P = 0.13)

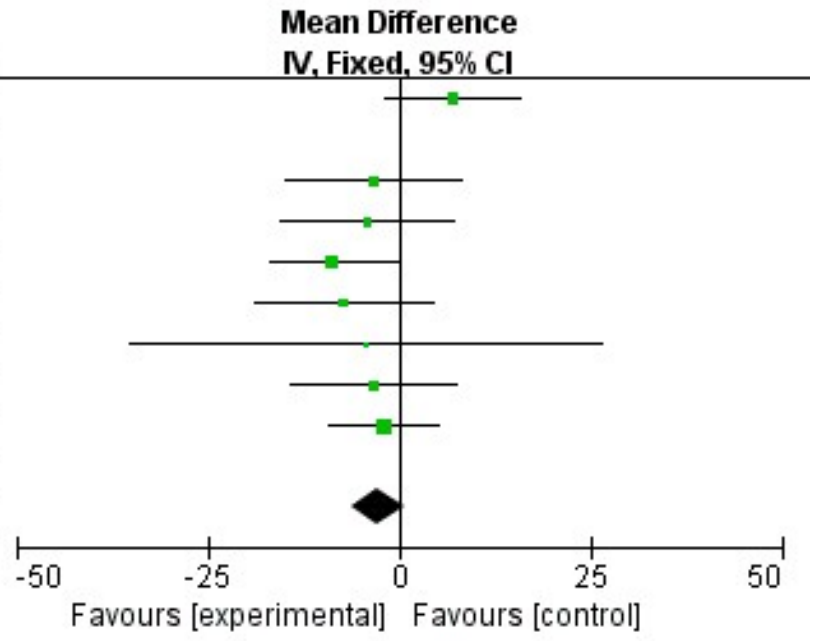

Study or Subgroup	Experimental Mean	Experimental SD	Experimental Total	Control Mean	Control SD	Control Total	Weight	Mean Difference IV, Fixed, 95% CI
Gloria VD et al. 2018	4.91	11.1	12	-2.14	11.4	13	17.5%	7.05 [-1.77, 15.87]
Joe RN et al. 2013	1.1	19.92	15	9	29.56	6	2.1%	-7.90 [-33.61, 17.81]
Li et al. 2022a	-7.63	16.6	19	-4.37	19.62	19	0.0%	-3.26 [-14.82, 8.30]
Li et al. 2024	-4	20.01	27	0.11	22.38	27	10.6%	-4.11 [-15.43, 7.21]
Lin et al. 2023	-18.1	10.78	15	-9.5	12.28	15	20.0%	-8.60 [-16.87, -0.33]
Thunga AV et al. 2022	-9.74	15.33	12	-2.47	13.65	12	10.1%	-7.27 [-18.88, 4.34]
Timo MB et al. 2021	-3.94	31.54	14	0.39	53.43	16	1.4%	-4.33 [-35.29, 26.63]
Wang et al. 2022	-5	22.37	23	-1.64	13.75	22	11.7%	-3.36 [-14.16, 7.44]
Yin et al. 2025	-4.82	13.18	25	-2.82	12.93	26	26.6%	-2.00 [-9.17, 5.17]
**Total (95% CI)**			**143**			**137**	**100.0%**	**-2.80 [-6.50, 0.89]**

Heterogeneity: Chi² = 7.52, df = 7 (P = 0.38); I² = 7%
Test for overall effect: Z = 1.49 (P = 0.14)

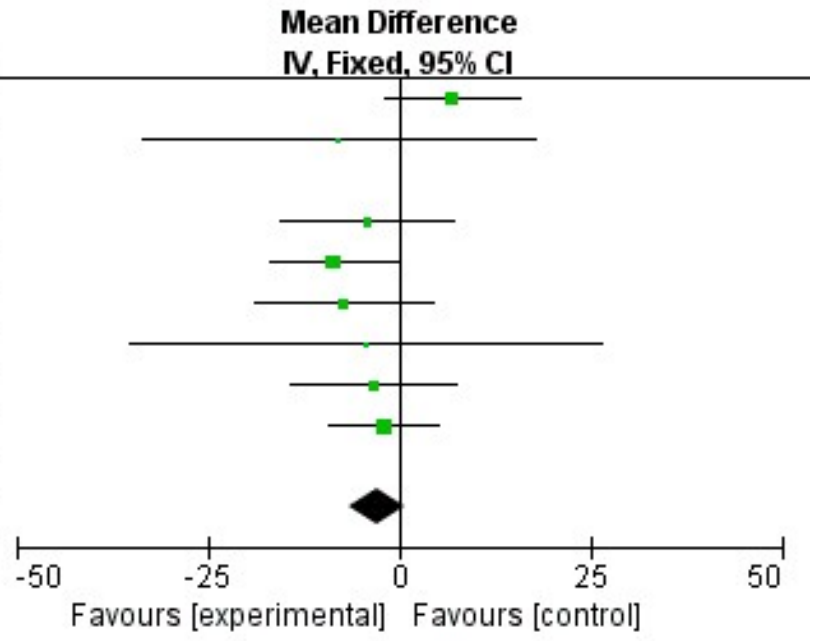

Study or Subgroup	Experimental Mean	Experimental SD	Experimental Total	Control Mean	Control SD	Control Total	Weight	Mean Difference IV, Fixed, 95% CI
Gloria VD et al. 2018	4.91	11.1	12	-2.14	11.4	13	17.6%	7.05 [-1.77, 15.87]
Joe RN et al. 2013	1.1	19.92	15	9	29.56	6	2.1%	-7.90 [-33.61, 17.81]
Li et al. 2022a	-7.63	16.6	19	-4.37	19.62	19	10.3%	-3.26 [-14.82, 8.30]
Li et al. 2024	-4	20.01	27	0.11	22.38	27	0.0%	-4.11 [-15.43, 7.21]
Lin et al. 2023	-18.1	10.78	15	-9.5	12.28	15	20.0%	-8.60 [-16.87, -0.33]
Thunga AV et al. 2022	-9.74	15.33	12	-2.47	13.65	12	10.2%	-7.27 [-18.88, 4.34]
Timo MB et al. 2021	-3.94	31.54	14	0.39	53.43	16	1.4%	-4.33 [-35.29, 26.63]
Wang et al. 2022	-5	22.37	23	-1.64	13.75	22	11.8%	-3.36 [-14.16, 7.44]
Yin et al. 2025	-4.82	13.18	25	-2.82	12.93	26	26.7%	-2.00 [-9.17, 5.17]
**Total (95% CI)**			**135**			**129**	**100.0%**	**-2.71 [-6.41, 0.99]**

Heterogeneity: Chi² = 7.47, df = 7 (P = 0.38); I² = 6%
Test for overall effect: Z = 1.43 (P = 0.15)

Mean Difference IV, Fixed, 95% CI

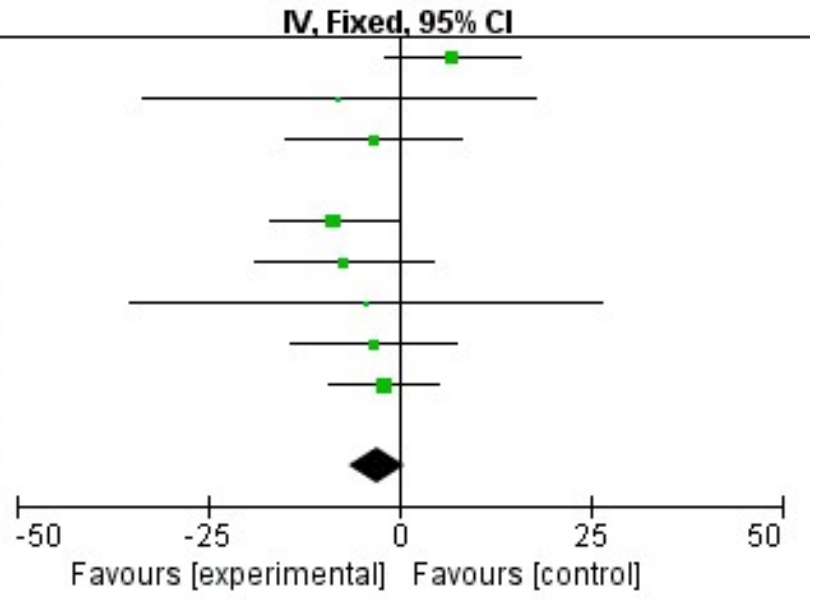

Study or Subgroup	Experimental Mean	Experimental SD	Experimental Total	Control Mean	Control SD	Control Total	Weight	Mean Difference IV, Fixed, 95% CI
Gloria VD et al. 2018	4.91	11.1	12	-2.14	11.4	13	19.4%	7.05 [-1.77, 15.87]
Joe RN et al. 2013	1.1	19.92	15	9	29.56	6	2.3%	-7.90 [-33.61, 17.81]
Li et al. 2022a	-7.63	16.6	19	-4.37	19.62	19	11.3%	-3.26 [-14.82, 8.30]
Li et al. 2024	-4	20.01	27	0.11	22.38	27	11.8%	-4.11 [-15.43, 7.21]
Lin et al. 2023	-18.1	10.78	15	-9.5	12.28	15	0.0%	-8.60 [-16.87, -0.33]
Thunga AV et al. 2022	-9.74	15.33	12	-2.47	13.65	12	11.2%	-7.27 [-18.88, 4.34]
Timo MB et al. 2021	-3.94	31.54	14	0.39	53.43	16	1.6%	-4.33 [-35.29, 26.63]
Wang et al. 2022	-5	22.37	23	-1.64	13.75	22	13.0%	-3.36 [-14.16, 7.44]
Yin et al. 2025	-4.82	13.18	25	-2.82	12.93	26	29.4%	-2.00 [-9.17, 5.17]
**Total (95% CI)**			**147**			**141**	**100.0%**	**-1.57 [-5.46, 2.32]**

Heterogeneity: Chi² = 5.25, df = 7 (P = 0.63); I² = 0%
Test for overall effect: Z = 0.79 (P = 0.43)

Mean Difference IV, Fixed, 95% CI

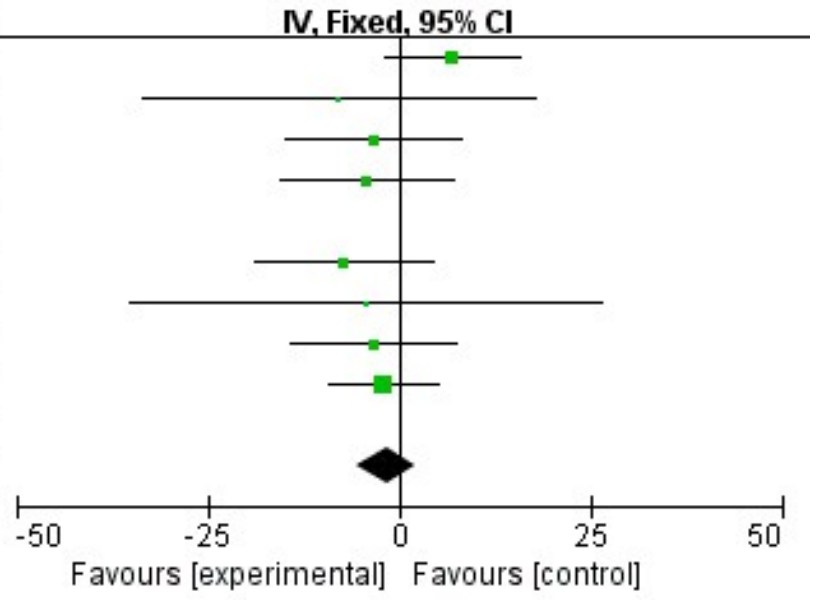

Study or Subgroup	Experimental Mean	Experimental SD	Experimental Total	Control Mean	Control SD	Control Total	Weight	Mean Difference IV, Fixed, 95% CI
Gloria VD et al. 2018	4.91	11.1	12	-2.14	11.4	13	17.5%	7.05 [-1.77, 15.87]
Joe RN et al. 2013	1.1	19.92	15	9	29.56	6	2.1%	-7.90 [-33.61, 17.81]
Li et al. 2022a	-7.63	16.6	19	-4.37	19.62	19	10.2%	-3.26 [-14.82, 8.30]
Li et al. 2024	-4	20.01	27	0.11	22.38	27	10.6%	-4.11 [-15.43, 7.21]
Lin et al. 2023	-18.1	10.78	15	-9.5	12.28	15	19.9%	-8.60 [-16.87, -0.33]
Thunga AV et al. 2022	-9.74	15.33	12	-2.47	13.65	12	0.0%	-7.27 [-18.88, 4.34]
Timo MB et al. 2021	-3.94	31.54	14	0.39	53.43	16	1.4%	-4.33 [-35.29, 26.63]
Wang et al. 2022	-5	22.37	23	-1.64	13.75	22	11.7%	-3.36 [-14.16, 7.44]
Yin et al. 2025	-4.82	13.18	25	-2.82	12.93	26	26.5%	-2.00 [-9.17, 5.17]
**Total (95% CI)**			**150**			**144**	**100.0%**	**-2.40 [-6.09, 1.29]**

Heterogeneity: Chi² = 6.91, df = 7 (P = 0.44); I² = 0%
Test for overall effect: Z = 1.27 (P = 0.20)

Mean Difference IV, Fixed, 95% CI

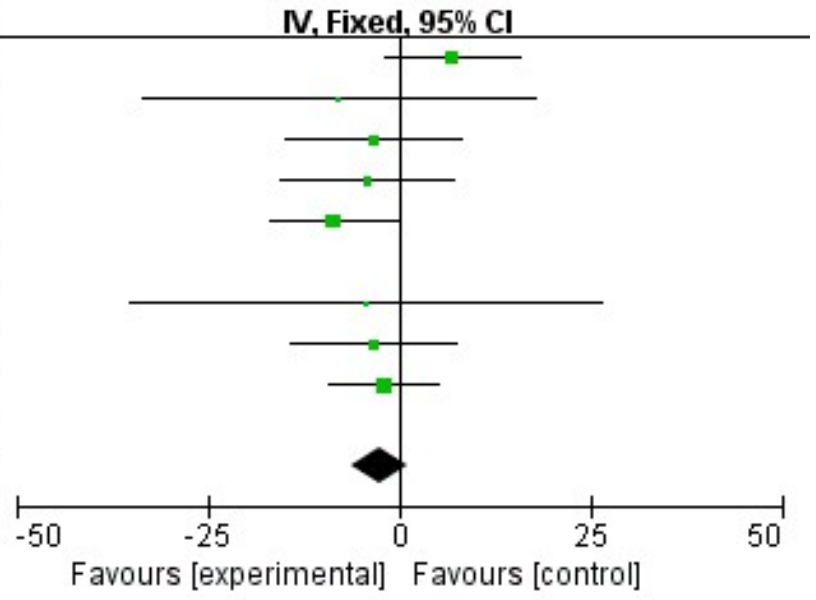

Study or Subgroup	Experimental Mean	Experimental SD	Experimental Total	Control Mean	Control SD	Control Total	Weight	Mean Difference IV, Fixed, 95% CI
Gloria VD et al. 2018	4.91	11.1	12	-2.14	11.4	13	16.1%	7.05 [-1.77, 15.87]
Joe RN et al. 2013	1.1	19.92	15	9	29.56	6	1.9%	-7.90 [-33.61, 17.81]
Li et al. 2022a	-7.63	16.6	19	-4.37	19.62	19	9.4%	-3.26 [-14.82, 8.30]
Li et al. 2024	-4	20.01	27	0.11	22.38	27	9.8%	-4.11 [-15.43, 7.21]
Lin et al. 2023	-18.1	10.78	15	-9.5	12.28	15	18.3%	-8.60 [-16.87, -0.33]
Thunga AV et al. 2022	-9.74	15.33	12	-2.47	13.65	12	9.3%	-7.27 [-18.88, 4.34]
Timo MB et al. 2021	-3.94	31.54	14	0.39	53.43	16	0.0%	-4.33 [-35.29, 26.63]
Wang et al. 2022	-5	22.37	23	-1.64	13.75	22	10.8%	-3.36 [-14.16, 7.44]
Yin et al. 2025	-4.82	13.18	25	-2.82	12.93	26	24.4%	-2.00 [-9.17, 5.17]
**Total (95% CI)**			**148**			**140**	**100.0%**	**-2.83 [-6.37, 0.72]**

Heterogeneity: Chi² = 7.51, df = 7 (P = 0.38); I² = 7%
Test for overall effect: Z = 1.56 (P = 0.12)

Mean Difference IV, Fixed, 95% CI

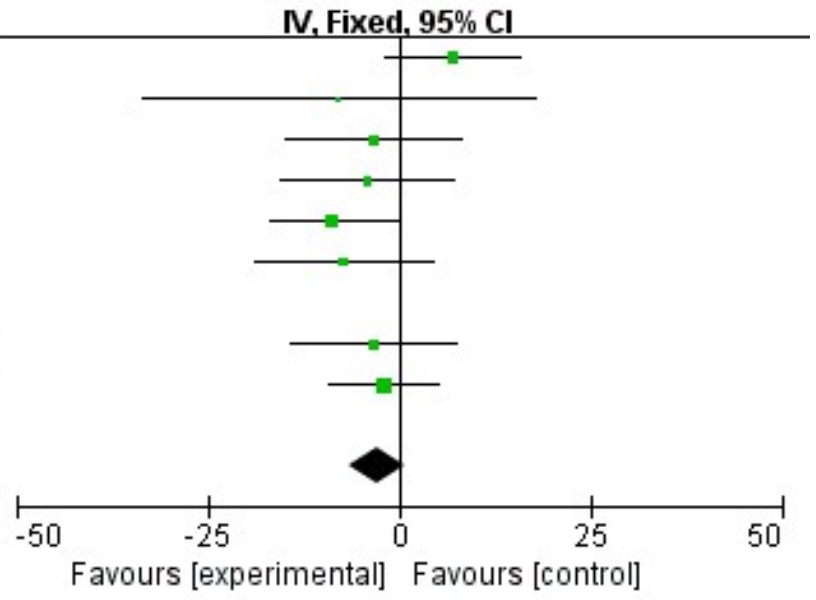

Study or Subgroup	Experimental Mean	Experimental SD	Experimental Total	Control Mean	Control SD	Control Total	Weight	Mean Difference IV, Fixed, 95% CI
Gloria VD et al. 2018	4.91	11.1	12	-2.14	11.4	13	17.8%	7.05 [-1.77, 15.87]
Joe RN et al. 2013	1.1	19.92	15	9	29.56	6	2.1%	-7.90 [-33.61, 17.81]
Li et al. 2022a	-7.63	16.6	19	-4.37	19.62	19	10.4%	-3.26 [-14.82, 8.30]
Li et al. 2024	-4	20.01	27	0.11	22.38	27	10.8%	-4.11 [-15.43, 7.21]
Lin et al. 2023	-18.1	10.78	15	-9.5	12.28	15	20.3%	-8.60 [-16.87, -0.33]
Thunga AV et al. 2022	-9.74	15.33	12	-2.47	13.65	12	10.3%	-7.27 [-18.88, 4.34]
Timo MB et al. 2021	-3.94	31.54	14	0.39	53.43	16	1.4%	-4.33 [-35.29, 26.63]
Wang et al. 2022	-5	22.37	23	-1.64	13.75	22	0.0%	-3.36 [-14.16, 7.44]
Yin et al. 2025	-4.82	13.18	25	-2.82	12.93	26	27.0%	-2.00 [-9.17, 5.17]
**Total (95% CI)**			**139**			**134**	**100.0%**	**-2.78 [-6.51, 0.94]**

Heterogeneity: Chi² = 7.51, df = 7 (P = 0.38); I² = 7%
Test for overall effect: Z = 1.47 (P = 0.14)

Mean Difference IV, Fixed, 95% CI

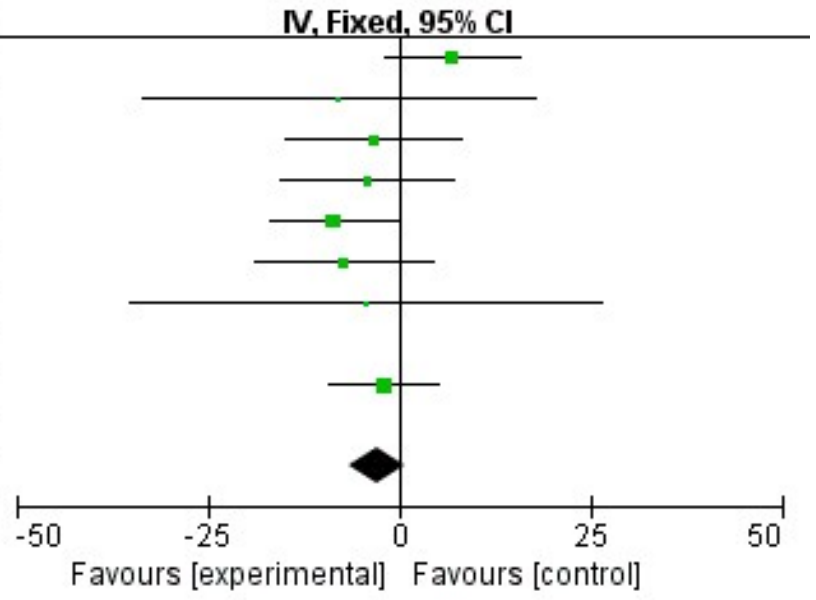

Study or Subgroup	Experimental Mean	SD	Total	Control Mean	SD	Total	Weight	Mean Difference IV, Fixed, 95% CI
Gloria VD et al. 2018	4.91	11.1	12	-2.14	11.4	13	21.0%	7.05 [-1.77, 15.87]
Joe RN et al. 2013	1.1	19.92	15	9	29.56	6	2.5%	-7.90 [-33.61, 17.81]
Li et al. 2022a	-7.63	16.6	19	-4.37	19.62	19	12.2%	-3.26 [-14.82, 8.30]
Li et al. 2024	-4	20.01	27	0.11	22.38	27	12.7%	-4.11 [-15.43, 7.21]
Lin et al. 2023	-18.1	10.78	15	-9.5	12.28	15	23.9%	-8.60 [-16.87, -0.33]
Thunga AV et al. 2022	-9.74	15.33	12	-2.47	13.65	12	12.1%	-7.27 [-18.88, 4.34]
Timo MB et al. 2021	-3.94	31.54	14	0.39	53.43	16	1.7%	-4.33 [-35.29, 26.63]
Wang et al. 2022	-5	22.37	23	-1.64	13.75	22	14.0%	-3.36 [-14.16, 7.44]
Yin et al. 2025	-4.82	13.18	25	-2.82	12.93	26	0.0%	-2.00 [-9.17, 5.17]
**Total (95% CI)**			**137**			**130**	**100.0%**	**-3.11 [-7.15, 0.93]**

Heterogeneity: Chi² = 7.45, df = 7 (P = 0.38); I² = 6%
Test for overall effect: Z = 1.51 (P = 0.13)

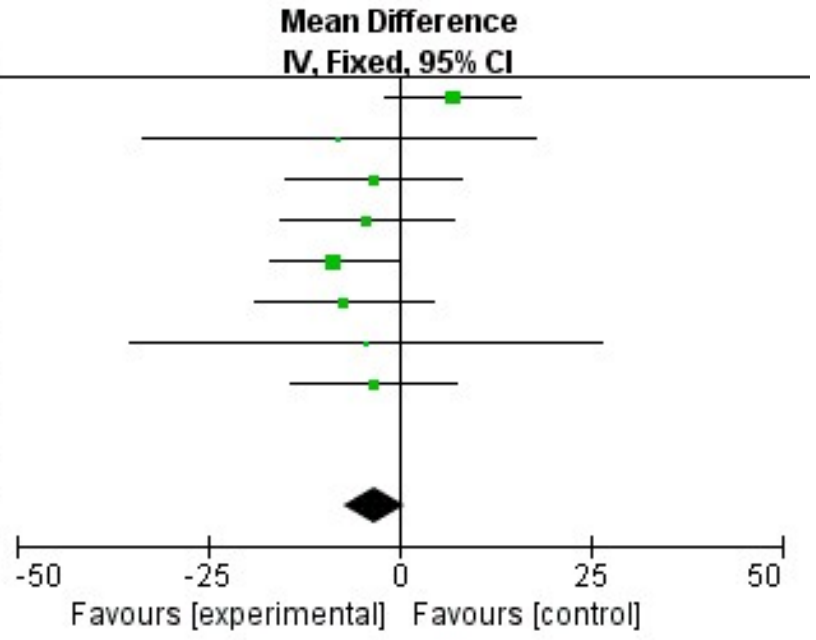

eTable 3 Grade Quality of evidence

Author(s):
Date: 2026-06-19
Question: UPDRS-III for Neurodegenerative Diseases
Settings:
Bibliography: . Mindfulness-Based Exercises for Neurodegenerative Diseases. Cochrane Database of Systematic Reviews [Year], Issue [Issue].

Quality assessment							No of patients		Effect		Quality	Importance
No of studies	Design	Risk of bias	Inconsistency	Indirectness	Imprecision	Other considerations	UPDRS-III	Control	Relative (95% CI)	Absolute		
UPDRS-III (Better indicated by lower values)												
18	randomised trials	serious[1]	no serious inconsistency	no serious indirectness	no serious imprecision	none	506	486	-	MD 4.74 lower (6.78 to 2.7 lower)	⊕⊕⊕O MODERATE	

[1] There's a pretty big bias in how the study assigns the hidden parts

Author(s):
Date: 2026-06-19
Question: TUG for Neurodegenerative Diseases
Settings:
Bibliography: . Mindfulness-Based Exercises for Neurodegenerative Diseases. Cochrane Database of Systematic Reviews [Year], Issue [Issue].

Quality assessment							No of patients		Effect		Quality	Importance
No of studies	Design	Risk of bias	Inconsistency	Indirectness	Imprecision	Other considerations	TUG	Control	Relative (95% CI)	Absolute		
TUG (Better indicated by lower values)												
14	randomised trials	serious[1]	no serious inconsistency	no serious indirectness	no serious imprecision	none	390	367	-	MD 1.63 lower (2.41 to 0.85 lower)	⊕⊕⊕O MODERATE	

[1] There's a pretty big bias in how the study assigns the hidden parts

Author(s):
Date: 2026-06-19
Question: BBS for Neurodegenerative Diseases
Settings:
Bibliography: . Mindfulness-Based Exercises for Neurodegenerative Diseases. Cochrane Database of Systematic Reviews [Year], Issue [Issue].

Quality assessment							No of patients		Effect		Quality	Importance
No of studies	Design	Risk of bias	Inconsistency	Indirectness	Imprecision	Other considerations	BBS	Control	Relative (95% CI)	Absolute		
BBS (Better indicated by lower values)												
6	randomised trials	serious[1]	no serious inconsistency	no serious indirectness	no serious imprecision	none	167	141	-	MD 2.8 higher (1.54 to 4.06 higher)	⊕⊕⊕O MODERATE	

[1] There's a pretty big bias in how the study assigns the hidden parts

Author(s):
Date: 2026-06-19
Question: MoCA for Neurodegenerative Diseases
Settings:
Bibliography: . Mindfulness-Based Exercises for Neurodegenerative Diseases. Cochrane Database of Systematic Reviews [Year], Issue [Issue].

Quality assessment							No of patients		Effect		Quality	Importance
No of studies	Design	Risk of bias	Inconsistency	Indirectness	Imprecision	Other considerations	MoCA	Control	Relative (95% CI)	Absolute		
MoCA (Better indicated by lower values)												
4	randomised trials	serious[1]	no serious inconsistency	no serious indirectness	no serious imprecision	none	107	105	-	MD 1.93 higher (1.12 to 2.74 higher)	⊕⊕⊕O MODERATE	

[1] The sample size is pretty small

Author(s):
Date: 2026-06-19
Question: MMSE for Neurodegenerative Diseases
Settings:
Bibliography: . Mindfulness-Based Exercises for Neurodegenerative Diseases. Cochrane Database of Systematic Reviews [Year], Issue [Issue].

Quality assessment							No of patients		Effect		Quality	Importance
No of studies	Design	Risk of bias	Inconsistency	Indirectness	Imprecision	Other considerations	MMSE	Control	Relative (95% CI)	Absolute		
MMSE (Better indicated by lower values)												
4	randomised trials	serious[1]	no serious inconsistency	no serious indirectness	no serious imprecision	none	103	92	-	MD 2.8 higher (0.32 to 5.29 higher)	⊕⊕⊕O MODERATE	

[1] The sample size is pretty small

Author(s):
Date: 2026-06-19
Question: BDI for Neurodegenerative Diseases
Settings:
Bibliography: . Mindfulness-Based Exercises for Neurodegenerative Diseases. Cochrane Database of Systematic Reviews [Year], Issue [Issue].

Quality assessment							No of patients		Effect		Quality	Importance
No of studies	Design	Risk of bias	Inconsistency	Indirectness	Imprecision	Other considerations	BDI	Control	Relative (95% CI)	Absolute		
BDI (Better indicated by lower values)												
5	randomised trials	serious[1]	no serious inconsistency	no serious indirectness	serious[2]	none	88	92	-	SMD 0.18 lower (0.47 lower to 0.12 higher)	⊕⊕OO LOW	

[1] There's a pretty big bias in how the study assigns the hidden parts
[2] The confidence interval is pretty wide

Author(s):
Date: 2026-06-19
Question: PDSS for Neurodegenerative Diseases
Settings:
Bibliography: . Mindfulness-Based Exercises for Neurodegenerative Diseases. Cochrane Database of Systematic Reviews [Year], Issue [Issue].

Quality assessment							No of patients		Effect		Quality	Importance
No of studies	Design	Risk of bias	Inconsistency	Indirectness	Imprecision	Other considerations	PDSS	Control	Relative (95% CI)	Absolute		
PDSS (Better indicated by lower values)												
4	randomised trials	serious[1]	no serious inconsistency	no serious indirectness	serious[2]	none	115	112	-	SMD 0.31 lower (0.91 lower to 0.28 higher)	⊕⊕OO LOW	

[1] There's a pretty big bias in how the study assigns the hidden parts
[2] The confidence interval is pretty wide

Author(s):
Date: 2026-06-19
Question: ADL for Neurodegenerative Diseases
Settings:
Bibliography: . Mindfulness-Based Exercises for Neurodegenerative Diseases. Cochrane Database of Systematic Reviews [Year], Issue [Issue].

Quality assessment							No of patients		Effect		Quality	Importance
No of studies	Design	Risk of bias	Inconsistency	Indirectness	Imprecision	Other considerations	ADL	Control	Relative (95% CI)	Absolute		
ADL (Better indicated by lower values)												
5	randomised trials	serious[1]	no serious inconsistency	no serious indirectness	serious[2]	none	96	73	-	MD 0.59 lower (3.49 lower to 2.31 higher)	⊕⊕OO LOW	

[1] There's a pretty big bias in how the study assigns the hidden parts
[2] The confidence interval is pretty wide

Author(s):
Date: 2026-06-19
Question: PDQ-39 for Neurodegenerative Diseases
Settings:
Bibliography: . Mindfulness-Based Exercises for Neurodegenerative Diseases. Cochrane Database of Systematic Reviews [Year], Issue [Issue].

Quality assessment							No of patients		Effect		Quality	Importance
No of studies	Design	Risk of bias	Inconsistency	Indirectness	Imprecision	Other considerations	PDQ-39	Control	Relative (95% CI)	Absolute		
PDQ-39 (Better indicated by lower values)												
9	randomised trials	serious[1]	no serious inconsistency	no serious indirectness	serious[2]	none	162	156	-	MD 2.85 lower (6.36 lower to 0.67 higher)	⊕⊕OO LOW	

[1] There's a pretty big bias in how the study assigns the hidden parts
[2] The confidence interval is pretty wide
